# Optimal blood pressure target in patients with uncomplicated hypertension: a target trial emulation study

**DOI:** 10.1038/s41467-026-74041-9

**Published:** 2026-06-05

**Authors:** Ran Zhang, Ivan Chun Hang Lam, Louise Emilsson, Feng Sun, Siyan Zhan, Kai Hang Yiu, Daniel Yee Tak Fong, Esther Yee Tak Yu, Celine Sze Ling Chui, Esther Wai Yin Chan, Ian Chi Kei Wong, Cindy Lo Kuen Lam, Goodarz Danaei, Eric Yuk Fai Wan

**Affiliations:** 1 Department of Family Medicine and Primary Care, School of Clinical Medicine, Li Ka Shing Faculty of Medicine, The University of Hong Kong, Hong Kong SAR, China; 2 Pharmaco- and Device Epidemiology, Health Data Sciences, Nuffield Department of Orthopaedics, Rheumatology and Musculoskeletal Sciences, University of Oxford, Oxford, United Kingdom; 3 Department of General Practice & General Practice Research Unit (AFE), Institute of Health and Society, University of Oslo, Oslo, Norway; 4 Vårdcentralen Nysäter and Centre for Clinical Research, County Council of Värmland, Värmland Värmlands Nysäter, Sweden; 5 Department of Medical Epidemiology and Biostatistics, Karolinska Institutet Solna, Sweden; 6 Department of Epidemiology and Biostatistics, School of Public Health, Peking University Beijing, China; 7 Key Laboratory of Epidemiology of Major Diseases (Peking University), Ministry of Education, Beijing, China; 8 Cardiology Division, Department of Medicine, School of Clinical Medicine, Li Ka Shing Faculty of Medicine, The University of Hong Kong, Hong Kong SAR, China; 9 Cardiology Division, Department of Medicine, The University of Hong Kong — Shenzhen Hospital, Shenzhen, China; 10 The Institute of Cardiovascular Science and Medicine, Li Ka Shing Faculty of Medicine, The University of Hong Kong, Hong Kong SAR, China; 11 School of Nursing, Li Ka Shing Faculty of Medicine, The University of Hong Kong SAR, Hong Kong, China; 12 Primary Healthcare Office, Health Bureau, Hong Kong SAR, China; 13 School of Public Health, Li Ka Shing Faculty of Medicine, The University of Hong Kong, Hong Kong SAR, China; 14 Centre for Safe Medication Practice and Research, Department of Pharmacology and Pharmacy, Li Ka Shing Faculty of Medicine, The University of Hong Kong, Hong Kong SAR, China; 15 The University of Hong Kong Shenzhen Institute of Research and Innovation, Hong Kong SAR, China; 16 Aston Pharmacy School, Aston University, Birmingham, United Kingdom; 17 School of Pharmacy, Medical Sciences Division, Macau University of Science and Technology, Macau SAR, China; 18 Advanced Data Analytics for Medical Science Limited, Hong Kong SAR, China; 19 Department of Family Medicine, The University of Hong Kong-Shenzhen Hospital, Shenzhen, China; 20 Department of Global Health and Population, Harvard TH Chan School of Public Health, Boston, Massachusetts USA; 21 CAUSALab, Department of Epidemiology, Harvard TH Chan School of Public Heath, Boston, Massachusetts, USA; 22 Comprehensive Primary Healthcare Collaboratory, Li Ka Shing Faculty of Medicine, The University of Hong Kong, Hong Kong SAR, China

**Keywords:** Hypertension, Cardiology

## Abstract

The limited real-world evidence on the clinical benefits of intensive blood pressure (BP) management demonstrated in randomised controlled trials has led to its poor adoption in primary care settings. Here we conducted a target trial emulation study on 118,271 patients with uncomplicated hypertension to evaluate the effectiveness and safety managed by intensive (below 130/80 mmHg) compared to standard (130-140/80-90 mmHg) BP targets using a territory-wide public healthcare database in Hong Kong. Patients in the intensive blood pressure target group were observed to incur a lower risk of hypertension related complications and all-cause mortality with no significant increased risk of the serious adverse events reported. The findings of this study provide evidence on the clinical benefits of an intensive BP management in real-world settings, supporting the adoption of a BP target of less than 130/80 mmHg in clinical guidelines for the treatment of adult patients with hypertension in primary care.

## Abstract (web summary)

Optimal blood pressure target is the cornerstone in hypertension management. Here the authors evaluated the effectiveness and safety of the intensive blood pressure target <130/80 mmHg in patients with uncomplicated hypertension.

## Introduction

Hypertension is a common cardiovascular disease affecting approximately one third of the global population^1^. Patients with hypertension are at greater risk of developing severe complications such as acute coronary syndrome and end-stage renal disease, which are leading causes of mortality worldwide^2^. Effective control of blood pressure has been widely regarded as an effective clinical strategy in preventing hypertension-related complications^3^.

Early recommendations from existing treatment guidelines have recommended a conservative treatment target of 140/90 mmHg for systolic blood pressure (SBP) and diastolic blood pressure (DBP), respectively^4^. Nonetheless, findings from subsequent randomised controlled trials (RCTs) and meta-analyses have provided evidence supporting a more intensive treatment targeting of SBP below 130 mmHg and even below 120 mm Hg, given the marked reduction in risk of cardiovascular events in patients treated with more intensive management targets^5–10^. For instance, early clinical trials comparing standard and intensive blood pressure targets have reported a 27% and 25% reduction in risk of cardiovascular events and mortality in individuals managed on lower SBP targets^5,7^. The risk reduction of cardiovascular events and mortality was more profound among elderly patients aged 75 years or over, with reductions of 33% and 34%^6^. More recent trials, including Blood Pressure Control Target in Diabetes (BPROAD) and Effects of Intensive Systolic Blood Pressure Lowering Treatment in Reducing Risk of Vascular Events (ESPRIT) trials have further demonstrated comparable benefits in risk reduction of major cardiovascular events in selected patient groups, including patients with diabetes and a history of stroke, supporting the clinical benefits of intensive blood pressure management across patient groups with different underlying health conditions^11,12^. The observed clinical benefits have since led to the adoption of a more intensive blood pressure target recommendations across various guidelines committees, including the American College of Cardiology / American Heart Association (ACC/AHA)^13^ and the European Society of Hypertension (ESH)^14^.

Whilst previous RCTs have demonstrated the clinical benefits of intensive blood pressure management in selected populations, the scarcity of real-world evidence on the benefits and potential adverse effects associated with patients with uncomplicated hypertension has limited the adoption of such a treatment regimen in clinical settings. This study aimed to evaluate the long-term benefits of intensified blood pressure control in reducing hypertension-related complications and all-cause mortality rates. The results can inform clinical decisions on the optimal blood pressure target for patients with uncomplicated hypertension.

## Results

**Fig. 1:**
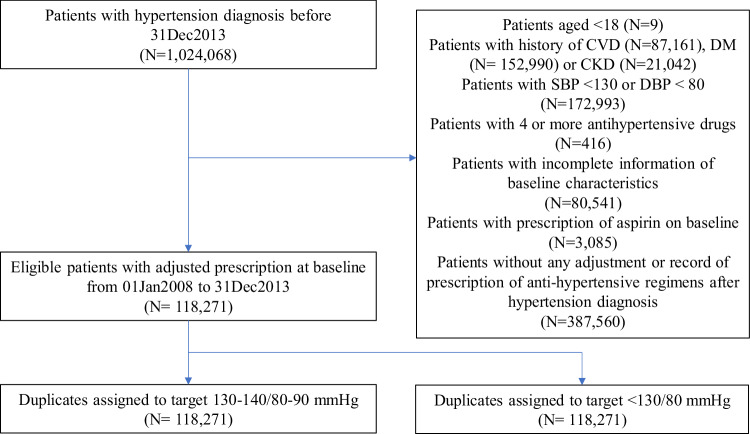
Flowchart of patient selection. Notes: CVD cardiovascular disease; DM diabetes mellitus, CKD:chronic kidney disease, SBP systolic blood pressure, DBP diastolic blood pressure.

**Fig. 2:**
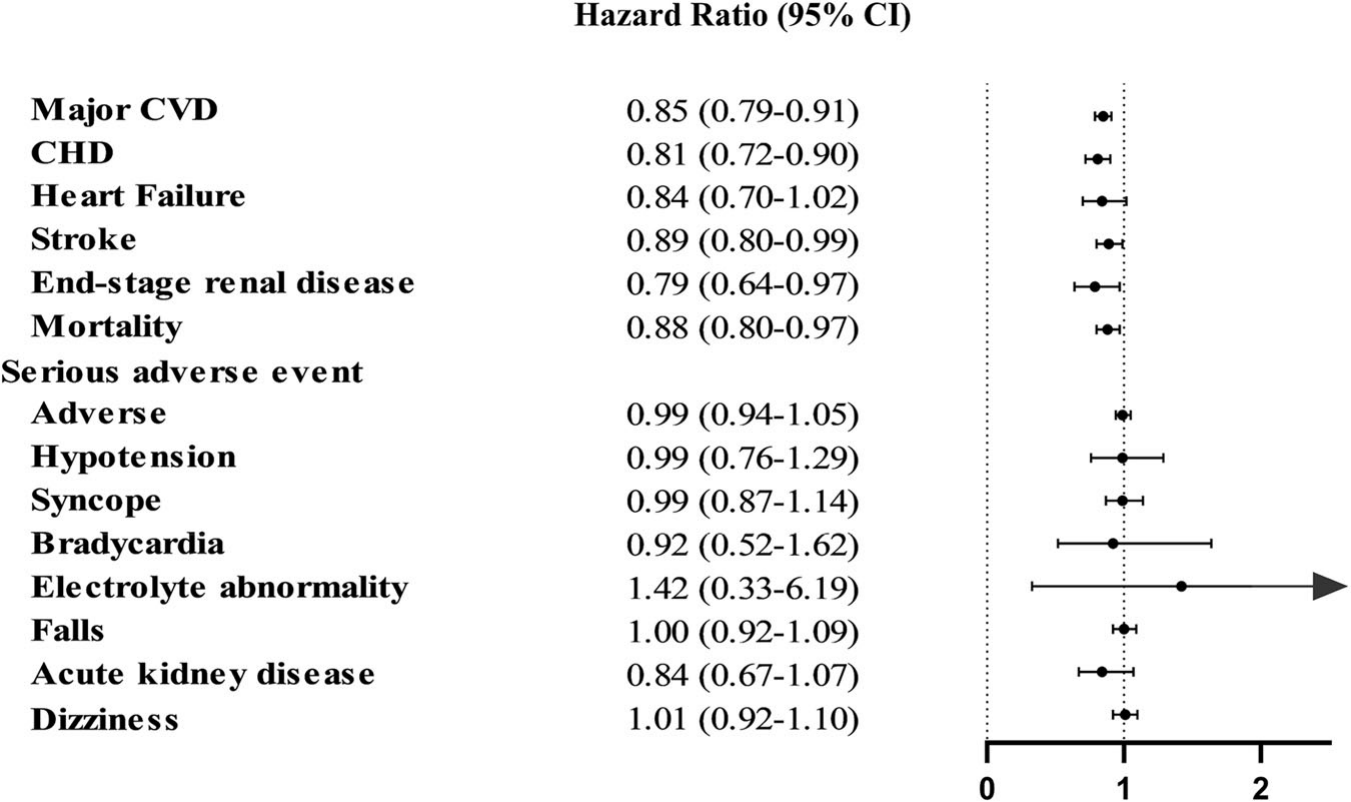
Estimated hazard ratios (and 95% confidence interval) of hypertension-related complications, all-cause mortality and serious adverse events between the intensive treatment target group and the traditional treatment target group. Notes: Major CVD: composite outcomes of heart failure, chronic heart disease and stroke. CHD chronic heart disease, ESRD end-stage renal disease. Analyses adjusted for sex, age, smoking status, fasting glucose, high-density lipoprotein cholesterol, low-density lipoprotein cholesterol, triglyceride, total cholesterol, eGFR, Charlson Comorbidities Index, usage of ACEI/ARB, β-blocker, calcium channel blockers, diuretic; history of adverse events, obesity status, specialist outpatient clinics attendance, general outpatient clinics attendance, accident and emergency attendance, and hospitalization (within 1 year before baseline). Statistical significance was defined as a two-tailed p value. The source data are provided.

A total of 118,271 eligible patients with hypertension were included in this study (Fig. 1). After censoring due to treatment deviation over the grace period, 82,753 patients initiated an optimal BP target of BP 130-140/80–90 mmHg and 15,992 initiated BP of below 130/80 mmHg. Table 1 summarised the baseline characteristics of the participants. The mean age was 57.6 (SD 10.2) and 58.6 (SD 9.7), and BP was 157.1 (16.6)/92.3(8.9) mmHg and 155.1 (15.9)/90.6(8.0) mmHg for patients in the traditional treatment strategy and in the intensive treatment strategy, respectively. Truncation at the 99.5th percentile did not change the overall distribution of the weight (Supplementary Table 4). The crude incidence rates for each outcome were summarised in Table 2. Adherence was around 60% for patients in intensive treatment strategy versus 80% in traditional treatment strategy (Supplementary Fig. 2). Over an average follow-up of 7 years, patients with lower BP target were observed to incur a lower risk of hypertension related complications (Fig. 2), including major CVD [HR (95%CI):0.85 0.79–0.91)], CHD [HR (95%CI): 0.81 (0.72–0.90)], stroke [HR (95%CI): 0.89 (0.80–0.99)], end-stage renal disease [HR (95%CI): 0.79 (0.64–0.97)], and all-cause mortality [HR (95%CI): 0.88 (0.80–0.97)]. The risk reduction (Table 2) was observed for major CVD (absolute risk difference, −0.58 % [95%CI, −0.63 to −0.53 %]), and all-cause mortality (absolute risk difference, −0.60 % [CI, −0.62 to −0.57 %]). There was an average of 35 attended office blood pressure measurements per patient.

**Table 1:** Baseline Characteristics

Demographic	Overall (*N* = 118,271)	SBP 130-140 mmHg and DBP 80–90 mmHg (*N* = 82,753)^a^	SBP < 130 mmHg and DBP < 80 mmHg (*N* = 15,992)^a^
Sex (male)	56,296 (47.6%)	40,005 (48.3%)	6,449 (40.3%)
Age	57.8 (10.4)	57.6 (10.2)	58.6 (9.7)
Smoking status	8119 (6.9%)	5814 (7.0%)	1041.0 (6.5%)
Systolic blood pressure	157.6 (16.8)	157.1 (16.6)	155.1 (15.9)
Diastolic blood pressure	92.2 (8.9)	92.3 (8.9)	90.6 (8.0)
Fasting glucose	5.5 (1.1)	5.5 (1.1)	5.5 (1.1)
Low-density lipoprotein cholesterol	3.3 (0.9)	3.4 (0.9)	3.4 (0.9)
Total cholesterol	5.4 (1.0)	5.4 (1.0)	5.4 (1.0)
High-density lipoprotein cholesterol	1.4 (0.4)	1.4 (0.4)	1.4 (0.4)
Triglyceride level	1.6 (1.1)	1.6 (1.1)	1.5 (1.0)
Body Mass Index	26.1 (4.0)	26.2 (4.0)	25.6 (3.8)
Estimated glomerular filtration rate	87.8 (14.4)	88.0 (14.4)	87.6 (13.9)
Charlson Comorbidity Index	0.1 (0.5)	2.3 (1.2)	2.4 (1.1)
Use of ACEI/ARB	7593 (6.4%)	5117 (6.2%)	859 (5.4%)
Use of β-blocker	19,243 (16.3%)	13,094 (15.8%)	2522 (15.8%)
Use of calcium channel blockers	25,244 (21.3%)	17,711 (21.4%)	2946 (18.4%)
Use of diuretic	7150 (6.0%)	5,038 (6.1%)	874 (5.5%)
Use of other antihypertensive drugs	5101 (4.3%)	3,496 (4.2%)	696 (4.4%)
Use of lipid-lowering agents	21,852 (18.5%)	15,058 (18.2%)	2956 (18.5%)
Dementia	3.5 (3.2)	65 (0.1%)	13 (0.1%)
Doctor consultations in general outpatient clinics^b^	2.6 (1.7)	3.5 (3.1)	3.8 (3.4)
Doctor consultations in specialist outpatient clinics^b^	1.4 (1.3)	2.6 (1.6)	2.5 (1.6)
Accident and emergency services^b^	1.3 (0.8)	1.4 (1.3)	1.4 (1.1)
Inpatient Visit^b^	3.5 (3.2)	1.3 (0.8)	1.3 (0.7)

^a^These numbers do not add up to the total number of patients as some patients deviated over the grace period.

^b^Number of Specialists, General Outpatient Clinics attendance, accident and emergency and hospitalization were counted within 1 year before baseline.

**Table 2:** Crude incidence rate and adjusted risk difference of different blood pressure treatment targets on the risk of hypertension related complications and serious adverse events

	SBP 130–140 mmHg and DBP 80–90 mmHg		SBP < 130 mmHg and DBP < 80 mmHg		
Outcome	Events/Follow-up time	Crude incidence Rate (95% CI)	Events/Follow-up time	Crude incidence Rate (95% CI)	Adjusted Risk Difference (95% CI)
Major CVD	4603 /6.6	8.50 (8.26–8.75)	770 /6.2	7.81 (7.28–8.39)	−0.58 (−0.63, −0.53)
CHD	2188 /6.6	3.99 (3.82–4.16)	362 /6.3	3.63 (3.28–4.03)	−0.23 (−0.26, −0.20)
Heart Failure	741 /6.7	1.34 (1.24–1.44)	109 /6.3	1.08 (0.90–1.31)	−0.15 (−0.18, −0.12)
Stroke	2153 /6.7	3.92 (3.76–4.09)	368 /6.3	3.69 (3.33–4.08)	−0.25 (−0.29, −0.22)
ESRD	73 /6.7	0.13 (0.10–0.17)	7 /6.3	0.07 (0.03–0.15)	−0.55 (−0.57, −0.52)
Mortality	2857 /6.7	5.14 (4.96–5.33)	496 /6.3	4.92 (4.50–5.37)	−0.60 (−0.62, −0.57)
***Serious adverse event***					
Composite of seven serious adverse event	6432 /6.5	12.02 (11.73–12.32)	1212 /6.1	12.47 (11.79–13.19)	−0.15 (−1.64, 1.34)
Hypotension	328 /6.7	0.59 (0.53–0.66)	58 /6.3	0.58 (0.45–0.75)	−0.00 (−0.65, 0.59)
Syncope	1069 /6.7	1.94 (1.82–2.06)	205 /6.3	2.05 (1.78–2.35)	−0.30 (−0.89, 0.28)
Bradycardia	82 /6.7	0.15 (0.12–0.18)	16 /6.3	0.16 (0.10–0.26)	0.03 (−0.03, 0.04)
Electrolyte abnormality	9 /6.7	0.02 (0.01–0.03)	2 /6.3	0.02 (0.00–0.08)	NA*
Falls	2903 /6.6	5.31 (5.12–5.51)	559 /6.2	5.63 (5.18–6.11)	0.11 (−1.20, 1.43)
Acute kidney disease	486 /6.7	0.88 (0.80–0.96)	72 /6.3	0.71 (0.57–0.90)	−0.41 (−1.26, 0.43)
Dizziness	2340 /6.6	4.27 (4.10–4.45)	459 /6.2	4.62 (4.21–5.06)	0.10 (−0.96, 1.16)

Major CVD: composite outcomes of heart failure, chronic heart disease and stroke; *CHD* chronic heart disease, *ESRD* end-stage renal disease. Analyses adjusted for sex, age, smoking status, fasting glucose, high-density lipoprotein cholesterol, low-density lipoprotein cholesterol, triglyceride, total cholesterol, eGFR, Charlson Comorbidities Index, usage of ACEI/ARB, β-blocker, calcium channel blockers, diuretic; history of adverse events, obesity status, specialist outpatient clinics attendance, general outpatient clinics attendance, accident and emergency attendance, and hospitalization (within 1 year before baseline).

*The model can not converge due to the limited number of events.

**Fig. 3:**
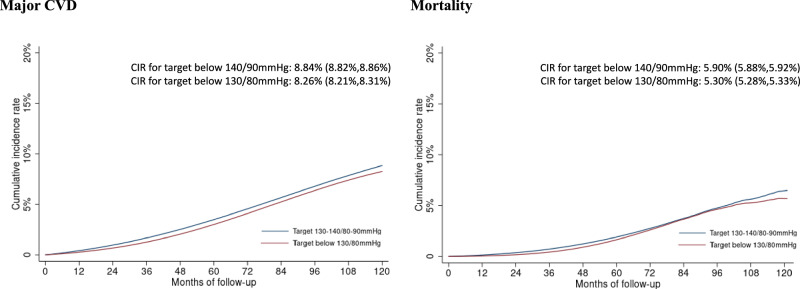
Cumulative incidence of cardiovascular diseases and all-cause mortality. Notes: Analyses adjusted for sex, age, smoking status, fasting glucose, high-density lipoprotein cholesterol, low-density lipoprotein cholesterol, triglyceride, total cholesterol, eGFR, Charlson Comorbidities Index, usage of ACEI/ARB, β-blocker, calcium channel blockers, diuretic; history of adverse events, obesity status, specialist outpatient clinics attendance, general outpatient clinics attendance, accident and emergency attendance, and hospitalization (within 1 year before baseline). A time-specific intercept was also included in the model, incorporating an interaction term between the assigned treatment strategy and the time-specific intercept.

There was no significant increased risk for the serious adverse events requiring hospital admission (Fig. 2) in the lower blood pressure group compared to the traditional treatment target were observed, including falls [HR (95%CI): 1.00 (0.92–1.09) and dizziness [HR (95%CI): 1.01 (0.92–1.10)]. The cumulative incidence curves of major CVD and all-cause mortality were summarised in the Fig. 3. Sensitivity and subgroup analyses reported largely consistent findings for aforementioned outcomes (Supplementary Tables 5–26). Notably, a similar risk-lowering benefit in CVD was observed across patients with varying baseline estimated 10-year CVD risk. The risk reduction effects in CHD associated with intensive blood pressure monitoring targets were more profound among patients aged between 65 and 80 compared to patients from other age subgroups. Despite age not being an interaction term with complications examined in this study, the greater risk of adverse events such as dizziness warrant the need for closer attention in older patients with lower blood pressure target (Supplementary Table 8). The blood pressure levels and proportion of intensification in the two treatment strategies over the follow-up period were shown in the Supplementary Fig. 3 and Supplementary Table 27. The lower blood pressure target group identified more instances of drug escalation or dosage increases, suggesting an intensification of treatment in the lower blood pressure treatment arm. The mean number of anti-hypertensive medications used by patients in each arm by the follow-up year was shown in Supplementary Table 28. The model estimates in the inverse probability weighting were summarized in Supplementary Table 29.

## Discussion

Our study on adults with uncomplicated hypertension reported a lower risk of CVD-related complications, end-stage renal disease, and mortality in patients managed by an intensive blood pressure target of less than 130/80mmHg compared to the standard blood pressure target over an up to 11 years of follow-up. The observed  major CVD and all-cause mortality risk reduction benefits were broadly consistent across various subgroups, supporting the clinical benefits of a lower blood pressure target that applies over a diverse population of patients.

Hypertension has been widely regarded as a major risk factor for CVD due to the potential damage to the vascular and myocardial tissues imposed by the prolonged high blood pressure, resulting in damage to vital organs and atherosclerosis^15^. The cumulative evidence from previous RCTs demonstrating a risk reduction in CVD complications in patients treated with a lower blood pressure target has supported “the lower the better” approach in determining the optimal blood pressure for individual patients. Nonetheless, the limited follow-up period and the recruitment of under-representative study populations have led to disputes over such an approach in clinical settings. For instance, the considerable proportion of elderly participants in the SPRINT trial, comprising over 25% of participants aged over 75, could lead to an over-estimation of the treatment efficacy of the lower blood pressure management target and limit its generalisability across the general population^5,16,17^. Subsequent evidence from a meta-analysis including a broader population of hypertensive patients demonstrated a markedly lower risk of myocardial infraction and stroke in patients managed by a lower blood pressure target compared the standard blood pressure target^18^. Despite the clinical benefits observed, the arbitrary definition of the lower blood pressures of ( < 140/90 and <135/85 mmHg) have led to poor interpretability of its findings in clinical settings^14^. The consistent findings of this study, based on a wider coverage of patients with a wider age range and CVD risk, further emphasised the clinical benefits in preventing CVD complications and all-cause mortality across the general population over the long-term, as well as supporting the adoption of a lower blood pressure target of 130/80 mmHg for patients with uncomplicated hypertension.

Despite the marked reduction in the risk of CVD, existing observational studies and RCTs have reported a significantly increased risk of the adverse events^17,19,20^, including hypotension, electrolyte imbalances, acute kidney diseases and falls associated with aggressive blood pressure management. In contrast, we did not observe among patients managed with an intensive blood pressure target of 130/80mmHg a greater risk of serious adverse effects. Our findings regarding the safety profile are generally consistent with the findings from the latest RCT conducted in China^12^. The low observed incidence of serious adverse events could be partially attributed to the selection of a healthier population. However, the subgroup analyses of patients with a high Charlson Comorbidity Index (CCI) did not indicate an increased risk of serious adverse events, supporting the lack of such risk across a diverse patient group with varying health conditions and comorbidity statuses. The low incidence rates of electrolyte abnormality might be due to the low use of diuretics in our study population, which is consistent with the practice among other Chinese populations^21,22^. Additionally, we excluded the patients with chronic kidney disease, who are more likely to develop acute kidney injury^23^. While there was no significant increase in serious adverse events that requiring hospitalisation associated with intensified blood pressure target <130/80 mmHg reported in our study, the slight increase in risk of syncope and dizziness observed during outpatient consultation implicated that a careful balance of potential benefits and risks of intensive blood pressure control should be considered on individual patients in clinical settings. Future study using more detailed and systematic adverse events monitoring are needed to better evaluate the safety of the intensive blood pressure target.

To our knowledge, this is the first study to investigate the effects of intensive blood pressure targets among the patients with uncomplicated hypertension with varying characteristics in real-world clinical settings. The population-based observational data, with at most 11-year follow-up period, allowed us to reveal the relationship between different optimal blood pressure targets and long-term health effects adjusted by the major potential confounders. In addition, the emulated RCT design used as the analytical approach on the per-protocol effect, where the evolution of the post-baseline confounders was adjusted. Furthermore, the patients with younger age and lower CVD risks were included in our study compared to the previous trials, extending the current evidence on the benefits of a lower blood pressure target in all populations for the primary prevention of hypertension-related complications.

However, we acknowledged several limitations in this study. Firstly, the information on the desired blood pressure target was not recorded in the CMS database. We assumed the blood pressure target based on the blood pressure records and the concurrent antihypertensive drugs prescription data, which may have introduced a misclassification of the treatment target groups. Besides, the treatment assignment after the long grace period could potentially introduce further misclassification bias. However, given the prolonged follow-up in this study of over 7 years, the duration of grace period included in relation to the total follow-up period was in-line with previous study employing the clone-censor-weight design^24,25^. Moreover, the results of the sensitivity analysis with a short grace period of six-months and the sensitivity analysis using different numbers of consecutive records of BP readings concurrent with the prescription records have majorly yielded consistent findings, showing that any potential misclassification bias using this definition should not have a major impact on the overall results. Secondly, the target-based definition of the treatment assignment may violate the assumption of consistency since patients can achieve the optimal blood pressure target through treatment modification, but attributed to treatment intensification in this study. Thirdly, some potential confounders, such as hypertension duration, lifestyle factors (including diet and physical activity), socioeconomic status, and educational level, were unavailable in our study, which may have introduced bias to our results. Additionally, despite our efforts to emulate a target trial and account for various clinical scenarios, certain confounders and patients’ complexities in real-world practice may not have been fully captured, similar to other observational studies. Therefore, the findings reported in this study should be interpreted with caution in patients with specific clinical conditions or complexities. Future research incorporating additional real-world variables is warranted to further examine the benefits of intensive BP monitoring in other patient groups. Furthermore, in our dataset, only the primary cause of death was recorded, which may lead to potential misclassification of the cause of death when considering the competing risks of the non-CVD mortality in this study. Further studies with more precise and comprehensive cause-of-death data are necessary to better understand the relationship between intensive blood pressure management and related complications. Lastly, ICPC-2 and ICD-9-CM codes were used to identify the diagnosis in the CMS database, which may lead to misclassification. However, as demonstrated in the previous studies using this database, the history of chronic diseases in the HKHA has been recorded with a high coding accuracy^26,27^.

The findings of the study provided comprehensive real-world evidence supporting the association of the clinical benefits of reduced risk of hypertension-related cardiovascular complications and all-cause mortality with an intensive blood pressure management target among patients with uncomplicated hypertension without a significantly increased risk of serious adverse events. Nevertheless, the optimal blood pressure management target should also take into consideration of individual’s characteristics, comorbidities, and potential risk factors for associated adverse events.

## Methods

This study was approved by the Institutional Review Board of the University of HK/HA HK West Cluster (UW19-361) with an exemption for informed consent from participants since all data used in this study were anonymized and obtained from the electronic health records from the HA.

### Data sources

We conducted a trial emulation using data from the Clinical Management System (CMS) provided by the Hong Kong Hospital Authority (HKHA) to evaluate the effects of different blood pressure targets on the risks of hypertension-related complications and adverse events among patients with hypertension. The HKHA manages all public healthcare services in Hong Kong, China. The service is available to all Hong Kong residents, covering over 80% of all routine healthcare management. The electronic medical records maintained by the HKHA include the disease diagnoses recorded during doctor consultations from in- and out-patient hospital and emergency visits^28^. The blood pressure readings in this data source are from attended office measurements.

### Eligible criteria for study participants

All patients aged≥18 with a documented diagnosis of hypertension on or before December 2013 were included in our study. The baseline was defined as the date of the first prescription adjustment for patients with hypertension whose blood pressure records were≥130/80 mmHg on that date, between 01 January 2008 and 31 December 2013. The treatment adjustment in these patients with uncontrolled blood pressure were regarded as an indication that the clinicians were attempting to modify and monitor their treatment plans to achieve the potential optimal BP target. Patients on 4 or more regular antihypertensive medications within 3 months before baseline, as well as patients with a history of CKD, DM, or CVD, were excluded from this study. Complete case analysis was conducted, and patients with incomplete data for the used covariates at the baseline were also excluded. Individuals with a prescription for aspirin on or before baseline were excluded to avoid the underdiagnosis of CVD history, as aspirin is used primarily for recurrence prevention in patients with cardiovascular diseases^29^. The details of the study design and target trial emulation were summarised in Supplementary Fig. 1 and Supplementary Table 1.

### Treatment strategies

According to the Hong Kong Reference Framework^30^, the optimal blood pressure treatment goal for patients without CKD, DM or CVD is below 140/90 mmHg, and a lower goal can be considered for those individuals who can tolerate. Therefore, two treatment targets were considered for comparison: (1) Continue being treated according to the current Hong Kong Reference Framework^30^: SBP 130-140 mmHg and DBP 80-90 mmHg; (2) a more intensive treatment goal: SBP < 130 mmHg and DBP < 80 mmHg. The initiation of the treatment strategy was defined based on the BP readings and the concurrent prescription records of antihypertensive regimens during 12-month grace period.

Patients with two consecutive records of BP < 130/80 mmHg without de-escalation, or BP readings of 130-140/ 80-90 mmHg with escalation of drug treatment, were considered to be treated to reach the intensive treatment target. The rest of the patients who already achieved the standard blood pressure target were considered to continue treatment according to the current treatment guideline with a target of 130-140/80-90 mmHg. Therefore, patients who did not achieve the traditional treatment target after the grace period, or patients without follow-up after baseline, were censored from the study during the grace period.

### Outcome measures and follow-up period

The outcomes of this study included the incidences of major cardiovascular disease (a composite outcomes of coronary heart disease, heart failure and stroke), coronary heart disease (CHD), stroke, heart failure, end-stage renal disease (ESRD), all-cause mortality and seven serious adverse events of intensive treatment (hypotension, syncope, bradycardia, electrolyte abnormality, fall(s), acute kidney injury or acute renal failure and dizziness). Disease diagnoses were based on the International Classification of Primary Care, 2nd edition (ICPC-2), International Classification of Diseases, 9th Revision, Clinical Modification (ICD-9-CM), or relevant clinical parameters (Supplementary Table 2). The serious adverse events of blood pressure treatment were defined as a diagnosis requiring hospital admission. All patients were followed from the baseline until the outcome events, death or the end of the study (31 Dec 2018), whichever occurred first.

### Statistical analysis

We adopted the cloning, artificial censoring and weighting approach to minimise the potential selection bias and immortal time bias because the treatment strategy of interest includes a grace period of 1 year for initiation of the optimal blood pressure target^31^. Firstly, we created the dataset of the eligible patients at baseline with 2 replicates (clones), and each of the replicates was randomly assigned to one of the treatment strategies. After that, we assessed whether the replicates adhered to their assigned treatment strategy at monthly intervals. To estimate the per-protocol effect of the optimal BP target, participants who actually deviated from their assigned strategy were censored unless the deviation was explained by a medical reason. For example, if replicates were assigned to the traditional treatment strategy, but (1) were not treated with the traditional treatment initially by the end of 12 months, or (2) received the intensive treatment after the grace period (identified as patients with five consecutive records of with BP below 140/90 mmHg with prescription escalation), except for changes due to the diagnosis of DM or CKD, or (3) did not maintain the standard treatment target after the grace period (defined as patients with five consecutive records of BP records which were higher than 140/90 mmHg without prescription escalation), they were censored at that time point; Conversely, if replicates were assigned to the intensive treatment target, but (1) were not treated with the intensive treatment target initially by the end of 12 months, or (2) were treated with the higher BP target after the grace period (defined as patients with five consecutive of BP records below 130/80 mmHg with prescription de-escalation; or of BP records higher than 130/80 without prescription escalation), they were be censored at that time point. The possible censoring mechanisms in our emulated trial setting were illustrated in Supplementary Fig. 1.

A panel dataset was created for all time-varying indicators (by month) for each eligible patient. The last observation carried forward was used for time-varying clinical parameters during the follow-up period. To adjust for the selection bias introduced by the aforementioned artificial censoring process, we conducted the inverse probability weighting to account for the difference in baseline and time-varying covariates, including sex; age; smoking status; fasting glucose level; low-density lipoprotein cholesterol (LDL-C), high-density lipoprotein cholesterol (HDL-C), total cholesterol (TC); triglyceride (TG) level; estimated glomerular filtration rate (eGFR); obesity status; and usage of ACEI/ARB, β-blocker, calcium channel blockers, diuretic, CCI score; history of adverse events; and in the past year the number of attendances for doctor consultations in general outpatient clinics, specialist outpatient clinics, accident and emergency services, and overnight hospitalisation between separate cohorts. The weighted score of individuals at each specific time points were calculated by the sum of the cumulative weighted score of previous time points.

Finally, the cumulative inverse probability weight at each time point was truncated at the 0.5th and 99.5th percentiles. A pooled logistic model was fitted to estimate the hazard ratio for the effect of the continuous blood pressure target on the incidence of outcome events. The indicators for the assigned treatment strategy, month of follow-up (linear and quadratic terms), and baseline covariates were included in the models with time-varying weights.

The 10-year absolute risk difference and cumulative incidence of each outcome were estimated using the aforementioned pooled logistic model with interaction terms between treatment and follow-up time after standardising the outcomes using the joint distribution of the covariates in the entire study population. The 95% CIs for the absolute risk difference and cumulative incidence were obtained from a nonparametric bootstrap with 200 samples.

### Subgroup analysis and sensitivity analysis

Subgroup analyses were predefined taking account of the risk factors of hypertension complications identified. Patients were stratified by 1) age ( < 65, 65–79, ≥80), 2) gender, 3) CCI ( < 4, ≥4), 4) smoking status, 5) CVD risk using Framingham risk score formula ( < 10,10-20 ≥ 20), and 6) obesity status. The interaction between treatment and each subgroup was also evaluated. Several sensitivity analyses were performed as: (1) Applying the same target trial emulation without adjustment for time-varying confounders to estimate the intention-to-treat effect, that is, the effect of being assigned to intensive treatment compared with standard treatment at baseline on the risk of outcomes^32^; (2) Censoring patients with missing SBP records (defined as an interval of more than 1 year between two SBP records) during follow-up to assess the influence of carrying forward the last SBP value for missing data; (3) Changing the grace period from 12 months to 6 months; (4) Using single or three, instead of two, consecutive BP readings and prescription records of antihypertensive regimens to define the treatment strategy, in order to investigate the robustness of the results to the definition of treatment assignment; (5) Evaluating the safety of the treatment target by assessing the incidences of adverse events from both inpatient and outpatient settings; (6) Using cancer as a negative control outcome; (7) Including baseline BP readings in the weighting model; (8) Adjusting for competing risks in the per-protocol analysis: each clone additionally received a time-varying inverse probability weight for not dying of non-cardiovascular events; (9) Including patients with low-dose aspirin prescriptions on or before baseline; (10) Including the year of the enrolment in the weighting model; (11) Using two, three, four, or six consecutive records to define deviation from the assigned treatment strategy after the grace period; (12) Considering adverse effects due to advanced age (defined as older than 65 years), frailty (defined as a change in CCI greater than 2 compared to baseline), and polypharmacy (defined as taking more than three types of drugs) during the follow-up period as contraindications.

All analyses were performed in Stata/MP 17.0. Statistical significance was defined as a two-tailed p-value.

### Reporting summary

Further information on research design is available in the Nature Portfolio Reporting Summary linked to this article.

## Supplementary information

Supplementary Information

Reporting Summary

Transparent Peer Review file

## Source data

Source Data

## Data Availability

The data used in this article were provided by the Hospital Authority of Hong Kong. Due to the data sharing policy, the data containing confidential information cannot be shared with the public. Local academic institutions, government departments, or non-governmental organizations interested in accessing the data may apply through the Hospital Authority’s data-sharing portal (https://www3.ha.org.hk/data).The investigators are responsible for the archiving and safekeeping of the personal and study data during and after the study. Source data are provided with this paper.

## Code availability

The code used in this study is available on Zenodo (10.5281/zenodo.17033442).
