# Supplementary material for: Optimal blood pressure target in patients with uncomplicated hypertension: a target trial emulation study
Source: Nat Commun. 2026 Jun 5;17:7207. doi: 10.1038/s41467-026-74041-9 (PMC13396347; doi:10.1038/s41467-026-74041-9)

Supplementary Table 1 Summary of the study protocol of emulating a target trial of optimal blood pressure targets using electronic health record data

Protocol component	Specification	Emulation using observational data
Eligible criteria	(1) Age ≥ 50<br>(2) Systolic blood pressure<br>SBP: 130 – 180 mm Hg on 0 or 1 medication<br>SBP: 130 – 170 mm Hg on up to 2 medications<br>SBP: 130 – 160 mm Hg on up to 3 medications<br>SBP: 130 – 150 mm Hg on up to 4 medications. [1]<br>(3) One or more of the risks below (increase risk of cardiovascular events):<br>a) Presence of clinical* or subclinical** cardiovascular disease other than stroke<br>b) CKD, defined as eGFR 20 – 59 ml/min/1.73m2 based on the 4-variable<br>Modification of Diet in Renal Disease (MDRD) equation and latest lab value,<br>within the past 6 months. (If the serum creatinine is unstable within the last 6<br>months, enrollment into SPRINT could be delayed until the serum creatinine<br>has been stabilized and the eGFR is still within the allowed range.)<br>c) Framingham Risk Score for 10-year CVD risk ≥ 15% based on laboratory<br>work done within the past 12 months for lipids<br>d) Age ≥ 75 years.<br><br>Exclude patients who:<br>With polycystic kidney disease (PKD)<br>With Diabetes mellitus<br>Have had a stroke<br><br>Baseline is defined as the date of recruitment.	All patients aged≥18 with a documented diagnosis of HT, SBP≥130mmHg and DBP≥80mmHg and prescription record between 1 January and 31 December 2013 recorded in the electronic clinical management system (CMS) of the Hong Kong Hospital Authority (HA) will be included in the target cohort.<br>Exclude patients who:<br>(1) With prescription of ≥4 regular medications on or within 3 months before baseline; (2) With diagnosis of DM, CVD or CKD on or before baseline; (3) With record of aspirin prescription on the baseline; (4) Patients with incomplete data for the used covariates at the baseline<br><br>Baseline is defined as the first date of prescription adjustment, between 1 January and 31 December 2013.
Treatment strategy	Intensive treatment<br><br>***versus***<br>Standard treatment<br>Seen monthly for the first three months and every 3 months thereafter<br>Intensive treatment group:<br>Participants in the intensive-treatment group were adjusted monthly based on monthly basis to target a SBP of less than 120 mmHg.<br><br>Standard-treatment group:<br>were adjusted to target a SBP of 135-139 mmHg. and the dose was reduced if systolic blood pressure was less than 130 mm Hg on a single visit or less than 135 mm Hg on two consecutive visits.	1. SBP 130-140mmHg and DBP 80-90mmHg<br>2. SBP below 130mmHg and DBP below 80 mmHg

	Dose adjustment was based on a mean of three blood-pressure measurements were made with the use of an automated measurement system.	
Treatment assignment	Eligible participants were assigned to a systolic blood-pressure target of either less than 140 mm Hg (the standard-treatment group) or less than 120 mm Hg (the intensive-treatment group). Randomization was stratified according to clinical site. Participants and study personnel were aware of the study-group assignments, but outcome adjudicators were not.	Participants will be classified into different treatment strategies according to the blood pressure and prescription records during the first 12 months (grace period) after baseline.<br><br>Patients with two consecutive records of BP <130/80 mmHg without de-escalation or BP 130-140/ 80-90 mmHg with escalation of drugs treatment were considered to be following intensive treatment target.<br><br>The rest of the patients with two consecutive records of BP less than 140/90mmHg considered that they continued being treated according to the current treatment guideline below 140/90mmHg.<br><br>Those patients who do not satisfy the aforementioned criteria or patients without follow-up after baseline will be artificially censored.<br><br>Potential immortal time bias will be accounted for using the cloning and censoring approach. Inverse probability weights were used to adjust for post-assignment confounders.<br><br>Randomization of treatment assignment will be emulated by regression adjustment of baseline confounders in the pooled logistic model.
Outcomes	Primary outcome:<br>The composite outcome of myocardial infarction, acute coronary syndrome not resulting in myocardial infarction, stroke, acute decompensated heart failure, or death from cardiovascular causes.<br><br>Secondary outcome:<br>1. Individual components of the primary composite outcome<br>2. Death from any cause<br>3. The composite of the primary outcome or death from any cause.<br>4. Renal outcomes	Primary outcome:<br>The risk of CVD and all-cause mortality<br><br>Secondary outcomes<br>The risk of each of the following events:<br>1.Total CVD<br>2.Coronary heart disease (CHD)<br>3.Stroke<br>4.Heart failure<br>5.All-cause mortality<br>6.End-stage renal disease (ESRD)<br>7.Seven serious adverse events:<br>i. Hypotension<br>ii. Syncope<br>iii. Bradycardia<br>iv. Electrolyte abnormality

		v. Fall(s)<br>vi. Acute kidney injury or acute renal failure<br>vii. Dizziness
Follow-up	Follow-up time will be censored at the last date of event ascertainment	All patients were followed from the baseline until the outcome events, deviated from the assigned strategy (except for the contraindication), death or the end of the study (31 Dec 2018), whichever occurred first.
Causal contrasts of interest	Intention-to-treat effect	Observational analogue of the per-protocol effect
Statistical analysis	Intention-to-treat analysis	Per-protocol analysis: patients are censored when they deviate from their respective treatment strategies. Pre-assignment and post-assignment confounders will be adjusted for.

Supplementary Table 2 Disease definition

Events		ICPC-2	ICD-9CM
Subjects	Hypertension	K86; K87	401.x-405.x
Outcomes of interest	Chronic heart disease	K74, K82, K76	411.x-414.x
	Heart failure	K77	428.x
	Stroke	K89; K90; K91	430.x-438.x
	Adverse events		
	Hypotension	K88	458.0, 458.1, 458.29, 458.8, 458.9
	Syncope	A06, N17	780.2
	Bradycardia	-	427.81 427.89
	Electrolyte abnormality	A91	276.9
	Falls	-	E880-E888
	Acute kidney disease	-	584.1,584.2,584.3 ,584.4,584.5,584.6,584.7, 584.8 ,584.9
	Dizziness	N17	780.4

Supplementary Table 3 Construction of inverse probability weights.

**Time point**	**Fitting the weighting model**<br>(Predicting the probability of the adherence to the assigned strategy)	**Construction of stabilized IP weights**<br>**Traditional follow-up arm**	 **Intensive follow-up arm**
Within grace period	**Denominator:**<br>$logit(\Pr[A(\text{intensive\_}assign)_k = 1 \mid L_0, L_k, Y_{k-1} = 0]) = \mu_0 + \mu_1^T L_0 + \mu_2^T L_k$<br>**Numerator:**<br>$logit(\Pr[A(\text{intensive\_}assign)_k = 1 \mid L_0, Y_{k-1} = 0]) = \varphi_0 + \varphi_1^T L_0$	1	1
End of grace period		(1-Pr_n_(intensive_assign))/<br>(1-Pr_d_(intensive_assign))	Pr_n_(intensive_assign)<br>/<br>Pr_d_(intensive_assign<br>)
After grace period	**Denominator:**<br>*Traditional treatment arm* $logit$<br>$(\Pr[A(\text{traditional\_}current)_k = 1 \mid A(\text{traditional\_}current)_{k-1} = 1, L_0, L_k, Y_{k-1} = 0])$<br>$= \theta_0 + \theta_1^T L_0 + \theta_2^T L_k$<br>*Intensive treatment arm*<br>$logit(\Pr[A(\text{intensive\_}current)_k = 1 \mid A(\text{intensive\_}current)_{k-1} = 1, L_0, L_k, Y_{k-1} = 0])$<br>$= \delta_0 + \delta_1^T L_0 + \delta_2^T L_k$<br>**Numerator:**<br>*Traditional treatment arm* $logit$<br>$(Pr[A(\text{traditional\_}current)_k = 1 \mid A(\text{traditional\_}current)_{k-1} = 1, L_0, Y_{k-1} = 0]) =$<br>$\beta_0 +$<br>$\beta_1^T L_0$<br>*Intensive treatment arm*<br>$logit(\Pr[A(\text{intensive\_}current)_k = 1 \mid A(\text{intensive\_}current)_{k-1} = 1, L_0, Y_{k-1} = 0]) =$<br>$\alpha_0 + \alpha_1^T L_0$	Pr_n_(traditional _current)/<br>Pr_d_(traditional_current)	Pr_n_(intensive_current)/<br>Pr_d_(intensive_current)

Supplementary Table 4 Distribution of estimated weights

Percentile	SBP <130mmHg and DBP <80 (Before truncation)	SBP <130mmHg and DBP <80 (After truncation)
Min	0.3794	0.9746
1%	0.9917	0.9917
5%	0.9974	0.9974
10%	0.9991	0.9991
25%	1	1
50%	1	1
75%	1	1
90%	1.0010	1.0010
95%	1.0021	1.0021
99%	1.0096	1.0097
Mean	1.0001	1.0001

Notes: The cumulative weights for each patient at each time points were truncated at the 0.5^{th} and 99.5^{th} percentile to avoid the influence of outliers in the estimated weights when estimating the results. The weight distribution presented above was calculated after truncation.

Supplementary Table 5. Estimated hazard ratios (and 95% confidence interval) of hypertension related complications, all-cause mortality and serious adverse events between intensive treatment target group and traditional treatment target group in female and male subgroup

Outcome	Adjusted hazard ratio (95% CI)	P value for interaction
**Major CVD**		
Female	0.89 (0.80-0.99)	
Male	0.82 (0.74-0.90)	0.21
**CHD**		
Female	0.92 (0.77-1.09)	
Male	0.74 (0.64-0.86)	0.06
**Heart Failure**		
Female	0.82 (0.63-1.07)	
Male	0.86 (0.66-1.10)	0.83
**Stroke**		
Female	0.89 (0.77-1.04)	
Male	0.89 (0.77-1.02)	0.98
**ESRD**		
Female	0.72 (0.53-0.99)	
Male	0.85 (0.65-1.11)	0.45
**Mortality**		
Female	0.91 (0.78-1.06)	
Male	0.86 (0.75-0.98)	0.55
Serious adverse event		
**Composite of seven serious adverse event**		
Female	0.98 (0.91-1.05)	
Male	0.99 (0.91-1.08)	0.70
**Hypotension**		
Female	0.97 (0.62-1.53)	
Male	1.00 (0.73-1.39)	0.13
**Syncope**		
Female	0.86 (0.70-1.07)	
Male	1.10 (0.92-1.31)	0.73
**Bradycardia**		
Female	1.06 (0.55-2.02)	
Male	0.67 (0.23-1.95)	0.95
**Electrolyte abnormality**		
Female	1.09 (0.13-9.02)	
Male	1.90 (0.23-16.05)	0.72
**Falls**		
Female	0.99 (0.89-1.09)	
Male	1.01 (0.89-1.16)	0.35
**Acute kidney disease**		
Female	0.80 (0.54-1.19)	
Male	0.87 (0.65-1.17)	0.80
**Dizziness**		
Female	1.04 (0.93-1.16)	
Male	0.96 (0.83-1.10)	0.17

Note: Major CVD: composite outcomes of heart failure, chronic heart disease and stroke; CHD: chronic heart disease; ESRD: end-stage renal disease; Analyses adjusted for age, smoking status, fasting glucose, high-density lipoprotein cholesterol, low-density lipoprotein cholesterol, triglyceride, total cholesterol, eGFR, Charlson Comorbidities Index, usage of ACEI/ARB, β-blocker, calcium channel blockers, diuretic; history of adverse events, obesity status, specialist outpatient clinics attendance, general outpatient clinics attendance, accident and emergency attendance and hospitalization (within 1 year before baseline). Statistical significance was defined as a two-tailed *p*-value.

Supplementary Table 6. Estimated hazard ratios (and 95% confidence interval) of hypertension related complications, all-cause mortality and serious adverse events between intensive treatment target group and traditional treatment target group in non-smoker and smoker subgroup

Outcome	Adjusted hazard ratio (95% CI)	P value for interaction
**Major CVD**		
Non-smoker	0.86 (0.79-0.92)	
Smoker	0.79 (0.63-0.99)	0.51
**CHD**		
Non-smoker	0.81 (0.72-0.91)	
Smoker	0.78 (0.56-1.07)	0.79
**Heart Failure**		
Non-smoker	0.83 (0.68-1.01)	
Smoker	0.91 (0.51-1.62)	0.77
**Stroke**		
Non-smoker	0.89 (0.80-0.99)	
Smoker	0.87 (0.63-1.22)	0.91
**ESRD**		
Non-smoker	0.75 (0.60-0.94)	
Smoker	1.13 (0.64-1.99)	0.19
**Mortality**		
Non-smoker	0.91 (0.82-1.01)	
Smoker	0.66 (0.48-0.90)	0.05
Serious adverse event		
**Composite of seven serious adverse event**		
Non-smoker	0.79 (0.72-0.88)	
Smoker	0.93 (0.65-1.33)	0.82
**Hypotension**		
Non-smoker	0.71 (0.44-1.16)	
Smoker	0.58 (0.13-2.57)	0.80
**Syncope**		
Non-smoker	0.85 (0.67-1.08)	
Smoker	0.64 (0.27-1.50)	0.53
**Bradycardia**		
Non-smoker	0.36 (0.11-1.21)	
Smoker	2.15 (0.39-11.88)	0.09
**Electrolyte abnormality**		
Non-smoker	NA	
Smoker	1.39 (0.32-6.16)	NA
**Falls**		
Non-smoker	0.78 (0.68-0.91)	
Smoker	1.06 (0.61-1.85)	0.30
**Acute kidney disease**		
Non-smoker	0.44 (0.27-0.74)	
Smoker	0.93 (0.32-2.69)	0.21
**Dizziness**		
Non-smoker	0.81 (0.69-0.96)	
Smoker	0.96 (0.53-1.73)	0.60

Note: Major CVD: composite outcomes of heart failure, chronic heart disease and stroke; CHD: chronic heart disease; ESRD: end-stage renal disease; NA: convergency not achieved; Analyses adjusted for sex, age, fasting glucose, high-density lipoprotein cholesterol, low-density lipoprotein cholesterol, triglyceride, total cholesterol, eGFR, Charlson Comorbidities Index, usage of ACEI/ARB, β-blocker, calcium channel blockers, diuretic; history of adverse events, obesity status, specialist outpatient clinics attendance, general outpatient clinics attendance, accident and emergency attendance and hospitalization (within 1 year before baseline). Statistical significance was defined as a two-tailed *p*-value.

Supplementary Table 7. Estimated hazard ratios (and 95% confidence interval) of hypertension related complications, all-cause mortality and serious adverse events between intensive treatment target group and traditional treatment target group in CCI<4 and CCI≥4 subgroup

Outcome	Adjusted hazard ratio (95% CI)	P value for interaction
**Major CVD**		
CCI<4	0.84 (0.76-0.91)	
CCI≥4	0.89 (0.78-1.00)	0.43
**CHD**		
CCI<4	0.78 (0.68-0.89)	
CCI≥4	0.90 (0.73-1.10)	0.27
**Heart Failure**		
CCI<4	0.78 (0.59-1.04)	
CCI≥4	0.90 (0.70-1.14)	0.46
**Stroke**		
CCI<4	0.91 (0.80-1.03)	
CCI≥4	0.87 (0.73-1.03)	0.68
**ESRD**		
CCI<4	0.77 (0.59-1.01)	
CCI≥4	0.82 (0.60-1.13)	0.76
**Mortality**		
CCI<4	0.88 (0.76-1.00)	
CCI≥4	0.90 (0.78-1.03)	0.82
Serious adverse event		
**Composite of seven serious adverse event**		
CCI<4	0.80 (0.71-0.90)	
CCI≥4	0.84 (0.72-0.99)	0.02
**Hypotension**		
CCI<4	0.82 (0.46-1.47)	
CCI≥4	0.57 (0.27-1.19)	0.73
**Syncope**		
CCI<4	0.89 (0.69-1.16)	
CCI≥4	0.70 (0.46-1.09)	0.49
**Bradycardia**		
CCI<4	0.75 (0.25-2.23)	
CCI≥4	0.25 (0.03-1.94)	0.61
**Electrolyte abnormality**		
CCI<4	NA	
CCI≥4	NA	NA
**Falls**		
CCI<4	0.84 (0.71-1.00)	
CCI≥4	0.73 (0.58-0.94)	0.08
**Acute kidney disease**		
CCI<4	0.54 (0.32-0.93)	
CCI≥4	0.41 (0.18-0.94)	0.27
**Dizziness**		
CCI<4	0.70 (0.58-0.85)	
CCI≥4	1.17 (0.90-1.53)	0.17

Note: Major CVD: composite outcomes of heart failure, chronic heart disease and stroke; CHD: chronic heart disease; ESRD: end-stage renal disease; NA: convergency not achieved; Analyses adjusted for sex, age, smoking status, fasting glucose, high-density lipoprotein cholesterol, low-density lipoprotein cholesterol, triglyceride, total cholesterol, eGFR, usage of ACEI/ARB, β-blocker, calcium channel blockers, diuretic; history of adverse events, obesity status, specialist outpatient clinics attendance, general outpatient clinics attendance, accident and emergency attendance and hospitalization (within 1 year before baseline). Statistical significance was defined as a two-tailed *p*-value.

Supplementary Table 8. Estimated hazard ratios (and 95% confidence interval) of hypertension related complications, all-cause mortality and serious adverse events between intensive treatment target group and traditional treatment target group in age<65, age 65-80 and age≥80 subgroup

Outcome	Adjusted hazard ratio (95% CI)	P value for interaction
**Major CVD**		
Age<65	0.83 (0.76-0.92)	
Age 65-80	0.92 (0.82-1.03)	0.18
Age≥80	0.79 (0.61-1.03)	0.72
**CHD**		
Age<65	0.79 (0.61-1.03)	
Age 65-80	0.78 (0.68-0.90)	0.25
Age≥80	0.89 (0.74-1.07)	0.71
**Heart Failure**		
Age<65	0.89 (0.74-1.07)	
Age 65-80	0.86 (0.52-1.41)	0.33
Age≥80	0.75 (0.53-1.05)	0.58
**Stroke**		
Age<65	0.75 (0.53-1.05)	
Age 65-80	0.92 (0.72-1.19)	0.94
Age≥80	0.88 (0.57-1.35)	0.18
**ESRD**		
Age<65	0.88 (0.57-1.35)	
Age 65-80	0.93 (0.80-1.07)	0.59
Age≥80	0.92 (0.79-1.08)	0.45
**Mortality**		
Age<65	0.92 (0.79-1.08)	
Age 65-80	0.71 (0.48-1.03)	0.47
Age≥80	0.74 (0.54-1.01)	0.40
Serious adverse event		
**Composite of seven serious adverse event**		
Age<65	0.84 (0.74-0.95)	
Age 65-80	0.74 (0.63-0.88)	0.25
Age≥80	0.99 (0.66-1.49)	0.19
**Hypotension**		
Age<65	0.99 (0.66-1.49)	
Age 65-80	0.99 (0.68-1.44)	0.75
Age≥80	1.09 (0.91-1.30)	0.99
**Syncope**		
Age<65	1.09 (0.91-1.30)	
Age 65-80	0.89 (0.71-1.11)	0.56
Age≥80	0.81 (0.27-2.39)	0.67
**Bradycardia**		
Age<65	0.81 (0.27-2.39)	
Age 65-80	1.00 (0.51-1.96)	0.12
Age≥80	1.12 (0.14-8.81)	0.99
**Electrolyte abnormality**		
Age<65	1.12 (0.14-8.81)	
Age 65-80	3.05 (0.29-31.79)	0.53
Age≥80	1.02 (0.91-1.14)	0.78
**Falls**		
Age<65	1.02 (0.91-1.14)	
Age 65-80	1.06 (0.94-1.21)	0.96

Age≥80	0.73 (0.50-1.06)	0.89
**Acute kidney disease**		
Age<65	0.73 (0.50-1.06)	
Age 65-80	0.97 (0.70-1.35)	0.27
Age≥80	0.99 (0.88-1.12)	0.53
**Dizziness**		
Age<65	0.99 (0.88-1.12)	
Age 65-80	1.04 (0.90-1.20)	0.09
Age≥80	1.17 (0.85-1.60)	<0.001

Note: Major CVD: composite outcomes of heart failure, chronic heart disease and stroke; CHD: chronic heart disease; ESRD: end-stage renal disease; Analyses adjusted for sex, smoking status, fasting glucose, high-density lipoprotein cholesterol, low-density lipoprotein cholesterol, triglyceride, total cholesterol, eGFR, Charlson Comorbidities Index, usage of ACEI/ARB, β-blocker, calcium channel blockers, diuretic; history of adverse events, obesity status, specialist outpatient clinics attendance, general outpatient clinics attendance, accident and emergency attendance and hospitalization (within 1 year before baseline). Statistical significance was defined as a two-tailed *p*-value.

Supplementary Table 9. Estimated hazard ratios (and 95% confidence interval) of hypertension related complications, all-cause mortality and serious adverse events between intensive treatment target group and traditional treatment target group in low, middle and high CVD risk subgroup

Outcome	Adjusted hazard ratio (95% CI)	P value for interaction
**Major CVD**		
CVD risk<10	0.87 (0.79-0.96)	
CVD risk 10-20	0.80 (0.69-0.93)	0.86
CVD risk≥20	0.86 (0.75-0.98)	0.93
**CHD**		
CVD risk<10	0.86 (0.75-0.98)	
CVD risk 10-20	0.87 (0.75-1.02)	0.14
CVD risk≥20	0.71 (0.57-0.88)	0.20
**Heart Failure**		
CVD risk<10	0.71 (0.57-0.88)	
CVD risk 10-20	0.80 (0.64-0.99)	0.38
CVD risk≥20	0.87 (0.67-1.12)	0.90
**Stroke**		
CVD risk<10	0.87 (0.67-1.12)	
CVD risk 10-20	0.58 (0.37-0.93)	0.36
CVD risk≥20	0.99 (0.72-1.36)	0.70
**ESRD**		
CVD risk<10	0.99 (0.72-1.36)	
CVD risk 10-20	0.86 (0.74-0.99)	0.36
CVD risk≥20	0.98 (0.80-1.22)	0.30
**Mortality**		
CVD risk<10	0.98 (0.80-1.22)	
CVD risk 10-20	0.87 (0.72-1.06)	0.43
CVD risk≥20	0.74 (0.55-0.99)	0.27
Serious adverse event		
**Composite of seven serious adverse event**		
CVD risk<10	0.98 (0.91-1.05)	
CVD risk 10-20	1.10 (0.97-1.25)	0.62
CVD risk≥20	0.93 (0.60-1.44)	0.72
**Hypotension**		
CVD risk<10	0.93 (0.60-1.44)	
CVD risk 10-20	1.17 (0.72-1.89)	0.73
CVD risk≥20	0.93 (0.77-1.13)	0.49
**Syncope**		
CVD risk<10	0.93 (0.77-1.13)	
CVD risk 10-20	1.09 (0.83-1.43)	0.49
CVD risk≥20	1.02 (0.54-1.94)	0.51
**Bradycardia**		
CVD risk<10	1.02 (0.54-1.94)	
CVD risk 10-20	0.66 (0.08-5.39)	0.61
CVD risk≥20	0.79 (0.10-6.03)	0.82
**Electrolyte abnormality**		
CVD risk<10	0.79 (0.10-6.03)	
CVD risk 10-20	0.79 (0.10-6.03)	0.69
CVD risk≥20	0.98 (0.89-1.08)	0.22
**Falls**		
CVD risk<10	0.98 (0.89-1.08)	
CVD risk 10-20	1.20 (0.99-1.46)	0.09

CVD risk≥20	0.77 (0.54-1.11)	0.38
**Acute kidney disease**		
CVD risk<10	0.77 (0.54-1.11)	
CVD risk 10-20	0.92 (0.57-1.51)	0.27
CVD risk≥20	1.03 (0.93-1.15)	0.36
**Dizziness**		
CVD risk<10	1.03 (0.93-1.15)	
CVD risk 10-20	1.01 (0.81-1.26)	0.17
CVD risk≥20	0.91 (0.74-1.12)	0.14

Note: Major CVD: composite outcomes of heart failure, chronic heart disease and stroke; CHD: chronic heart disease; ESRD: end-stage renal disease; Analyses adjusted for sex, age, smoking status, fasting glucose, high-density lipoprotein cholesterol, low-density lipoprotein cholesterol, triglyceride, total cholesterol, eGFR, Charlson Comorbidities Index, usage of ACEI/ARB, β-blocker, calcium channel blockers, diuretic; history of adverse events, obesity status, specialist outpatient clinics attendance, general outpatient clinics attendance, accident and emergency attendance and hospitalization (within 1 year before baseline). Statistical significance was defined as a two-tailed *p*-value.

Supplementary Table 10. Estimated hazard ratios (and 95% confidence interval) of hypertension related complications, all-cause mortality and serious adverse events between intensive treatment target group and traditional treatment target group in non-obesity and obesity subgroup

Outcome	Adjusted hazard ratio (95% CI)	P value for interaction
**Major CVD**		
Non-obesity	0.85 (0.78-0.92)	
Obesity	0.85 (0.70-1.04)	0.36
**CHD**		
Non-obesity	0.79 (0.70-0.89)	
Obesity	0.96 (0.71-1.29)	0.37
**Heart Failure**		
Non-obesity	0.82 (0.67-1.01)	
Obesity	0.93 (0.62-1.40)	0.95
**Stroke**		
Non-obesity	0.91 (0.81-1.01)	
Obesity	0.75 (0.55-1.04)	0.46
**ESRD**		
Non-obesity	0.84 (0.67-1.05)	
Obesity	0.55 (0.31-0.97)	0.20
**Mortality**		
Non-obesity	0.89 (0.80-0.98)	
Obesity	0.82 (0.59-1.13)	0.69
Serious adverse event		
**Composite of seven serious adverse event**		
Non-obesity	1.00 (0.94-1.06)	
Obesity	0.87 (0.74-1.03)	0.82
**Hypotension**		
Non-obesity	1.08 (0.82-1.42)	
Obesity	0.26 (0.06-1.10)	0.80
**Syncope**		
Non-obesity	1.02 (0.89-1.18)	
Obesity	0.72 (0.44-1.19)	0.53
**Bradycardia**		
Non-obesity	0.90 (0.50-1.62)	
Obesity	1.15 (0.25-5.36)	0.09
**Electrolyte abnormality**		
Non-obesity	NA	
Obesity	1.89 (0.39-9.15)	NA
**Falls**		
Non-obesity	1.01 (0.92-1.10)	
Obesity	0.93 (0.72-1.20)	0.30
**Acute kidney disease**		
Non-obesity	0.91 (0.71-1.17)	
Obesity	0.54 (0.27-1.06)	0.21
**Dizziness**		
Non-obesity	1.00 (0.91-1.10)	
Obesity	1.03 (0.80-1.32)	0.60

Note: Major CVD: composite outcomes of heart failure, chronic heart disease and stroke; CHD: chronic heart disease; ESRD: end-stage renal disease; NA: convergency not achieved; Analyses adjusted for sex, age, smoking status, fasting glucose, high-density lipoprotein cholesterol, low-density lipoprotein cholesterol, triglyceride, total cholesterol, eGFR, Charlson Comorbidities Index, usage of ACEI/ARB, β-blocker, calcium channel blockers, diuretic; history of adverse events, specialist outpatient clinics attendance, general outpatient clinics attendance, accident and emergency attendance and hospitalization (within 1 year before baseline). Statistical significance was defined as a two-tailed *p*-value.

Supplementary Table 11 Estimated hazard ratios (and 95% confidence interval) of hypertension related complications, all-cause mortality and serious adverse events between intensive treatment target group and traditional treatment target group (Intention to treat)

Outcome	Adjusted hazard ratio (95% CI)	P value
Major CVD	0.85 (0.79-0.91)	<0.001
CHD	0.81 (0.72-0.90)	<0.001
Heart Failure	0.83 (0.69-1.00)	0.06
Stroke	0.89 (0.80-0.98)	0.02
ESRD	0.79 (0.64-0.97)	0.03
Mortality	0.88 (0.80-0.97)	0.01
Serious adverse event		
Composite of seven serious adverse event	0.99 (0.93-1.04)	0.60
Hypotension	0.99 (0.76-1.29)	0.92
Syncope	0.99 (0.86-1.14)	0.87
Bradycardia	0.91 (0.52-1.58)	0.73
Electrolyte abnormality	1.36 (0.31-6.01)	0.68
Falls	0.99 (0.92-1.08)	0.90
Acute kidney disease	1.36 (0.31-6.01)	0.68
Dizziness	1.00 (0.92-1.09)	0.98

Note: Major CVD: composite outcomes of heart failure, chronic heart disease and stroke; CHD: chronic heart disease; ESRD: end-stage renal disease; Analyses adjusted for sex, age, smoking status, fasting glucose, high-density lipoprotein cholesterol, low-density lipoprotein cholesterol, triglyceride, total cholesterol, eGFR, Charlson Comorbidities Index, usage of ACEI/ARB, β-blocker, calcium channel blockers, diuretic; history of adverse events, obesity status, specialist outpatient clinics attendance, general outpatient clinics attendance, accident and emergency attendance and hospitalization (within 1 year before baseline). Statistical significance was defined as a two-tailed *p*-value.

Supplementary Table 12 Estimated hazard ratios (and 95% confidence interval) of hypertension related complications, all-cause mortality and serious adverse events between intensive treatment target group and traditional treatment target group (Lost to follow-up)

Outcome	Adjusted hazard ratio (95% CI)	P value
Major CVD	0.85 (0.79-0.91)	<0.001
CHD	0.81 (0.72-0.90)	<0.001
Heart Failure	0.83 (0.69-1.00)	0.06
Stroke	0.86 (0.77-0.96)	0.01
ESRD	0.79 (0.64-0.97)	0.03
Mortality	0.89 (0.80-0.98)	0.02
Serious adverse event		
Composite of seven serious adverse event	0.99 (0.94-1.05)	0.76
Hypotension	0.99 (0.76-1.29)	0.94
Syncope	0.99 (0.87-1.14)	0.94
Bradycardia	0.92 (0.52-1.62)	0.77
Electrolyte abnormality	1.42 (0.33-6.19)	0.64
Falls	1.00 (0.92-1.09)	0.97
Acute kidney disease	0.84 (0.67-1.07)	0.16
Dizziness	1.01 (0.92-1.10)	0.85

Note: Major CVD: composite outcomes of heart failure, chronic heart disease and stroke; CHD: chronic heart disease; ESRD: end-stage renal disease; Analyses adjusted for sex, age, smoking status, fasting glucose, high-density lipoprotein cholesterol, low-density lipoprotein cholesterol, triglyceride, total cholesterol, eGFR, Charlson Comorbidities Index, usage of ACEI/ARB, β-blocker, calcium channel blockers, diuretic; history of adverse events, obesity status, specialist outpatient clinics attendance, general outpatient clinics attendance, accident and emergency attendance and hospitalization (within 1 year before baseline). Statistical significance was defined as a two-tailed *p*-value.

Supplementary Table 13 Estimated hazard ratios (and 95% confidence interval) of hypertension related complications, all-cause mortality and serious adverse events between intensive treatment target group and traditional treatment target group (using 6-month as grace period)

Outcome	Adjusted hazard ratio (95% CI)	P value
Major CVD	0.80 (0.73-0.88)	<0.001
CHD	0.71 (0.62-0.82)	<0.001
Heart Failure	0.88 (0.70-1.11)	0.27
Stroke	0.83 (0.73-0.95)	0.01
ESRD	0.84 (0.62-1.12)	0.23
Mortality	0.84 (0.73-0.98)	0.03
Serious adverse event		
Composite of seven serious adverse event	0.93 (0.87-1.00)	0.07
Hypotension	0.88 (0.62-1.27)	0.50
Syncope	1.02 (0.85-1.22)	0.84
Bradycardia	0.44 (0.15-1.26)	0.13
Electrolyte abnormality	1.80 (0.22-14.94)	0.59
Falls	0.90 (0.81-1.01)	0.07
Acute kidney disease	0.79 (0.56-1.10)	0.16
Dizziness	0.98 (0.87-1.10)	0.74

Note: Major CVD: composite outcomes of heart failure, chronic heart disease and stroke; CHD: chronic heart disease; ESRD: end-stage renal disease; Analyses adjusted for sex, age, smoking status, fasting glucose, high-density lipoprotein cholesterol, low-density lipoprotein cholesterol, triglyceride, total cholesterol, eGFR, Charlson Comorbidities Index, usage of ACEI/ARB, β-blocker, calcium channel blockers, diuretic; history of adverse events, obesity status, specialist outpatient clinics attendance, general outpatient clinics attendance, accident and emergency attendance and hospitalization (within 1 year before baseline). Statistical significance was defined as a two-tailed *p*-value.

Supplementary Table 14 Estimated hazard ratios (and 95% confidence interval) of hypertension related complications, all-cause mortality and serious adverse events between intensive treatment target group and traditional treatment target group (using single record for defining treatment strategy)

Outcome	Adjusted hazard ratio (95% CI)	P value
Major CVD	0.83 (0.77-0.90)	<0.001
CHD	0.85 (0.76-0.95)	<0.001
Heart Failure	1.10 (0.90-1.35)	0.36
Stroke	0.81 (0.72-0.90)	<0.001
ESRD	0.81 (0.62-1.05)	0.11
Mortality	0.88 (0.79-0.99)	0.03
Serious adverse event		
Composite of seven serious adverse event	1.01 (0.96-1.07)	0.72
Hypotension	1.05 (0.82-1.34)	0.71
Syncope	0.98 (0.86-1.11)	0.75
Bradycardia	0.58 (0.33-1.01)	0.05
Electrolyte abnormality	0.84 (0.20-3.56)	0.82
Falls	0.98 (0.91-1.06)	0.62
Acute kidney disease	0.84 (0.20-3.56)	0.82
Dizziness	1.02 (0.94-1.11)	0.58

Note: Major CVD: composite outcomes of heart failure, chronic heart disease and stroke; CHD: chronic heart disease; ESRD: end-stage renal disease; Analyses adjusted for sex, age, smoking status, fasting glucose, high-density lipoprotein cholesterol, low-density lipoprotein cholesterol, triglyceride, total cholesterol, eGFR, Charlson Comorbidities Index, usage of ACEI/ARB, β-blocker, calcium channel blockers, diuretic; history of adverse events, obesity status, specialist outpatient clinics attendance, general outpatient clinics attendance, accident and emergency attendance and hospitalization (within 1 year before baseline). Statistical significance was defined as a two-tailed *p*-value.

Supplementary Table 15 Estimated hazard ratios (and 95% confidence interval) of hypertension related complications, all-cause mortality and serious adverse events between intensive treatment target group and traditional treatment target group (using 3 consecutive records for defining treatment strategy)

Outcome	Adjusted hazard ratio (95% CI)	P value
Major CVD	0.78 (0.71-0.87)	<0.001
CHD	0.69 (0.59-0.81)	<0.001
Heart Failure	0.89 (0.69-1.15)	0.38
Stroke	0.86 (0.74-0.99)	0.04
ESRD	0.73 (0.54-0.98)	0.03
Mortality	0.79 (0.68-0.92)	0.002
Serious adverse event		
Composite of seven serious adverse event	0.96 (0.89-1.03)	0.26
Hypotension	0.58 (0.37-0.92)	0.02
Syncope	0.89 (0.73-1.08)	0.24
Bradycardia	0.58 (0.19-1.74)	0.33
Electrolyte abnormality	1.90 (0.23-16.04)	0.56
Falls	0.95 (0.85-1.07)	0.40
Acute kidney disease	0.67 (0.45-1.00)	0.05
Dizziness	1.05 (0.93-1.18)	0.45

Note: Major CVD: composite outcomes of heart failure, chronic heart disease and stroke; CHD: chronic heart disease; ESRD: end-stage renal disease; Analyses adjusted for sex, age, smoking status, fasting glucose, high-density lipoprotein cholesterol, low-density lipoprotein cholesterol, triglyceride, total cholesterol, eGFR, Charlson Comorbidities Index, usage of ACEI/ARB, β-blocker, calcium channel blockers, diuretic; history of adverse events, obesity status, specialist outpatient clinics attendance, general outpatient clinics attendance, accident and emergency attendance and hospitalization (within 1 year before baseline). Statistical significance was defined as a two-tailed *p*-value.

Supplementary Table 16 Estimated hazard ratios (and 95% confidence interval) of adverse events between intensive treatment target group and traditional treatment target group (incidence of adverse events from both inpatient and outpatient settings)

Outcome	Adjusted hazard ratio (95% CI)	P value
Serious adverse event		
Composite of seven serious adverse event	0.99 (0.97-1.02)	0.55
Hypotension	0.96 (0.70-1.33)	0.82
Syncope	1.06 (1.01-1.11)	0.03
Bradycardia	0.96 (0.54-1.68)	0.88
Electrolyte abnormality	0.97 (0.94-1.01)	0.11
Falls	0.98 (0.91-1.07)	0.70
Acute kidney disease	0.85 (0.67-1.08)	0.18
Dizziness	1.05 (1.00-1.10)	0.05

Note: Major CVD: composite outcomes of heart failure, chronic heart disease and stroke; CHD: chronic heart disease; ESRD: end-stage renal disease; Analyses adjusted for sex, age, smoking status, fasting glucose, high-density lipoprotein cholesterol, low-density lipoprotein cholesterol, triglyceride, total cholesterol, eGFR, Charlson Comorbidities Index, usage of ACEI/ARB, β-blocker, calcium channel blockers, diuretic; history of adverse events, obesity status, specialist outpatient clinics attendance, general outpatient clinics attendance, accident and emergency attendance and hospitalization (within 1 year before baseline). Statistical significance was defined as a two-tailed *p*-value.

Supplementary Table 17 Estimated hazard ratios (and 95% confidence interval) of safety outcome between intensive treatment target group and traditional treatment target group (using cancer as a negative control outcome)

Outcome	Adjusted hazard ratio (95% CI)	P value
Cancer	1.03 (0.97-1.10)	0.23

Note: Analyses adjusted for sex, age, smoking status, fasting glucose, high-density lipoprotein cholesterol, low-density lipoprotein cholesterol, triglyceride, total cholesterol, eGFR, Charlson Comorbidities Index, usage of ACEI/ARB, β-blocker, calcium channel blockers, diuretic; history of adverse events, obesity status, specialist outpatient clinics attendance, general outpatient clinics attendance, accident and emergency attendance and hospitalization (within 1 year before baseline). Statistical significance was defined as a two-tailed *p*-value.

Supplementary Table 18 Estimated hazard ratios (and 95% confidence interval) of hypertension related complications, all-cause mortality and serious adverse events between intensive treatment target group and traditional treatment target group (Including baseline BP readings in the weighting model)

Outcome	Adjusted hazard ratio (95% CI)	P value
Major CVD	0.86 (0.80-0.92)	<0.001
CHD	0.81 (0.72-0.90)	<0.001
Heart Failure	0.90 (0.81-1.00)	0.06
Stroke	0.85 (0.70-1.02)	0.09
ESRD	0.81 (0.66-0.99)	0.04
Mortality	0.90 (0.82-0.99)	0.04
Serious adverse event		
Composite of seven serious adverse event	0.99 (0.93-1.04)	0.66
Hypotension	1.01 (0.77-1.32)	0.96
Syncope	0.98 (0.85-1.13)	0.80
Bradycardia	0.92 (0.53-1.62)	0.78
Electrolyte abnormality	1.59 (0.36-7.05)	0.54
Falls	1.01 (0.93-1.10)	0.81
Acute kidney disease	0.86 (0.68-1.10)	0.23
Dizziness	1.00 (0.91-1.09)	0.96

Note: Major CVD: composite outcomes of heart failure, chronic heart disease and stroke; CHD: chronic heart disease; ESRD: end-stage renal disease; Analyses adjusted for sex, age, smoking status, fasting glucose, high-density lipoprotein cholesterol, low-density lipoprotein cholesterol, triglyceride, total cholesterol, eGFR, Charlson Comorbidities Index, usage of ACEI/ARB, β-blocker, calcium channel blockers, diuretic; history of adverse events, obesity status, specialist outpatient clinics attendance, general outpatient clinics attendance, accident and emergency attendance and hospitalization (within 1 year before baseline). Statistical significance was defined as a two-tailed *p*-value.

Supplementary Table 19 Estimated hazard ratios (and 95% confidence interval) of hypertension related complications, all-cause mortality and serious adverse events between intensive treatment target group and traditional treatment target group (adjusting for competing risk)

Outcome	Adjusted hazard ratio (95% CI)	P value
Major CVD	0.90 (0.83-0.98)	0.02
CHD	0.85 (0.74-0.97)	0.02
Heart Failure	0.98 (0.87-1.10)	0.71
Stroke	0.98 (0.78-1.22)	0.83
ESRD	0.90 (0.70-1.15)	0.38
Serious adverse event		
Composite of seven serious adverse event	0.99 (0.94-1.05)	0.77
Hypotension	1.00 (0.77-1.31)	0.99
Syncope	1.00 (0.87-1.15)	0.99
Bradycardia	0.94 (0.53-1.64)	0.82
Electrolyte abnormality	1.43 (0.34-6.08)	0.63
Falls	1.01 (0.93-1.09)	0.89
Acute kidney disease	0.86 (0.68-1.09)	0.21
Dizziness	1.01 (0.93-1.11)	0.80

Note: Major CVD: composite outcomes of heart failure, chronic heart disease and stroke; CHD: chronic heart disease; ESRD: end-stage renal disease; Analyses adjusted for sex, age, smoking status, fasting glucose, high-density lipoprotein cholesterol, low-density lipoprotein cholesterol, triglyceride, total cholesterol, eGFR, Charlson Comorbidities Index, usage of ACEI/ARB, β-blocker, calcium channel blockers, diuretic; history of adverse events, obesity status, specialist outpatient clinics attendance, general outpatient clinics attendance, accident and emergency attendance and hospitalization (within 1 year before baseline). Statistical significance was defined as a two-tailed *p*-value.

Supplementary Table 20 Estimated hazard ratios (and 95% confidence interval) of hypertension related complications, all-cause mortality and serious adverse events between intensive treatment target group and traditional treatment target group (Including patients with low-dose aspirin prescriptions on or before baseline)

Outcome	Adjusted hazard ratio (95% CI)	P value
Major CVD	0.93 (0.87-0.99)	0.02
CHD	0.91 (0.83-0.99)	0.04
Heart Failure	0.85 (0.72-1.01)	0.07
Stroke	0.97 (0.89-1.06)	0.53
ESRD	0.81 (0.67-0.99)	0.04
Mortality	0.89 (0.81-0.98)	0.02
Serious adverse event		
Composite of seven serious adverse event	0.99 (0.94-1.04)	0.73
Hypotension	1.01 (0.79-1.29)	0.93
Syncope	1.01 (0.88-1.15)	0.92
Bradycardia	1.02 (0.64-1.63)	0.95
Electrolyte abnormality	1.41 (0.33-5.99)	0.64
Falls	1.00 (0.92-1.08)	0.94
Acute kidney disease	0.88 (0.71-1.10)	0.27
Dizziness	1.01 (0.93-1.10)	0.78

Note: Major CVD: composite outcomes of heart failure, chronic heart disease and stroke; CHD: chronic heart disease; ESRD: end-stage renal disease; Analyses adjusted for sex, age, smoking status, fasting glucose, high-density lipoprotein cholesterol, low-density lipoprotein cholesterol, triglyceride, total cholesterol, eGFR, Charlson Comorbidities Index, usage of ACEI/ARB, β-blocker, calcium channel blockers, diuretic; history of adverse events, obesity status, specialist outpatient clinics attendance, general outpatient clinics attendance, accident and emergency attendance and hospitalization (within 1 year before baseline). Statistical significance was defined as a two-tailed *p*-value.

Supplementary Table 21 Estimated hazard ratios (and 95% confidence interval) of hypertension related complications, all-cause mortality and serious adverse events between intensive treatment target group and traditional treatment target group (Including the year of the enrolment in the weighting model)

Outcome	Adjusted hazard ratio (95% CI)	P value
Major CVD	0.80 (0.73-0.88)	<0.001
CHD	0.71 (0.62-0.82)	<0.001
Heart Failure	0.88 (0.70-1.11)	0.27
Stroke	0.89 (0.80-0.99)	0.03
ESRD	0.84 (0.62-1.12)	0.23
Mortality	0.88 (0.80-0.97)	0.01
Serious adverse event		
Composite of seven serious adverse event	0.99 (0.93-1.04)	0.65
Hypotension	1.01 (0.77-1.32)	0.94
Syncope	0.99 (0.86-1.14)	0.88
Bradycardia	0.93 (0.53-1.63)	0.80
Electrolyte abnormality	1.51 (0.34-6.70)	0.59
Falls	1.01 (0.93-1.10)	0.83
Acute kidney disease	0.86 (0.68-1.09)	0.22
Dizziness	1.00 (0.91-1.09)	0.98

Note: Major CVD: composite outcomes of heart failure, chronic heart disease and stroke; CHD: chronic heart disease; ESRD: end-stage renal disease; Analyses adjusted for sex, age, smoking status, fasting glucose, high-density lipoprotein cholesterol, low-density lipoprotein cholesterol, triglyceride, total cholesterol, eGFR, Charlson Comorbidities Index, usage of ACEI/ARB, β-blocker, calcium channel blockers, diuretic; history of adverse events, obesity status, specialist outpatient clinics attendance, general outpatient clinics attendance, accident and emergency attendance and hospitalization (within 1 year before baseline). Statistical significance was defined as a two-tailed *p*-value.

Supplementary Table 22 Estimated hazard ratios (and 95% confidence interval) of hypertension related complications, all-cause mortality and serious adverse events between intensive treatment target group and traditional treatment target group (Using two consecutive records to define deviation from the assigned treatment strategy)

Outcome	Adjusted hazard ratio (95% CI)	P value
Major CVD	0.86 (0.78-0.94)	<0.001
CHD	0.79 (0.68-0.91)	<0.001
Heart Failure	0.85 (0.67-1.09)	0.20
Stroke	0.92 (0.81-1.05)	0.22
ESRD	0.85 (0.65-1.10)	0.22
Mortality	0.86 (0.75-0.99)	0.03
Serious adverse event		
Composite of seven serious adverse event	0.98 (0.91-1.04)	0.47
Hypotension	1.17 (0.83-1.65)	0.36
Syncope	0.99 (0.84-1.18)	0.95
Bradycardia	0.49 (0.20-1.17)	0.11
Electrolyte abnormality	0.84 (0.20-3.56)	0.82
Falls	0.98 (0.91-1.06)	0.62
Acute kidney disease	0.80 (0.59-1.08)	0.15
Dizziness	1.02 (0.92-1.13)	0.73

Note: Major CVD: composite outcomes of heart failure, chronic heart disease and stroke; CHD: chronic heart disease; ESRD: end-stage renal disease; Analyses adjusted for sex, age, smoking status, fasting glucose, high-density lipoprotein cholesterol, low-density lipoprotein cholesterol, triglyceride, total cholesterol, eGFR, Charlson Comorbidities Index, usage of ACEI/ARB, β-blocker, calcium channel blockers, diuretic; history of adverse events, obesity status, specialist outpatient clinics attendance, general outpatient clinics attendance, accident and emergency attendance and hospitalization (within 1 year before baseline). Statistical significance was defined as a two-tailed *p*-value.

Supplementary Table 23 Estimated hazard ratios (and 95% confidence interval) of hypertension related complications, all-cause mortality and serious adverse events between intensive treatment target group and traditional treatment target group (Using three consecutive records to define deviation from the assigned treatment strategy)

Outcome	Adjusted hazard ratio (95% CI)	P value
Major CVD	0.86 (0.79-0.93)	<0.001
CHD	0.80 (0.70-0.91)	<0.001
Heart Failure	0.89 (0.72-1.10)	0.27
Stroke	0.91 (0.81-1.02)	0.11
ESRD	0.77 (0.61-0.98)	0.03
Mortality	0.87 (0.78-0.98)	0.02
Serious adverse event		
Composite of seven serious adverse event	0.98 (0.92-1.04)	0.48
Hypotension	1.09 (0.81-1.46)	0.59
Syncope	1.07 (0.92-1.24)	0.39
Bradycardia	0.51 (0.24-1.09)	0.08
Electrolyte abnormality	NA	NA
Falls	0.98 (0.90-1.07)	0.68
Acute kidney disease	0.84 (0.64-1.10)	0.21
Dizziness	1.02 (0.92-1.12)	0.74

Note: Major CVD: composite outcomes of heart failure, chronic heart disease and stroke; CHD: chronic heart disease; ESRD: end-stage renal disease; NA: convergency not achieved; Analyses adjusted for sex, age, smoking status, fasting glucose, high-density lipoprotein cholesterol, low-density lipoprotein cholesterol, triglyceride, total cholesterol, eGFR, Charlson Comorbidities Index, usage of ACEI/ARB, β-blocker, calcium channel blockers, diuretic; history of adverse events, obesity status, specialist outpatient clinics attendance, general outpatient clinics attendance, accident and emergency attendance and hospitalization (within 1 year before baseline). Statistical significance was defined as a two-tailed *p*-value.

Supplementary Table 24 Estimated hazard ratios (and 95% confidence interval) of hypertension related complications, all-cause mortality and serious adverse events between intensive treatment target group and traditional treatment target group (Using four consecutive records to define deviation from the assigned treatment strategy)

**Outcome**	**Adjusted hazard ratio (95% CI)**	**P value**
Major CVD	0.84 (0.78-0.91)	<0.001
CHD	0.80 (0.71-0.90)	<0.001
Heart Failure	0.86 (0.71-1.05)	0.13
Stroke	0.88 (0.79-0.98)	0.02
ESRD	0.80 (0.64-0.99)	0.04
Mortality	0.88 (0.79-0.98)	0.02
Serious adverse event		
Composite of seven serious adverse event	0.98 (0.92-1.03)	0.43
Hypotension	0.98 (0.74-1.30)	0.91
Syncope	1.02 (0.89-1.18)	0.75
Bradycardia	0.80 (0.43-1.48)	0.48
Electrolyte abnormality	1.47 (0.35-6.25)	0.60
Falls	0.99 (0.91-1.07)	0.76
Acute kidney disease	0.80 (0.62-1.03)	0.08
Dizziness	1.00 (0.92-1.10)	0.93

Note: Major CVD: composite outcomes of heart failure, chronic heart disease and stroke; CHD: chronic heart disease; ESRD: end-stage renal disease; Analyses adjusted for sex, age, smoking status, fasting glucose, high-density lipoprotein cholesterol, low-density lipoprotein cholesterol, triglyceride, total cholesterol, eGFR, Charlson Comorbidities Index, usage of ACEI/ARB, β-blocker, calcium channel blockers, diuretic; history of adverse events, obesity status, specialist outpatient clinics attendance, general outpatient clinics attendance, accident and emergency attendance and hospitalization (within 1 year before baseline). Statistical significance was defined as a two-tailed *p*-value.

Supplementary Table 25 Estimated hazard ratios (and 95% confidence interval) of hypertension related complications, all-cause mortality and serious adverse events between intensive treatment target group and traditional treatment target group (Using six consecutive records to define deviation from the assigned treatment strategy)

Outcome	Adjusted hazard ratio (95% CI)	P value
Major CVD	0.86 (0.80-0.92)	<0.001
CHD	0.83 (0.74-0.92)	<0.001
Heart Failure	0.87 (0.73-1.04)	0.14
Stroke	0.90 (0.81-0.99)	0.04
ESRD	0.82 (0.68-1.00)	0.05
Mortality	0.88 (0.80-0.97)	0.01
Serious adverse event		
Composite of seven serious adverse event	1.03 (0.98-1.09)	0.26
Hypotension	1.02 (0.78-1.33)	0.90
Syncope	1.07 (0.93-1.23)	0.35
Bradycardia	1.09 (0.63-1.88)	0.77
Electrolyte abnormality	0.95 (0.22-4.09)	0.95
Falls	1.08 (0.99-1.17)	0.08
Acute kidney disease	0.91 (0.72-1.16)	0.45
Dizziness	1.06 (0.97-1.16)	0.20

Note: Major CVD: composite outcomes of heart failure, chronic heart disease and stroke; CHD: chronic heart disease; ESRD: end-stage renal disease; Analyses adjusted for sex, age, smoking status, fasting glucose, high-density lipoprotein cholesterol, low-density lipoprotein cholesterol, triglyceride, total cholesterol, eGFR, Charlson Comorbidities Index, usage of ACEI/ARB, β-blocker, calcium channel blockers, diuretic; history of adverse events, obesity status, specialist outpatient clinics attendance, general outpatient clinics attendance, accident and emergency attendance and hospitalization (within 1 year before baseline). Statistical significance was defined as a two-tailed *p*-value.

Supplementary Table 26 Estimated hazard ratios (and 95% confidence interval) of hypertension related complications, all-cause mortality and serious adverse events between intensive treatment target group and traditional treatment target group patients (Considering side effect due to age, frailty or polypharmacy as contraindications)

Outcome	Adjusted hazard ratio (95% CI)	P value
Major CVD	0.85 (0.79-0.91)	<0.001
CHD	0.80 (0.72-0.90)	<0.001
Heart Failure	0.83 (0.69-0.99)	0.04
Stroke	0.90 (0.81-1.00)	0.05
ESRD	0.78 (0.64-0.96)	0.02
Mortality	0.88 (0.80-0.97)	0.01
Serious adverse event		
Composite of seven serious adverse event	1.03 (0.98-1.09)	0.26
Hypotension	1.02 (0.78-1.33)	0.90
Syncope	1.07 (0.93-1.23)	0.35
Bradycardia	1.09 (0.63-1.88)	0.77
Electrolyte abnormality	0.95 (0.22-4.09)	0.95
Falls	1.08 (0.99-1.17)	0.08
Acute kidney disease	0.91 (0.72-1.16)	0.45
Dizziness	1.06 (0.97-1.16)	0.20

Note: Major CVD: composite outcomes of heart failure, chronic heart disease and stroke; CHD: chronic heart disease; ESRD: end-stage renal disease; Analyses adjusted for sex, age, smoking status, fasting glucose, high-density lipoprotein cholesterol, low-density lipoprotein cholesterol, triglyceride, total cholesterol, eGFR, Charlson Comorbidities Index, usage of ACEI/ARB, β-blocker, calcium channel blockers, diuretic; history of adverse events, obesity status, specialist outpatient clinics attendance, general outpatient clinics attendance, accident and emergency attendance and hospitalization (within 1 year before baseline). Statistical significance was defined as a two-tailed *p*-value.

Supplementary Table 27 The proportion of times of intensification in each treatment strategy

	Year 1	Year 2	Year 3	Year 4	Year 5	Year 6	Year 7	Year 8	Year 9	Year 10	Year 11
Blood pressure target below 140/90 mmHg											
0	65.50	78.35	86.81	86.99	86.65	85.78	88.63	90.95	93.94	96.35	98.51
1	28.81	17.54	11.02	10.94	11.30	11.83	9.58	7.61	4.99	3.04	1.20
2	4.75	3.38	1.73	1.62	1.67	1.89	1.46	1.16	0.83	0.53	0.27
≥3	0.94	0.73	0.42	0.45	0.38	0.50	0.33	0.28	0.24	0.08	0.02
Blood pressure target below 130/80 mmHg											
0	57.56	67.23	78.29	81.74	83.32	84.43	88.09	91.31	94.19	96.71	98.61
1	33.25	25.23	17.54	15.04	13.78	12.94	9.85	7.20	4.76	2.69	1.12
2	7.55	6.02	3.38	2.60	2.36	2.13	1.68	1.20	0.83	0.47	0.22
≥3	1.64	1.52	0.79	0.62	0.54	0.50	0.38	0.29	0.22	0.13	0.05

Note: the score is calculated by adding up the times of dosage increase and number of antihypertensive drug increases per year during follow-up

Supplementary Table 28 The mean numbers of anti-hypertensive regiments in each treatment strategy over the follow-up years

	Year 0	Year 1	Year 2	Year 3	Year 4	Year 5	Year 6	Year 7	Year 8	Year 9	Year 10	Year 11
Blood pressure target below 140/90 mmHg												
Mean/SD	1.21/0.44	1.33/0.53	1.35/0.54	1.37/0.55	1.38/0.56	1.39/0.58	1.42/0.59	1.45/0.61	1.49/0.64	1.55/0.67	1.61/0.68	1.66/0.69
Blood pressure target below 130/80 mmHg												
Mean/SD	1.20/0.44	1.34/0.54	1.43/0.58	1.47/0.61	1.51/0.62	1.53/0.64	1.57/0.66	1.61/0.67	1.66/0.69	1.72/0.71	1.79/0.73	1.86/0.75

Supplementary Table 29 Model estimates in the inverse probability weighting

	Treatment strategy				Treatment arm: BP target of <130/80mmHg				Traditional arm: BP target of 130-140/80-90mmHg			
	Denominator		Nominator		Denominator		Nominator		Denominator		Nominator	
**Variable**	**Coef**	**SE**	**Coef**	**SE**	**Coef**	**SE**	**Coef**	**SE**	**Coef**	**SE**	**Coef**	**SE**
Intercept	-1.661†	0.032	-1.796†	0.030	7.012†	0.301	5.527	0.259	9.797†	0.180	8.408†	0.138
Month	-0.006	0.001	-0.001	0.001	-0.054†	0.005	-0.039	0.002	-0.061†	0.003	-0.057†	0.001
Month square	0.0003	0.0002	-0.002	0.002	0.0003†	0.0000	0.0003	0.0001	0.0004†	0.0000	0.0005†	0.0005
**Baseline variables**												
Gender, male	-0.339†	0.004	-0.351†	0.004	-0.266†	0.035	-0.196†	0.034	0.318†	0.020	0.305†	0.019
Age, year	0.008†	0.0002	0.008†	0.0002	-0.159	0.053	0.004†	0.002	-0.081†	0.030	-0.011†	0.001
Smoking status	0.084†	0.007	0.088†	0.007	-0.032	0.062	-0.003	0.061	-0.144†	0.036	-0.131†	0.036
Fasting glucose	0.012†	0.005	0.007†	0.001	0.003	0.021	0.040†	0.015	-0.030†	0.013	0.038†	0.009
Triglyceride level	-0.014†	0.005	-0.024†	0.004	0.039	0.036	0.028	0.032	0.011	0.019	0.021	0.020
HDL-C	-0.112†	0.026	-0.026†	0.010	0.273†	0.106	0.160	0.084	0.160†	0.060	0.059	0.053
LDL-C	-0.043†	0.014	-0.038†	0.009	0.038	0.085	0.020	0.076	0.019	0.048	0.018	0.049
Total cholesterol	0.110†	0.013	0.030†	0.009	0.056	0.083	-0.003	0.074	-0.096†	0.047	-0.059	0.048
CCI	-0.028†	0.004	0.049†	0.003	-0.005	0.041	0.015	0.030	-0.961†	0.046	0.024	0.019
ACEI/ARB user	-0.075†	0.008	-0.102†	0.007	-0.267†	0.064	-0.278	0.060	-0.111†	0.035	-0.194†	0.034
Beta blocker user	-0.105†	0.005	-0.006	0.004	-0.295†	0.043	-0.202	0.040	0.060†	0.025	-0.056†	0.023
CCB user	-0.167†	0.004	-0.174†	0.004	-0.113†	0.040	-0.078	0.039	0.076†	0.022	0.055†	0.022
Diuretic user	-0.221†	0.008	-0.180†	0.007	-0.369†	0.061	-0.320	0.059	0.122†	0.037	0.081†	0.036
eGFR	-0.0004	0.0003	-0.001†	0.002	-0.001	0.002	-0.002	0.001	-0.001	0.001	-0.008†	0.001
Obesity	-0.059†	0.020	-0.307†	0.005	0.059	0.073	-0.125†	0.047	-0.059	0.040	0.138†	0.026
Adverse events	-0.016	0.011	-0.039†	0.010	0.283†	0.107	0.170	0.096	0.029	0.061	0.055	0.053
A&E attendance	0.095†	0.005	0.079†	0.004	0.100†	0.040	0.096†	0.039	-0.038	0.023	-0.014	0.023
Inpatient Visit	-0.009	0.008	0.014†	0.006	-0.173†	0.055	-0.170†	0.055	0.005	0.033	0.031	0.033
GOPC Visit	-0.156†	0.042	-0.030†	0.014	1.408†	0.112	0.487†	0.097	1.240†	0.068	0.231†	0.062
SOPC Visit	-0.069†	0.006	0.0005	0.006	0.022	0.055	0.029	0.055	-0.039	0.031	-0.031	0.031
**Time-varying variables**			-1.796	0.030								
Smoking status	0.084	0.007			-0.032	0.062			-0.144†	0.036		
Fasting glucose	-0.041†	0.005			-0.072†	0.020			0.032†	0.013		
Triglyceride level	-0.014	0.005			-0.033	0.045			0.000	0.029		
HDL-C	0.121†	0.026			-0.087	0.124			-0.250†	0.078		
LDL-C	0.038†	0.016			-0.007	0.113			-0.106	0.070		
Total cholesterol	-0.119†	0.015			-0.156	0.112			0.195†	0.070		
CCI	0.061†	0.003			0.007	0.027			0.975†	0.042		

ACEI/ARB user	-0.016†	0.005	0.097	0.043	-0.188†	0.021
Beta blocker user	0.179†	0.005	0.278	0.041	-0.219†	0.021
Calcium channel blockers user	0.012†	0.005	0.239	0.044	-0.029	0.025
Diuretic user	0.107†	0.006	0.351	0.064	-0.072†	0.032
eGFR	-0.0002	0.000	-0.002	0.002	-0.012†	0.001
Obesity	-0.263†	0.020	-0.292†	0.070	0.296†	0.040
Adverse events	-0.036†	0.006	-0.122†	0.055	0.022	0.034
A&E attendance	-0.026†	0.005	0.057	0.043	0.049	0.025
Inpatient Visit	-0.023†	0.008	-0.039	0.061	0.261†	0.039
GOPC Visit	0.138†	0.043	-1.445†	0.110	-1.672†	0.068
SOPC Visit	0.153†	0.004	0.056	0.032	0.005	0.019

†P<0.05; coef: coefficient, SE: standard error

Notes: CCI: Charlson Comorbidity Index, CCB: Calcium channel blockers, A&E: Accident and emergency, GOPC: General Out-patient; SOPC: Specialist Out-patient; ACEI/ARB: angiotensinconverting enzyme inhibitor and angiotensin receptor blocker; Number of Specialist, General Outpatient Clinics attendance, accident and emergency and hospitalization were counted within 1 year before baseline. Statistical significance was defined as a two-tailed *p*-value.

Supplementary Figure 1 Definition of indicators for different types of patients regarding their timeline

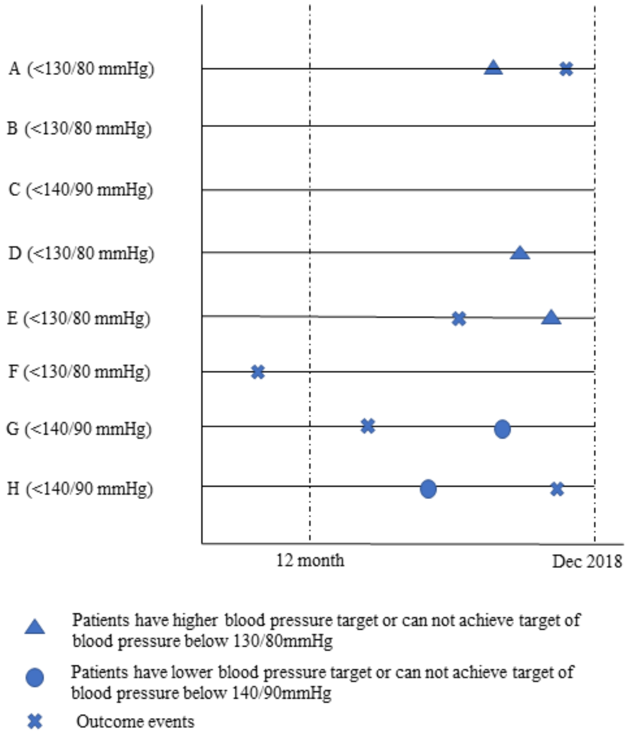

	Arm	Value of indicators		
		Censoring	Outcome	End of follow-up
A	Treatment	1	0	Time to censor
	Control	1	0	12m
B	Treatment	0	0	Time to end date
	Control	1	0	12m
C	Treatment	0	0	12m
	Control	0	0	Time to end date
D	Treatment	1	0	Time to censor
	Control	1	0	12m
E	Treatment	0	1	Time to outcome
	Control	1	0	12m
F	Treatment	0	1	Time to outcome
	Control	0	1	Time to outcome
G	Treatment	1	0	12m
	Control	0	1	Time to outcome
H	Treatment	1	0	12m
	Control	1	0	Time to censor

Supplementary Figure 2 Proportion of patients who adhered to the assigned treatment target during follow-up period

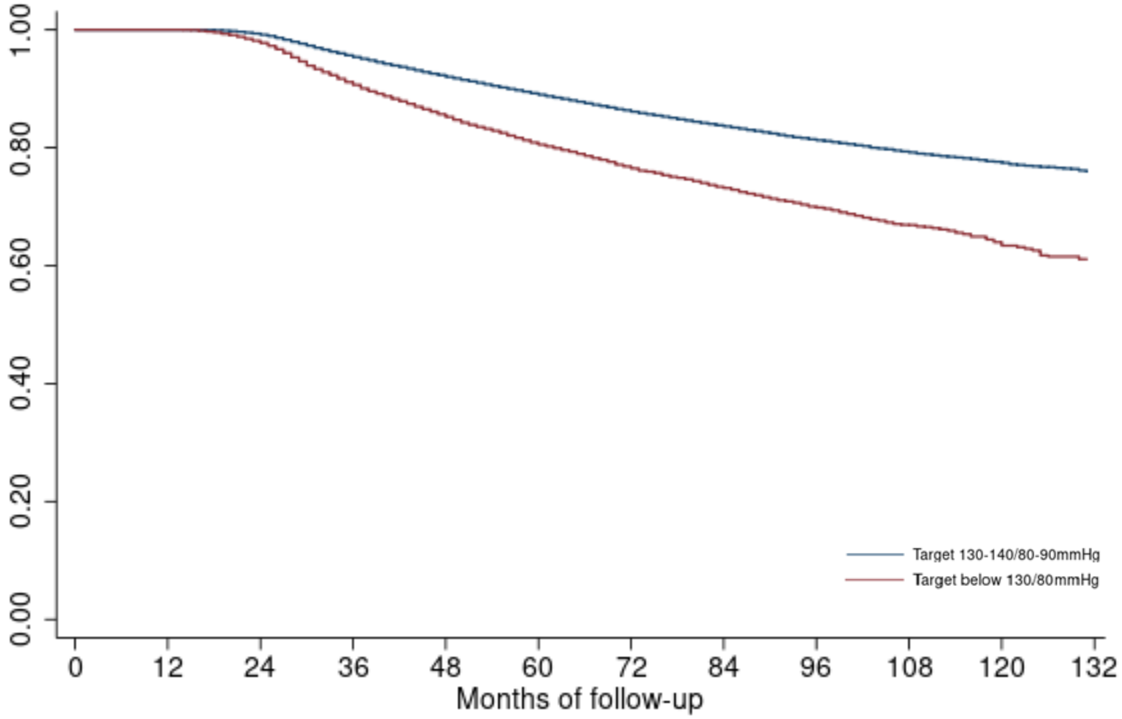

Supplementary Figure 3 Systolic blood pressure and diastolic blood pressure during follow-up

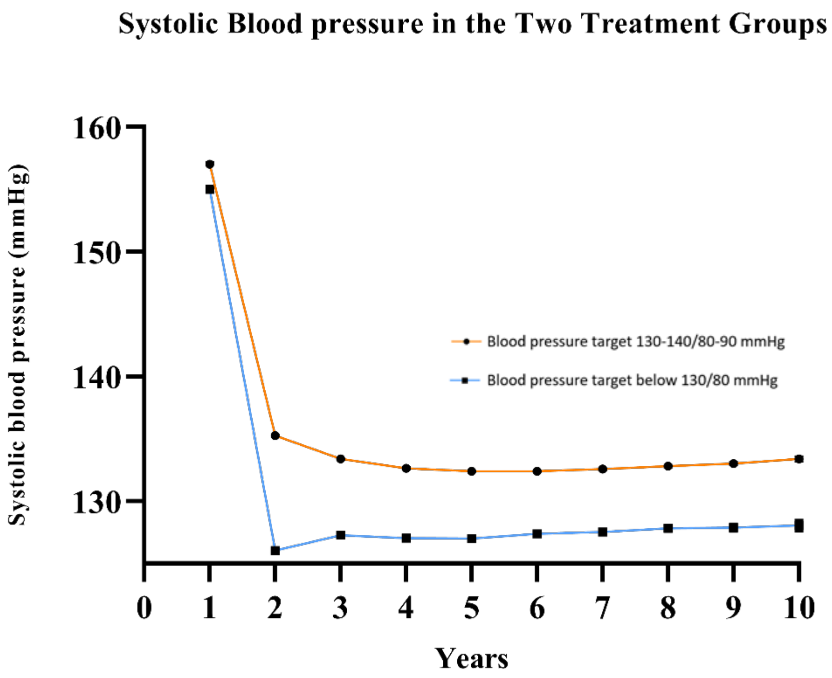

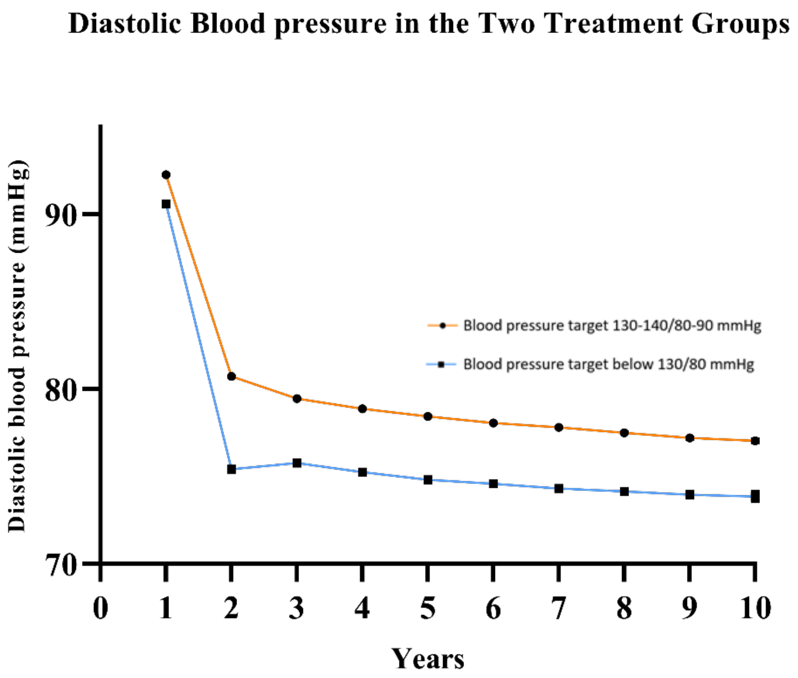

Supplement: Supplementary file 1 — Supplementary Information [file 41467_2026_74041_MOESM1_ESM.pdf]
