# Supplementary material for: Optimal blood pressure target in patients with uncomplicated hypertension: a target trial emulation study
Source: Nat Commun. 2026 Jun 5;17:7207. doi: 10.1038/s41467-026-74041-9 (PMC13396347; doi:10.1038/s41467-026-74041-9)
Supplement: Supplementary file 2 — Reporting Summary [file 41467_2026_74041_MOESM2_ESM.pdf]

nature portfolio

Corresponding author(s): Eric Yuk Fai Wan

Last updated by author(s): Feb 2, 2026

Reporting Summary

Nature Portfolio wishes to improve the reproducibility of the work that we publish. This form provides structure for consistency and transparency in reporting. For further information on Nature Portfolio policies, see our Editorial Policies and the Editorial Policy Checklist.

Statistics

For all statistical analyses, confirm that the following items are present in the figure legend, table legend, main text, or Methods section.

n/a	Confirmed	
☐	☒	The exact sample size (n) for each experimental group/condition, given as a discrete number and unit of measurement
☒	☐	A statement on whether measurements were taken from distinct samples or whether the same sample was measured repeatedly
☐	☒	The statistical test(s) used AND whether they are one- or two-sided<br>*Only common tests should be described solely by name; describe more complex techniques in the Methods section.*
☐	☒	A description of all covariates tested
☐	☒	A description of any assumptions or corrections, such as tests of normality and adjustment for multiple comparisons
☐	☒	A full description of the statistical parameters including central tendency (e.g. means) or other basic estimates (e.g. regression coefficient) AND variation (e.g. standard deviation) or associated estimates of uncertainty (e.g. confidence intervals)
☐	☒	For null hypothesis testing, the test statistic (e.g. F, t, r) with confidence intervals, effect sizes, degrees of freedom and P value noted<br>*Give P values as exact values whenever suitable.*
☒	☐	For Bayesian analysis, information on the choice of priors and Markov chain Monte Carlo settings
☐	☒	For hierarchical and complex designs, identification of the appropriate level for tests and full reporting of outcomes
☒	☐	Estimates of effect sizes (e.g. Cohen's d, Pearson's r), indicating how they were calculated

Our web collection on statistics for biologists contains articles on many of the points above.

Software and code

Policy information about availability of computer code

Data collection	The data was extracted by the data custodian (Hong Kong Hospital Authority).
Data analysis	All analyses were performed in Stata/MP 17.0. The code used in this study is available on Zenodo (https://doi.org/10.5281/zenodo.17033442).

For manuscripts utilizing custom algorithms or software that are central to the research but not yet described in published literature, software must be made available to editors and reviewers. We strongly encourage code deposition in a community repository (e.g. GitHub). See the Nature Portfolio guidelines for submitting code & software for further information.

Data

Policy information about availability of data

All manuscripts must include a data availability statement. This statement should provide the following information, where applicable:

- Accession codes, unique identifiers, or web links for publicly available datasets
- A description of any restrictions on data availability
- For clinical datasets or third party data, please ensure that the statement adheres to our policy

The data contains confidential information and hence cannot be shared with the public due to third-party use restrictions.

Research involving human participants, their data, or biological material

Policy information about studies with human participants or human data. See also policy information about sex, gender (identity/presentation), and sexual orientation and race, ethnicity and racism.

Reporting on sex and gender	This study reported subgroup analysis on participants of male and female. The sex of each participants were determined based on the electronic healthcare records from the Hong Kong Hospital Authority .
Reporting on race, ethnicity, or other socially relevant groupings	The manuscript did not report on race, ethnicity, or other socially relevant groupings of participants.
Population characteristics	A total of 118,271 patients with hypertension were included in this study (Figure 1). After censoring due to treatment deviation over the grace period, 82,753 patients initiated an optimal BP target of BP 130-140/80-90 mmHg and 15,992 initiated BP of 120-130/70-80 mmHg were assigned into the separate treatment arms. The mean age was 57.6 (SD 10.2) and 58.6 (SD 9.7) and BP was 157.1 (16.6)/92.3(8.9) mmHg and 155.1 (15.9)/90.6(8.0) mmHg of patients in traditional treatment strategy and in the intensive treatment strategy, respectively. We have provided all details in Table 1.
Recruitment	Electronic healthcare records from clinical management system provided by the Hong Kong Hospital Authority.
Ethics oversight	Ethical approval for this study was granted by the Institutional Review Board of the University of HK/HA HK West Cluster (UW19-361) with an exemption for informed consent from participants as patients' confidentiality was maintained in this retrospective cohort study.

Note that full information on the approval of the study protocol must also be provided in the manuscript.

Field-specific reporting

Please select the one below that is the best fit for your research. If you are not sure, read the appropriate sections before making your selection.

☒ Life sciences ☐ Behavioural & social sciences ☐ Ecological, evolutionary & environmental sciences

For a reference copy of the document with all sections, see nature.com/documents/nr-reporting-summary-flat.pdf

Life sciences study design

All studies must disclose on these points even when the disclosure is negative.

Sample size	Sample size calculation was not concluded. This is a population-based study and all identified eligible patients from the territory-wide healthcare database were included in this study. A total of 118,271 patients with diagnosis of hypertension identified from the clinical management system (CMS) provided by the Hong Kong Hospital Authority (HKHA), on or before the end of 2013 were included in this study.
Data exclusions	Patients on 4 or more regular medications on or within 3 months before baseline, patients with history of CKD, DM or CVD were excluded in this study. Complete case analysis was conducted, patients with incomplete data for the used covariates at the baseline were also excluded. Individuals with a prescription for aspirin on or before baseline were excluded.
Replication	Three investigators conducted each of the statistical analyses independently for quality insurance and obtained the results.<br>Local academic institutions, government departments, or non-governmental organizations may apply for the access to data through the Hospital Authority's data sharing portal (https://www3.ha.org.hk/data) and replicate this analysis.<br>The codes for this study are available online on Zenodo (https://doi.org/10.5281/zenodo.17033442).
Randomization	An observational study was conducted, hence exposure allocation was not random. However, we used clone-censor-weight approach to emulate the randomization.
Blinding	An observational study was conducted, hence blindings were not applicable.

Reporting for specific materials, systems and methods

We require information from authors about some types of materials, experimental systems and methods used in many studies. Here, indicate whether each material, system or method listed is relevant to your study. If you are not sure if a list item applies to your research, read the appropriate section before selecting a response.

Materials & experimental systems

n/a	Involved in the study
☒	☐ Antibodies
☒	☐ Eukaryotic cell lines
☒	☐ Palaeontology and archaeology
☒	☐ Animals and other organisms
☒	☐ Clinical data
☒	☐ Dual use research of concern
☒	☐ Plants

Methods

n/a	Involved in the study
☒	☐ ChIP-seq
☒	☐ Flow cytometry
☒	☐ MRI-based neuroimaging

Plants

Seed stocks	An observational study was conducted, hence this information is not applicable.
Novel plant genotypes	An observational study was conducted, hence this information is not applicable.
Authentication	An observational study was conducted, hence this information is not applicable.
