# Supplementary material for: Optimal blood pressure target in patients with uncomplicated hypertension: a target trial emulation study
Source: Nat Commun. 2026 Jun 5;17:7207. doi: 10.1038/s41467-026-74041-9 (PMC13396347; doi:10.1038/s41467-026-74041-9)
Supplement: Supplementary file 3 — Transparent Peer Review file [file 41467_2026_74041_MOESM3_ESM.pdf]

nature portfolio

Peer Review File

Optimal blood pressure target in patients with uncomplicated hypertension: a target trial emulation study

Corresponding Author: Dr Eric Yuk Fai Wan

This file contains all reviewer reports in order by version, followed by all author rebuttals in order by version.

Version 0:

Reviewer comments:

Reviewer #1

(Remarks to the Author)
Summary
This study evaluated CVD risk reduction and safety in uncomplicated hypertension managed by intensive or standard BP targets. The authors conducted a target trial emulation involving 98745 patients without CKD, DM or CVD and prescription adjustment between Jan 2008 to Dec 2013 using public electronic medical records. Patients were assigned to two treatment arms of BP targets, that is, <140/90mmHg or <130/80 mmHg according to their prescription and BP records after 1-year grace period. Pooled logistic regression was applied to obtain the hazard ratios (HRs) for the major CVDs (stroke, coronary heart disease, heart failure), all-cause mortality and major adverse events (hypotension, syncope, electrolyte abnormalities, and acute kidney injury or failure). Of the 98745 eligible patients, 82753 were managed by the treatment target of 140/90mmHg and 15992 by the treatment goal of 130/80mmHg. Over the 7-years of follow-up, patients with a lower BP target had lower risk of CVDs (hazard ratio [HR]: 0.85 [95%CI, 0.79 to 0.91]) and all-cause mortality (hazard ratio [HR]: 0.88 [95%CI, 0.80 to 0.97]) with no significant increase of adverse events. The authors concluded that intensive BP management (<130/80mmHg) in real-world settings has higher clinical benefit than standard BP management (<140/90 mmHg).
Comments
Introduction: Ref. 1 is unclear to me.
Introduction (lines 96-107 and references 6-14): I think this part of the manuscript needs to be better written. The sequence and appropriateness of citations should be more in line with the appearance in the literature. Some numbers should be double-checked and the data on the global SPRINT study and in the elderly should be distinguished. There is a lack of reference for the European Society of Hypertension (ESH) guidelines.
Introduction: the aim of the study could be better written.
Please, check Ref. 12
Methods are reported after Results and Discussion: is there any reason?
If I am not mistaken, you have defined hypertension (inclusion criteria) as BP >= 130/80 mmHg, but you have established two treatment targets, i.e. < 140/90 mmHg or < 130/80 mmHg. This is not entirely clear to me.
Could you explain better why approximately 83000 patients were assigned to the traditional target and approximately 16000 patients were assigned to the intensive target?
You state: “The crude incidence rate for each outcome is summarized inTable S3.” Please check.
Lines 122-124: “Over 60% of the patients with intensive blood pressure target adhered to the treatment strategy while 20% of the patients with traditional treatment target did not achieve or considered a lower blood pressure target (Figure S2).” This sentence is unclear to me.
Please check the title of Figure 2. Also, this figure appears to be a duplicate of Table 2.
I cannot find Figure 3 in the text.
Supplementary Table 8: Please comment on the results on CHD risk according to age ranges.
Refs 9 and 17 are the same.
Refs 13 and 21 are the same.
At least for me, it is difficult to follow some parts of the discussion section which should be overall shortened and focused on the main findings.
There are some typos.
English language should be imroved.

(Remarks on code availability)

Reviewer #2

(Remarks to the Author)
I appreciate the opportunity to review the manuscript "Optimal blood pressure target for antihypertensive management in patients with uncomplicated hypertension: Real-world evidence from a target trial emulation study". The authors conducted a target trial emulation to evaluate the benefits of the cardiovascular diseases risk reduction in hypertension patients managed by intensive compared to standard BP target using real-world electronic health records in Hong Kong. Overall, the manuscript is well written. However, there are some points that require work to improve the clearness and readability. Please find below some questions and suggestions:

1. The authors defined intensive treatment as two consecutive records of BP <130/80 mmHg without de-escalation or BP 130-140/ 80-90 mmHg with escalation of drugs treatment. This seems to be arbitrary. I would suggest providing the rationale of using this definition and adding some sensitivity analyses.
2. The outcomes were obtained based on coding of disease diagnoses. The adverse events were defined as the diagnosis that required hospital admission. This may underestimate the risk of adverse outcomes, as patients with some adverse events, such as dizziness may not be hospitalized or be reported as a discharge diagnosis.
3. A negative control outcome, for example, cancer incidence would be considered. The conclusion of this study would be strengthened, if no association was found between BP lowering treatments and cancer incidence.
4. It is not clear for the choice of variables in the inverse probability weighting. It seems that a few major potential confounders are not included. For example, the baseline BP levels, duration of hypertension diagnosis, alcohol consumption, diet, and physical activity.
5. The authors might consider deaths from non-cardiovascular events competing risks to obtain the effects of BP-lowering interventions.
6. Individuals with a prescription for aspirin on or before baseline were excluded to avoid the under-diagnosis of CVD history. However, aspirin is still widely used in primary prevention during 2008 to 2013.

(Remarks on code availability)

Reviewer #3

(Remarks to the Author)
In this manuscript, Zhang et al. report the results of a target trial emulation study using real-world electronic health record data to evaluate optimal blood pressure targets for patients with uncomplicated hypertension. The hypothesis is clearly stated, and the manuscript is organized and overall well-written. The authors showed that the group assigned to intensive blood pressure target had significantly lower risk of cardiovascular events and mortality compared to those assigned to the standard target group over 7 years of follow-up.

I agree with two of the strengths espoused by the authors: 1) the use of target trial emulation methodology, which provides a rigorous framework for analyzing observational data to approximate a randomized controlled trial; and 2) the large sample size (98,745 patients) with extended follow-up, which provides robust real-world evidence. The authors further employed sensitivity analyses and subgroup analyses to strengthen the findings.

There are several major concerns noted below:

Major Concerns:

1. I have several queries regarding treatment assignment. Time zero is defined as the "first date of prescription adjustment," and subjects are given a 12-month grace period to determine eligibility. It appears that treatment strategies were defined retrospectively based on observed clinician prescriptions and BP trends, rather than according to a pre-specified protocol. This suggests a post-treatment classification, where the treatment group is inferred from downstream outcomes (i.e., BP trends and medication escalation). This approach raises concerns about potential reverse causation, where BP readings influence medication decisions, yet are treated as independent indicators of the assigned strategy. Additionally, it appears that all subjects with BP >140/90 mmHg are automatically considered to be on the "standard" treatment strategy. Given that this is an EHR-based study, it may be possible to incorporate clinical notes documenting physicians' explicitly stated BP targets to classify patients based on intended treatment strategies rather than observed patterns alone.

2. Enrollment in this target emulation trial began in 2008, well before publication of major randomized controlled trials (e.g., SPRINT, PBROAD, ESPRIT) showing lower BP was beneficial. How do the authors account for the rationale for lower BP in the intensive group? What was the distribution of enrollment dates in both groups by year? This could introduce major confounding as recommendations for SBP < 130 mmHg were not guideline based at the time of observation. Of course, widespread adoption of guidelines after publication are slow, but least there was a rationale.

3. The 12-month grace period may introduce bias. While the authors attempt to account for immortal time bias using cloning and censoring, the bias may still persist. Subjects must survive the entire 12-month grace period to be eligible, which could exclude those who experience clinical outcomes during this period. Moreover, only patients with consistent follow-up data—specifically, two consecutive BP measurements—are eligible, though the interval between these measurements is not

clearly defined. This could lead to survivorship bias, where only patients actively engaged in their healthcare remain in the cohort, while sicker patients may have “dropped out” during the grace period.

4. The censoring criteria seem confusing. For the standard BP target strategy clones, they are censored at month 12 if they had 5 consecutive records considering lower blood pressure target (identified as patients with BP below 140/90mmHg with prescription escalation). The rationale behind using 5 records and the specific time window is not clearly explained. These censoring rules are extensive and may reintroduce selection bias, undermining the very purpose of the cloning. For example, why 5 consecutive visits? Why not, e.g. 4?

5. The inclusion of medical exceptions for diabetes and CKD is appropriate, but the absence of consideration for other conditions—such as orthostatic hypotension, frailty, or medication intolerance—imposes a rigid rule-based framework that may not reflect real-world clinical practice. This limits the generalizability of the findings and may violate the positivity assumption, which requires that each participant have a non-zero probability of receiving any of the treatment strategies. For example, a frail elderly patient with hypotension might need de-escalation, but without DM or CKD, they may be censored. This implies that patients who are more susceptible to adverse effects—due to age, comorbidities, or polypharmacy—could never have feasibly received intensive treatment, even though in real practice, they might have through individualized clinical judgment. This violation has downstream implications: the treatment strategies may not represent the full covariate distribution, and inverse probability of censoring weighting (IPCW) may be biased due to extrapolation beyond the observed data, ultimately distorting per-protocol effect estimates.

6. Although the authors mention complete-case analysis, the extent and pattern of missing data—particularly regarding follow-up BP measurements—are not reported. It would be helpful to know how many BP measurements were available per patient and the method of BP assessment (e.g., ambulatory BP monitoring [ABPM], attended vs. unattended office readings).

7. Clarification is needed on how medication intensity and escalation are defined. It would be helpful to use a standardized score to capture treatment intensity, based on the number and dosages of antihypertensive medications. Supplementary Table 13 lacks clarity regarding the frequency and definition of intensification. Does intensification refer to the number of medications, dose increases, or changes to more potent drug classes? According to Supplementary Figure 3, most subjects reached their BP goals within two years. However, Supplementary Table 13 reports that more than 80% of patients in both arms experienced three or more treatment intensifications by year 11. This seems inconsistent and warrants clarification.

8. The lack of increased adverse events warrants more detailed discussion and analysis. Given the issues raised above, one wonders whether the study cohort enrolled relatively healthier or lower-risk individuals.

9. Why so few medications from standard drug classes? Were those in intensive arm on more meds than those in standard arm? (e.g., similar to SPRINT, ~2.8 vs. 1.8)

Minor Concerns:

1. It would be useful to present the results of sensitivity analyses, model specifications (including variables used and regression coefficients), and the impact of weight truncation on outcomes.

2. For CONSORT diagram, in Figure 1, the ineligible patients + eligible patients (N=118,721) do not add up to N=699,568. Please explain this discrepancy.

(Remarks on code availability)

Reviewer #4

(Remarks to the Author)
I co-reviewed this manuscript with one of the reviewers who provided the listed reports. This is part of the Nature Communications initiative to facilitate training in peer review and to provide appropriate recognition for Early Career Researchers who co-review manuscripts.

(Remarks on code availability)

Version 1:

Reviewer comments:

Reviewer #1

(Remarks to the Author)
The authors answered most of the questions, and the manuscript improved. In my opinion, some minor changes are needed.
Abstract: there are some typos (also in the text).
Introduction: At least in my opinion, it would be more appropriate to list references from 5 to 10 in order of their appearance in

the literature (8-10-9-7-6-5).
Abstract: “The absolute risk reduction observed for major CVDs was -0.44 % (CI, -1.47 to -0.44 %), and for all cause was -0.84 % (CI, -1.17 to -0.52 %).”, and Results: “The risk reduction (Table 2) was observed for major CVD (absolute risk difference, -0.95 % [CI, -1.47 to -0.44 %]), and all-cause mortality (absolute risk difference, -0.84 % [CI, -1.17 to -0.52 %]).” Please clarify.
You report: “Table 2 Hazard ratios estimate of different blood pressure treatment targets on the risk of hypertension related complications”. HRs do not appear to be listed in this table. Please check.
You report: “Our findings regarding the safety profile are generally consistent with those of the latest RCT conducted in China.13” Is this reference correct?

(Remarks on code availability)

Reviewer #2

(Remarks to the Author)
The authors have made considerable efforts to address the reviewers' comments. However, several important concerns remain:

After adjusting for competing risks, the association between intensive BP control and reduced risk of major CVD was attenuated, and it became non-significant for stroke and ESRD.

The inclusion of outpatient diagnoses revealed a significantly higher risk of syncope and dizziness associated with intensive treatment.

These findings are not consistent with the results of the main analysis, and therefore do not support the conclusion that an intensive BP target is associated with a lower risk of hypertension-related complications without a significant increase in adverse events.

(Remarks on code availability)

Reviewer #3

(Remarks to the Author)
In this revised manuscript, Zhang et al. report the use of target trial emulation methodology is appropriate and timely. The investigators should be commended for including a large sample size with an extended follow-up period of up to 7 years, providing robust real-world evidence.
The authors provided a detailed explanation of the time-zero definition and grace period rationale. They further conducted sensitivity analyses (6-month grace period, single record, 3 consecutive records), which showed consistent results. The provided-reported BP treatment goal was unavailable in the database and is now reported as a limitation of the study.

We also commend the authors' effort in demonstrating the enrollment distribution of BP targets, which showed consistent proportions across years (2008-2012). This suggests that there is no temporal bias in treatment assignment.

Regarding the censoring criteria that use 5 consecutive BP records for defining deviation from the assigned treatment protocol, the authors conducted sensitivity analyses using 2, 3, 4, and 6 consecutive records as the censoring criteria which showed consistent results. In terms of the rigid censoring rules that may violate the positivity assumption for vulnerable patients, the authors performed sensitivity analysis considering age, frailty, and polypharmacy as contraindications, and again the results remained consistent. Given that each individual is unique and statistical aggregates should not override clinical acumen, the authors adequately addressed this concern and acknowledged the limitation in the Discussion.

The authors emphasized the use of the "Clone-Censor-Weight" approach to address immortal time bias, selection bias, and confounding variables, but fundamentally, the study infers treatment strategy (intensive versus standard) from downstream observations (BP readings + medication changes) rather than from actual clinical intent of the physician. Hence, the classification rules may not capture actual physician treatment goal. This is acknowledged as a limitation.

Some persistent concerns include the lack of generalizability, as the study excluded patients with diabetes, CKD, and CVD, which limits applicability to real-world hypertensive patients. Nonetheless, the authors did acknowledge this limitation and provided a sensitivity analysis based on a generalized morbidity index. The study provides valuable real-world evidence, and the study results should be interpreted with appropriate caution by the reader.

We have no additional major or minor concerns.

(Remarks on code availability)

Version 2:

Reviewer comments:

Reviewer #2

(Remarks to the Author)
The authors have addressed my previous concerns regarding competing risks from non-CVD mortality and the higher risk associated with mild syncope or dizziness. The limitations have been appropriately acknowledged, and the conclusions have been revised with greater precision. I have no additional concerns.

(Remarks on code availability)

Open Access This Peer Review File is licensed under a Creative Commons Attribution 4.0 International License, which permits use, sharing, adaptation, distribution and reproduction in any medium or format, as long as you give appropriate credit to the original author(s) and the source, provide a link to the Creative Commons license, and indicate if changes were made.
In cases where reviewers are anonymous, credit should be given to 'Anonymous Referee' and the source.
The images or other third party material in this Peer Review File are included in the article's Creative Commons license, unless indicated otherwise in a credit line to the material. If material is not included in the article's Creative Commons license and your intended use is not permitted by statutory regulation or exceeds the permitted use, you will need to obtain permission directly from the copyright holder.
To view a copy of this license, visit https://creativecommons.org/licenses/by/4.0/

RE: NCOMMS-25-28778 Optimal blood pressure target for antihypertensive management in patients with uncomplicated hypertension: Real-world evidence from a target trial emulation study

On behalf of the co-authors, I would like to thank the reviewers for their further comments and suggestions. In response, we have made the necessary adjustments in the revised manuscript, and hope that you will now find it suitable for publication. We provide here, our point-to-point responses to the earlier version of this paper reviewed.

<u>Reviewer 1</u>

R1(1):	This study evaluated CVD risk reduction and safety in uncomplicated hypertension managed by intensive or standard BP targets. The authors conducted a target trial emulation involving 98745 patients without CKD, DM or CVD and prescription adjustment between Jan 2008 to Dec 2013 using public electronic medical records. Patients were assigned to two treatment arms of BP targets, that is, <140/90mmHg or <130/80 mmHg according to their prescription and BP records after 1-year grace period. Pooled logistic regression was applied to obtain the hazard ratios (HRs) for the major CVDs (stroke, coronary heart disease, heart failure), all-cause mortality and major adverse events (hypotension, syncope, electrolyte abnormalities, and acute kidney injury or failure). Of the 98745 eligible patients, 82753 were managed by the treatment target of 140/90mmHg and 15992 by the treatment goal of 130/80mmHg. Over the 7-years of follow-up, patients with a lower BP target had lower risk of CVDs (hazard ratio [HR]: 0.85 [95%CI, 0.79 to 0.91]) and all-cause mortality (hazard ratio [HR]: 0.88 [95%CI, 0.80 to 0.97]) with no significant increase of adverse events. The authors concluded that intensive BP management (<130/80mmHg) in real-world settings has higher clinical benefit than standard BP management (<140/90 mmHg).
A:	*Thank you for your helpful comments and suggestions. Please find our response below.*

R1(2):	Introduction: Ref. 1 is unclear to me.
A:	*Thank you very much for your comment. We have revised the inserted citation and changed it to a more relevant reference to provide the more further information on the article cited in the manuscript.*

R1(3):	Introduction (lines 96-107 and references 6-14): I think this part of the manuscript needs to be better written. The sequence and appropriateness of citations should be more in line with the appearance in the literature. Some numbers should be double-checked and the data on the global SPRINT study and in the elderly should be distinguished. There is a lack of reference for the European Society of Hypertension (ESH) guidelines.
A:	*Thank you very much for your comments. We have revised the relevant paragraph of the introduction to review the findings and evidence from the literature. Furthermore, we have cited the reference on the ESH guideline mentioned in the manuscript.*

R1(4):	Introduction: the aim of the study could be better written.
A:	*Thank you very much for your suggestions. We have revised the description of the aim of this study in the manuscript as below:*<br><br>*This study aimed to evaluate the long-term benefits of intensified blood pressure control in reducing CVD complication and all-cause mortality rates. The results can inform clinical decisions on the optimal blood pressure target for patients with uncomplicated hypertension. (Page 7, Lines 115-118)*

R1(5):	Please, check Ref. 12
A:	*Thank you very much for your comment. We have checked and revised the format of Ref.12 accordingly.*

R1(6):	Methods are reported after Results and Discussion: is there any reason?
A:	*Thank you for your comment. The current structure was adopted based on the formatting guidelines stated by the nature communication. For your reference, please see the link below for the formatting guidelines in the author instructions for Nature Communications:*<br><br>*https://www.nature.com/documents/ncomms-submission-guide.pdf.*

R1(7):	If I am not mistaken, you have defined hypertension (inclusion criteria) as BP >= 130/80 mmHg, but you have established two treatment targets, i.e. < 140/90 mmHg or < 130/80 mmHg. This is not entirely clear to me.
A:	*Thank you very much for your comment. The aim of this study is to investigate the traditional target of 130-140/80-90mmHg versus lower BP target (<130/80 mmHg) and we also considered intensification on drug usage when defining the treatment arm. We would like to explain more on our definition for the baseline and the treatment assignment below:*<br>*1. For the baseline: patients with diagnosis of hypertension defined by ICPC-2 or ICD-9 code on or before the end of 2013 were included in our study. The index date was defined as the date of BP > 130/80 mmHg with the adjustment of antihypertensive drugs (including the adjustment of dose and number of antihypertensive regimens). We used this definition for baseline as this would be a signal that the clinicians were trying to adjust and monitor their treatment plan to achieve the potential optimal BP target.*<br>*2. For the assignment of the treatment strategy, the definition referred to the SPRINT trial (1). In the SPRINT trial (1), the clinicians would add therapy not already in use if the patients' BP readings were over 120 mmHg in the intensive treatment group. The treatment would step down the treatment if the patients' BP readings were below 130 mmHg at this visit or below 135 mmHg on 2 consecutive visits. We used similar definition for the treatment strategy in our study since we didn't have the clinical notes for the exact treatment target in the dataset we used. In our study, we assumed that patients with BP readings of 130-140 mmHg and escalation of antihypertensive regimens were treated to reach the target of less than 130/80 mmHg. If the patients with BP readings less than 130/80 mmHg but*

	the clinicians did not step down the treatment, the patients were likely to be treated with the treatment target of <130/80 mmHg.<br><br>*To reflect clinical practice of withholding drug treatment or adjustment until the blood pressure has persistently exceeded the threshold, we used two consecutive high BP readings as the criterion for identifying patients who had adjustments in antihypertensive treatment.*<br><br>*Moreover, a similar inclusion criterion was used in our previous observational study on optimal SBP target. (2)*<br><br>*We have revised the Method part on the manuscript to further clarify.*<br><br>*Reference:*<br>*(1) SPRINT Research Group. (2015). A randomized trial of intensive versus standard blood-pressure control. New England Journal of Medicine, 373(22), 2103-2116.*<br>*(2) Xu, W., Goldberg, S. I., Shubina, M., & Turchin, A. (2015). Optimal systolic blood pressure target, time to intensification, and time to follow-up in treatment of hypertension: population based retrospective cohort study. BMJ, 350.*

R1(8):	Could you explain better why approximately 83000 patients were assigned to the traditional target and approximately 16000 patients were assigned to the intensive target?
A:	*Thank you very much for your comment. We would like to clarify that the actual number of patients was identified from the HA database based on those who satisfied the inclusion criteria for the specific group. The details of the inclusion criteria and the definition of the specific group are summarized in our response to R1(7).*

R1(9):	You state: "The crude incidence rate for each outcome is summarized inTable S3." Please check.
A:	*Thank you very much for your comment. We apologize the wrong number of the table S3. The crude incidence rate for each outcome was summarized in Table 2. We have revised it in the manuscript:*<br>*The crude incidence rate for each outcome is summarized in Table 2. (Page 7, Lines 128-129)*

R1(10):	Lines 122-124: "Over 60% of the patients with intensive blood pressure target adhered to the treatment strategy while 20% of the patients with traditional treatment target did not achieve or considered a lower blood pressure target (Figure S2)." This sentence is unclear to me.
A:	*Thank you very much for your comments. We apologize the unclear description. We have rephrased this sentence in the manuscript to improve clarity:*

	Adherence was around 60% for patients in intensive treatment strategy versus 80% in traditional treatment strategy (Supplementary Figure 2). (Page 7, Lines129-130)

R1(11):	Please check the title of Figure 2. Also, this figure appears to be a duplicate of Table 2.
A:	*Thank you for your comment. We have revised the title for Figure 2 and reformatted Table 2 to avoid duplication of contents reported in the manuscript.*

R1(12):	I cannot find Figure 3 in the text.
A:	*Thank you very much for your comment. We have inserted the Figure in the manuscript as below:*<br>*The cumulative incidence rate of major CVD and all-cause mortality was summarized in the Figure 3. (Page 8, Lines 141-142)*

R1(13):	Supplementary Table 8: Please comment on the results on CHD risk according to age ranges.<br>Refs 9 and 17 are the same.<br>Refs 13 and 21 are the same.
A:	*Thank you for the comment. We have included description of the findings on the risk of CHD associated with different blood pressure monitoring target reported in Supplementary Table 8 in the main text of the manuscript to guide the interpretation of the results:*<br>*The risk reduction effects in CHD associated with intensive blood pressure monitoring target was more profound among patients aged between 65 and 80 compared to patients from other age-subgroups. (Page 8, Lines 145-147)*<br><br>*We have also checked for and removed any duplicated references in the manuscript.*

R1(14):	At least for me, it is difficult to follow some parts of the discussion section which should be overall shortened and focused on the main findings.<br>There are some typos.<br>English language should be improved.
A:	*Thank you for the comment. We have tightened the discussion section to focus on the main findings of this study. We have also carefully proof-read the manuscript to ensure the English is correct.*

Reviewer 2

R2:	I appreciate the opportunity to review the manuscript “Optimal blood pressure target for antihypertensive management in patients with uncomplicated hypertension: Real-world evidence from a target trial emulation study”. The authors conducted a target trial emulation to evaluate the benefits of the cardiovascular diseases risk reduction in hypertension patients managed by

	intensive compared to standard BP target using real-world electronic health records in Hong Kong. Overall, the manuscript is well written. However, there are some points that require work to improve the clearness and readability. Please find below some questions and suggestions:
A:	*Thank you for your encouraging comment and helpful suggestions. Please see our detailed response to your separate comments below.*

R2(1):	The authors defined intensive treatment as two consecutive records of BP <130/80 mmHg without de-escalation or BP 130-140/ 80-90 mmHg with escalation of drugs treatment. This seems to be arbitrary. I would suggest providing the rationale of using this definition and adding some sensitivity analyses.
A:	*Thank you for your comment and helpful suggestions. We would like to explain that the target blood pressure definitions and inclusion criteria in our study were adapted from those of the SPRINT trial (1). Further details regarding the definition of treatment strategy are provided in our response to comment number R1 (7).*<br><br>*Based on the clinical expertise of our co-investigators and in line with common clinical practices, most clinicians tend to avoid adjusting hypertensive medication prescriptions based on a single elevated BP reading during a consultation, unless the serious medication side effects are present. This approach is supported by the understanding that BP can fluctuate due to factors such as stress, anxiety, or white coat hypertension, and overreacting to a single reading may lead to unnecessary treatment changes (2). Instead, clinicians typically opt to monitor BP readings over time to determine whether the elevated levels persist, as sustained high BP is a more reliable indicator of uncontrolled hypertension. If high BP is consistently observed, clinicians are more likely to adjust the dosage, add another antihypertensive medication, or switch to a different drug class to achieve better control. This practice aligns with evidence-based guidelines from organizations like the American Heart Association (3) and the European Society of Hypertension (4), which emphasize the importance of confirming hypertension through multiple readings before initiating or intensifying treatment. To reflect clinical practice of withholding drug treatment or adjustment until the blood pressure has persistently exceeded the threshold, we used two consecutive high BP readings as the criterion for identifying adjustments in antihypertensive treatment. And the 12-month grace period was needed for clinicians to monitor for the BP target following the antihypertensive treatment.*<br><br>*Moreover, similar inclusion criteria for treatment group assignment were used in our previous observational study investigating the optimal SBP target (5)*<br><br>*However, we acknowledge that our study inclusion criteria might have limitations since we could not validate them by the clinical notes. Per your suggestion, we conducted three sensitivity analyses: 1. using 6-month instead of 1-year as grace period; 2. Assigning the desired BP target based on a single BP measurement and concurrent antihypertensive drugs record; 3. Assigning the desired BP target based on three consecutive BP measurements and concurrent antihypertensive drugs records. The results are summarized below, which were consistent with the results from the main analysis.*

Supplementary Table 13 Hazard ratios estimates of different blood pressure treatment target on patients (using 6-month as grace period)

**Outcome**	**Adjusted hazard ratio (95% CI)**	**P value**
Major CVD	0.80 (0.73-0.88)	<0.01
CHD	0.71 (0.62-0.82)	<0.01
Heart Failure	0.88 (0.70-1.11)	0.27
Stroke	0.83 (0.73-0.95)	0.01
ESRD	0.84 (0.62-1.12)	0.23
Mortality	0.84 (0.73-0.98)	0.03
Serious adverse event		
Composite of seven serious adverse event	0.93 (0.87-1.00)	0.07
Hypotension	0.88 (0.62-1.27)	0.50
Syncope	1.02 (0.85-1.22)	0.84
Bradycardia	0.44 (0.15-1.26)	0.13
Electrolyte abnormality	1.80 (0.22-14.94)	0.59
Falls	0.90 (0.81-1.01)	0.07
Acute kidney disease	0.79 (0.56-1.10)	0.16
Dizziness	0.98 (0.87-1.10)	0.74

Note: Major CVD: composite outcomes of heart failure, chronic heart disease and stroke; CHD: chronic heart disease; ESRD: end-stage renal disease; Analyses adjusted for sex, age, smoking status, fasting glucose, high-density lipoprotein cholesterol, low-density lipoprotein cholesterol, triglyceride, total cholesterol, eGFR, Charlson Comorbidities Index, usage of ACEI/ARB, β-blocker, calcium channel blockers, diuretic; history of fall, syncope and electrolyte abnormality, Specialist, General Outpatient Clinics attendance, accident and emergency and hospitalization (within 1 year before baseline).

Supplementary Table 14 Hazard ratios estimates of different blood pressure treatment target on patients (using single record for defining treatment strategy)

**Outcome**	**Adjusted hazard ratio (95% CI)**	**P value**
Major CVD	0.83 (0.77-0.90)	<0.01
CHD	0.85 (0.76-0.95)	<0.01
Heart Failure	1.10 (0.90-1.35)	0.36
Stroke	0.81 (0.72-0.90)	<0.01
ESRD	0.81 (0.62-1.05)	0.11
Mortality	0.88 (0.79-0.99)	0.03
Serious adverse event		
Composite of seven serious adverse event	1.01 (0.96-1.07)	0.72
Hypotension	1.05 (0.82-1.34)	0.71
Syncope	0.98 (0.86-1.11)	0.75
Bradycardia	0.58 (0.33-1.01)	0.05
Electrolyte abnormality	0.84 (0.20-3.56)	0.82
Falls	0.98 (0.91-1.06)	0.62
Acute kidney disease	0.84 (0.20-3.56)	0.82
Dizziness	1.02 (0.94-1.11)	0.58

Note: Major CVD: composite outcomes of heart failure, chronic heart disease and stroke; CHD: chronic heart disease; ESRD: end-stage renal disease; Analyses adjusted for sex, age, smoking status, fasting glucose, high-density lipoprotein cholesterol, low-density lipoprotein cholesterol, triglyceride, total cholesterol, eGFR, Charlson Comorbidities Index, usage of ACEI/ARB, β-blocker, calcium channel blockers, diuretic; history of fall, syncope and electrolyte abnormality, Specialist, General Outpatient Clinics attendance, accident and emergency and hospitalization (within 1 year before baseline).

Supplementary Table 15 Hazard ratios estimates of different blood pressure treatment target on patients (using 3 consecutive records for defining treatment strategy)

**Outcome**	**Adjusted hazard ratio (95% CI)**	**P value**
Major CVD	0.78 (0.71-0.87)	<0.01
CHD	0.69 (0.59-0.81)	<0.01
Heart Failure	0.89 (0.69-1.15)	0.38
Stroke	0.86 (0.74-0.99)	0.04
ESRD	0.73 (0.54-0.98)	0.03
Mortality	0.79 (0.68-0.92)	<0.01

Serious adverse event		
Composite of seven serious adverse event	0.96 (0.89-1.03)	0.26
Hypotension	0.58 (0.37-0.92)	0.02
Syncope	0.89 (0.73-1.08)	0.24
Bradycardia	0.58 (0.19-1.74)	0.33
Electrolyte abnormality	1.90 (0.23-16.04)	0.56
Falls	0.95 (0.85-1.07)	0.40
Acute kidney disease	0.67 (0.45-1.00)	0.05
Dizziness	1.05 (0.93-1.18)	0.45

Note: Major CVD: composite outcomes of heart failure, chronic heart disease and stroke; CHD: chronic heart disease; ESRD: end-stage renal disease; Analyses adjusted for sex, age, smoking status, fasting glucose, high-density lipoprotein cholesterol, low-density lipoprotein cholesterol, triglyceride, total cholesterol, eGFR, Charlson Comorbidities Index, usage of ACEI/ARB, β-blocker, calcium channel blockers, diuretic; history of fall, syncope and electrolyte abnormality, Specialist, General Outpatient Clinics attendance, accident and emergency and hospitalization (within 1 year before baseline).

Besides, we have also pointed out this limitation in the Discussion section of the revised manuscript as follows:

Firstly, the information of the desired blood pressure target was not recorded in the CMS database. We assumed the blood pressure target based on the blood pressure and the concurrent antihypertensive drugs prescription data, which might introduce a misclassification of the treatment target groups. (Page 11, Lines 214-217)

Nevertheless, the results of the sensitivity analysis with a short grace period of six-months and the sensitivity analysis using different numbers of consecutive records of BP readings concurrent with the prescription records have yielded consistent findings, showing that any potential misclassification bias using this definition should not have a major impact on the overall findings. (Page 12, Lines 221-225)

Reference:

(1) *SPRINT Research Group. (2015). A randomized trial of intensive versus standard blood-pressure control. New England Journal of Medicine, 373(22), 2103-2116.*
(2) *Cobos B, Haskard-Zolnierek K, Howard K. White coat hypertension: improving the patient-health care practitioner relationship. Psychol Res Behav Manag. 2015 May 2;8:133-41.*
(3) *Whelton PK, et al. 2017 ACC/AHA/AAPA/ABC/ACPM/AGS/APhA/ASH/ASPC/NMA/PCNA Guideline for the Prevention, Detection, Evaluation, and Management of High Blood Pressure in Adults: Executive Summary: A Report of the American College of Cardiology/American Heart Association Task Force on Clinical Practice Guidelines. Hypertension 71, 1269-1324 (2018).*
(4) *Mancia G, et al. 2023 ESH Guidelines for the management of arterial hypertension The Task Force for the management of arterial hypertension of the European Society of Hypertension: Endorsed by the International Society of Hypertension (ISH) and the European Renal Association (ERA). Journal of Hypertension 41, 1874-2071 (2023).*
(5) *Xu, W., Goldberg, S. I., Shubina, M., & Turchin, A. (2015). Optimal systolic blood pressure target, time to intensification, and time to follow-up in treatment of hypertension: population based retrospective cohort study. BMJ, 350.*

R2(2): The outcomes were obtained based on coding of disease diagnoses. The adverse events were defined as the diagnosis that required hospital admission. This may underestimate the risk of adverse outcomes, as patients with some adverse events, such as dizziness may not be hospitalized or be reported as a discharge diagnosis.

A: *Thank you very much for your comments. Our study focused on serious adverse or life-threatening events requiring hospital admission as examined in SPRINT trial (1). However, we understand your concern that the hospital admission data could underestimate the risk of adverse outcomes. Therefore, we conducted a sensitivity analysis that included diagnoses from both outpatient and inpatient settings. The results for this analysis shown below were largely consistent with the main analysis, except for a slightly higher and statistically significant risk of dizziness when outpatient diagnoses were included.*

Supplementary Table 16 Hazard ratio estimates for safety outcomes of different blood pressure treatment target on patients (incidence of adverse events from both inpatient and outpatient settings)

Outcome	Adjusted hazard ratio (95% CI)	P value
Serious adverse event		
Composite of seven serious adverse event	0.99 (0.97-1.02)	0.55
Hypotension	0.96 (0.70-1.33)	0.82
Syncope	1.06 (1.01-1.11)	0.03
Bradycardia	0.96 (0.54-1.68)	0.88
Electrolyte abnormality	0.97 (0.94-1.01)	0.11
Falls	0.98 (0.91-1.07)	0.70
Acute kidney disease	0.85 (0.67-1.08)	0.18
Dizziness	1.05 (1.00-1.10)	0.05

Note: Analyses adjusted for sex, age, smoking status, fasting glucose, high-density lipoprotein cholesterol, low-density lipoprotein cholesterol, triglyceride, total cholesterol, eGFR, Charlson Comorbidities Index, usage of ACEI/ARB, β-blocker, calcium channel blockers, diuretic; history of fall, syncope and electrolyte abnormality, Specialist, General Outpatient Clinics attendance, accident and emergency and hospitalization (within 1 year before baseline).

Reference:

(1) *SPRINT Research Group. (2015). A randomized trial of intensive versus standard blood-pressure control. New England Journal of Medicine, 373(22), 2103-2116.*

R2(3): A negative control outcome, for example, cancer incidence would be considered. The conclusion of this study would be strengthened, if no association was found between BP lowering treatments and cancer incidence.

A: *Thank you very much for your advice. As you suggested, we conducted a sensitivity analysis using cancer as a negative control. We did not observe any significant difference in the risk of cancer between the two groups.*

Supplementary Table 17 Hazard ratios estimates for safety outcomes of different blood pressure treatment target on patients (using cancer as a negative control outcome)

	**Outcome** / **Adjusted hazard ratio (95% CI)** / **P value**<br>Cancer / 1.03 (0.97-1.10) / 0.23<br>Note: Analyses adjusted for sex, age, smoking status, fasting glucose, high-density lipoprotein cholesterol, low-density lipoprotein cholesterol, triglyceride, total cholesterol, eGFR, Charlson Comorbidities Index, usage of ACEI/ARB, β-blocker, calcium channel blockers, diuretic; history of fall, syncope and electrolyte abnormality, Specialist, General Outpatient Clinics attendance, accident and emergency and hospitalization (within 1 year before baseline).

R2(4):	It is not clear for the choice of variables in the inverse probability weighting. It seems that a few major potential confounders are not included. For example, the baseline BP levels, duration of hypertension diagnosis, alcohol consumption, diet, and physical activity.
A:	*Thank you very much for your insightful comments. We fully understand your concerns regarding the choice of variables in the inverse probability weighting. Therefore, we have conducted a sensitivity analysis including baseline BP levels in the weighting model. The results of the analysis presented below were consistent with the results from the main analysis.*

Supplementary Table 18 Hazard ratios estimates of different blood pressure treatment target on patients (Including baseline BP readings in the weighting model)

Outcome	Adjusted hazard ratio (95% CI)	P value
Major CVD	0.86 (0.80-0.92)	<0.01
CHD	0.81 (0.72-0.90)	<0.01
Heart Failure	0.90 (0.81-1.00)	0.06
Stroke	0.85 (0.70-1.02)	0.09
ESRD	0.81 (0.66-0.99)	0.04
Mortality	0.90 (0.82-0.99)	0.04
Serious adverse event		
Composite of seven serious adverse event	0.99 (0.93-1.04)	0.66
Hypotension	1.01 (0.77-1.32)	0.96
Syncope	0.98 (0.85-1.13)	0.80
Bradycardia	0.92 (0.53-1.62)	0.78
Electrolyte abnormality	1.59 (0.36-7.05)	0.54
Falls	1.01 (0.93-1.10)	0.81
Acute kidney disease	0.86 (0.68-1.10)	0.23
Dizziness	1.00 (0.91-1.09)	0.96

Note: Major CVD: composite outcomes of heart failure, chronic heart disease and stroke; CHD: chronic heart disease; ESRD: end-stage renal disease; Analyses adjusted for sex, age, smoking status, fasting glucose, high-density lipoprotein cholesterol, low-density lipoprotein cholesterol, triglyceride, total cholesterol, eGFR, Charlson Comorbidities Index, usage of ACEI/ARB, β-blocker, calcium channel blockers, diuretic; history of fall, syncope and electrolyte abnormality, Specialist, General Outpatient Clinics attendance, accident and emergency and hospitalization (within 1 year before baseline).

However, we could not include confounders such as alcohol consumption and physical activity in the weighting because the public electronic health records did not include data on lifestyle variables such as alcohol consumption and physical activity, which is a limitation common to other electronic health record databases.

We could not include the duration of hypertension as a confounder because the information was uncertain. Although the electronic medical record dataset could be dated back to 2006, some patients could have been diagnosed with hypertension prior to that year.

	We have pointed out the limitations from the lack of information on potential confounders in the manuscript as below: Thirdly, some potential confounders such as hypertension duration, lifestyle factors including diet and physical activity socioeconomic status and educational level are unavailable in our study, which may have introduced bias to our results. (Page 12, Lines 228-230).

R2(5):	The authors might consider deaths from non-cardiovascular events competing risks to obtain the effects of BP-lowering interventions.
A:	*Thank you very much for your comments. We have added a sensitivity analysis to consider the potential bias from the competing event of the deaths from non-cardiovascular events. The competing risk adjustment was performed in the per-protocol analysis, in which each clone additionally received a time-varying inverse probability weight of not dying of non-cardiovascular events. The results are shown below, which were consistent with those of the main analysis.*

Supplementary Table 19 Hazard ratios estimates of different blood pressure treatment target on patients (adjusting for competing risk)

Outcome	Adjusted hazard ratio (95% CI)	P value
Major CVD	0.90 (0.83-0.98)	0.02
CHD	0.85 (0.74-0.97)	0.02
Heart Failure	0.98 (0.87-1.10)	0.71
Stroke	0.98 (0.78-1.22)	0.83
ESRD	0.90 (0.70-1.15)	0.38
Serious adverse event		
Composite of seven serious adverse event	0.99 (0.94-1.05)	0.77
Hypotension	1.00 (0.77-1.31)	0.99
Syncope	1.00 (0.87-1.15)	0.99
Bradycardia	0.94 (0.53-1.64)	0.82
Electrolyte abnormality	1.43 (0.34-6.08)	0.63
Falls	1.01 (0.93-1.09)	0.89
Acute kidney disease	0.86 (0.68-1.09)	0.21
Dizziness	1.01 (0.93-1.11)	0.80

Note: Major CVD: composite outcomes of heart failure, chronic heart disease and stroke; CHD: chronic heart disease; ESRD: end-stage renal disease; Analyses adjusted for sex, age, smoking status, fasting glucose, high-density lipoprotein cholesterol, low-density lipoprotein cholesterol, triglyceride, total cholesterol, eGFR, Charlson Comorbidities Index, usage of ACEI/ARB, β-blocker, calcium channel blockers, diuretic; history of fall, syncope and electrolyte abnormality, Specialist, General Outpatient Clinics attendance, accident and emergency and hospitalization (within 1 year before baseline).

R2(6):	Individuals with a prescription for aspirin on or before baseline were excluded to avoid the under-diagnosis of CVD history. However, aspirin is still widely used in primary prevention during 2008 to 2013.
A:	*Thank you very much for your comment. Although aspirin was widely used for primary prevention in some countries, such as the U.S. from 2008 to 2013 (1), it had never been recommended for primary prevention of CVD in Hong Kong (2).*<br><br>*For the population included in our study, the likelihood of aspirin use for primary prevention should be low, given the exclusion of subjects with a history of cardiovascular disease, diabetes, or chronic kidney disease. It is possible that patients prescribed aspirin may have had a diagnosis of CVD. To better assess the risk of first cardiovascular events, we have excluded patients with aspirin used to*

ensure patients without CVD to evaluate the effect of intensive blood pressure target for primary prevention.

However, we fully understand your concern regarding the number of patients with an aspirin prescription at or before baseline. Therefore, we conducted a sensitivity analysis including patients on aspirin. The results summarized below were largely consistent with those of the main analysis.

Supplementary Table 20 Hazard ratios estimates of different blood pressure treatment target on patients (Including patients with low-dose aspirin prescriptions on or before baseline)

Outcome	Adjusted hazard ratio (95% CI)	P value
Major CVD	0.93 (0.87-0.99)	0.02
CHD	0.91 (0.83-0.99)	0.04
Heart Failure	0.85 (0.72-1.01)	0.07
Stroke	0.97 (0.89-1.06)	0.53
ESRD	0.81 (0.67-0.99)	0.04
Mortality	0.89 (0.81-0.98)	0.02
Serious adverse event		
Composite of seven serious adverse event	0.99 (0.94-1.04)	0.73
Hypotension	1.01 (0.79-1.29)	0.93
Syncope	1.01 (0.88-1.15)	0.92
Bradycardia	1.02 (0.64-1.63)	0.95
Electrolyte abnormality	1.41 (0.33-5.99)	0.64
Falls	1.00 (0.92-1.08)	0.94
Acute kidney disease	0.88 (0.71-1.10)	0.27
Dizziness	1.01 (0.93-1.10)	0.78

Note: Major CVD: composite outcomes of heart failure, chronic heart disease and stroke; CHD: chronic heart disease; ESRD: end-stage renal disease; Analyses adjusted for sex, age, smoking status, fasting glucose, high-density lipoprotein cholesterol, low-density lipoprotein cholesterol, triglyceride, total cholesterol, eGFR, Charlson Comorbidities Index, usage of ACEI/ARB, β-blocker, calcium channel blockers, diuretic; history of fall, syncope and electrolyte abnormality, Specialist, General Outpatient Clinics attendance, accident and emergency and hospitalization (within 1 year before baseline).

Reference:

(1) *Huang AL, Navar AM, Ayers C, Rohatgi A, Michos ED, Virani SS, Joshi P, Peterson ED, Khera A. US population qualifying for aspirin use for primary prevention of cardiovascular disease. Am J Prev Cardiol. 2024 Apr 16;18:100669. doi: 10.1016/j.ajpc.2024.100669. PMID: 38681065; PMCID: PMC11046250.*

(2) *Drug Office, Department of Health, Hong Kong SAR. https://www.drugoffice.gov.hk/eps/do/en/consumer/news_informations/dm_03.html*

Reviewer 3

R3:	In this manuscript, Zhang et al. report the results of a target trial emulation study using real-world electronic health record data to evaluate optimal blood pressure targets for patients with uncomplicated hypertension. The hypothesis is clearly stated, and the manuscript is organized and overall well-written. The authors showed that the group assigned to intensive blood pressure target had significantly

	lower risk of cardiovascular events and mortality compared to those assigned to the standard target group over 7 years of follow-up.<br><br>I agree with two of the strengths espoused by the authors: 1) the use of target trial emulation methodology, which provides a rigorous framework for analyzing observational data to approximate a randomized controlled trial; and 2) the large sample size (98,745 patients) with extended follow-up, which provides robust real-world evidence. The authors further employed sensitivity analyses and subgroup analyses to strengthen the findings.<br>There are several major concerns noted below:
A:	*Thank you for your encouraging comment and helpful suggestions. Please see our detailed response to your separate comments below.*

R3(1):	I have several queries regarding treatment assignment. Time zero is defined as the "first date of prescription adjustment," and subjects are given a 12-month grace period to determine eligibility. It appears that treatment strategies were defined retrospectively based on observed clinician prescriptions and BP trends, rather than according to a pre-specified protocol. This suggests a post-treatment classification, where the treatment group is inferred from downstream outcomes (i.e., BP trends and medication escalation). This approach raises concerns about potential reverse causation, where BP readings influence medication decisions, yet are treated as independent indicators of the assigned strategy. Additionally, it appears that all subjects with BP >140/90 mmHg are automatically considered to be on the "standard" treatment strategy. Given that this is an EHR-based study, it may be possible to incorporate clinical notes documenting physicians' explicitly stated BP targets to classify patients based on intended treatment strategies rather than observed patterns alone.
A:	*Thank you very much for your comments and suggestions. We agree that a review of clinical notes would provide more accurate information on the desired BP treatment target of individual patients, but such data could not be extracted from the electronic medical records because of confidentiality issues. We acknowledge your concern on post-treatment classification and reverse causation. We believe the target trial emulation with cloning and censor approach could have reduced the bias (1,2). We would also like to further explain the inclusion eligibility and treatment group assignment criteria.*<br><br>*The time-zero of this study refers to the index date defined as the date of BP > 130/80 with the adjustment of antihypertensive drugs (including the adjustment of dose and number of antihypertensive regimens) under the assumption that these changes attempted to achieve the desired BP target. The BP readings and prescriptions were therefore not considered as an indication for treatment strategy at this stage as their desired BP target has not been determined yet. Since the treatment strategy of individuals should not be ascertained based on baseline information, a 12-month grace period was implemented.*<br><br>*The grace period allowed us to observe for any changes in patient's BP from the baseline measurement and adjustment in treatment to achieve the desired target, thus enabled us to accurately deduce the treatment strategy. Besides, it ensures that the strategies remain realistic and increases the number of people in the*

observational database whose data can be used to emulate the target trial (1). A similar design in the proportion of the grace period to the total follow-up time was used in other studies using clone-censor-weight approach (2,3). We understand 12-months grace period may appear to be long and requirement of two consecutive records may artificially censor some unstable patients, so we carried out a sensitivity analysis using 6-month instead of 12-month as grace period. The table below summarized the results which were consistent with the main analysis.*

Supplementary Table 13 Hazard ratios estimates of different blood pressure treatment target on patients (using 6-month as grace period)

Outcome	Adjusted hazard ratio (95% CI)	P value
Major CVD	0.80 (0.73-0.88)	<0.01
CHD	0.71 (0.62-0.82)	<0.01
Heart Failure	0.88 (0.70-1.11)	0.27
Stroke	0.83 (0.73-0.95)	0.01
ESRD	0.84 (0.62-1.12)	0.23
Mortality	0.84 (0.73-0.98)	0.03
Serious adverse event		
Composite of seven serious adverse event	0.93 (0.87-1.00)	0.07
Hypotension	0.88 (0.62-1.27)	0.50
Syncope	1.02 (0.85-1.22)	0.84
Bradycardia	0.44 (0.15-1.26)	0.13
Electrolyte abnormality	1.80 (0.22-14.94)	0.59
Falls	0.90 (0.81-1.01)	0.07
Acute kidney disease	0.79 (0.56-1.10)	0.16
Dizziness	0.98 (0.87-1.10)	0.74

Note: Major CVD: composite outcomes of heart failure, chronic heart disease and stroke; CHD: chronic heart disease; ESRD: end-stage renal disease; Analyses adjusted for sex, age, smoking status, fasting glucose, high-density lipoprotein cholesterol, low-density lipoprotein cholesterol, triglyceride, total cholesterol, eGFR, Charlson Comorbidities Index, usage of ACEI/ARB, β-blocker, calcium channel blockers, diuretic; history of fall, syncope and electrolyte abnormality, Specialist, General Outpatient Clinics attendance, accident and emergency and hospitalization (within 1 year before baseline).

Further details regarding the assignment of treatment strategy are provided in our response to comment number R1 (7).

Besides, we would like to explain that we censored all subjects with blood pressure readings exceeding 140/90 mmHg at the end of the grace period because they did not meet the standard treatment goal and we could not determine their desired BP target. For example, when a patient escalated treatment with a blood pressure of 145/91 mmHg, it is possible that this patient was targeting either the standard goal or a more intensive target of <130/80 mmHg. For patients who did not explicitly consider the intensive treatment target, we assumed they were following the standard treatment goal as recommended by the Hong Kong treatment guidelines (4).

We have discussed the above limitations as below in our revised manuscript:
Firstly, the information of the desired blood pressure target was not recorded in the CMS database. We assumed the blood pressure target based on the blood pressure and the concurrent antihypertensive drugs prescription data, which might introduce a misclassification of the treatment target groups. Besides, the treatment assignment after the long grace period might potentially introduce further misclassification bias. However, given the prolonged follow-up in this study of over 7 years, the duration of grace period included in relation to the total follow-up period was in-line with previous study employing the clone-censor-weight design. Nevertheless, the results of the sensitivity analysis with a short grace

	period of six-months and the sensitivity analysis using different numbers of consecutive records of BP readings concurrent with the prescription records have yielded consistent findings, showing that any potential misclassification bias using this definition should not have a major impact on the overall findings. (Pages 11-12, Lines 215-225)<br><br>*Besides, we have revised the Methods section to improve clarity in our revised manuscript.*<br><br>*Reference:*<br>*(1) Hernán MA, Robins JM. Using Big Data to Emulate a Target Trial When a Randomized Trial Is Not Available. Am J Epidemiol. 2016;183(8):758-64.*<br>*(2) Chen, A. et al. Impact of beta-blockers on mortality and cardiovascular disease outcomes in patients with obstructive sleep apnoea: a population-based cohort study in target trial emulation framework. The Lancet Regional Health – Europe, 33, 100715. (2023)*<br>*(3) Trevisan M, et al. Stopping mineralocorticoid receptor antagonists after hyperkalaemia: trial emulation in data from routine care. Eur J Heart Fail. 2021;23(10):1698-1707.*<br>*(4) Hong Kong Special Administrative Region Government. (2013). Guidelines on the management of hypertension in adults. Department of Health, Hong Kong SAR.*

R3(2):	Enrollment in this target emulation trial began in 2008, well before publication of major randomized controlled trials (e.g., SPRINT, PBROAD, ESPRIT) showing lower BP was beneficial. How do the authors account for the rationale for lower BP in the intensive group? What was the distribution of enrollment dates in both groups by year? This could introduce major confounding as recommendations for SBP < 130 mmHg were not guideline based at the time of observation. Of course, widespread adoption of guidelines after publication are slow, but least there was a rationale.
A:	*Thank you very much for your comments. The table below summarized the patients managed in traditional and lower BP targets before and after 2010. As shown in the table, the percentage of people in intensive treatment and standard treatment arms are consistent before and after the publication of the major RCTs. Therefore, confounding by time of recruitment should not have a major influence.*

**Baseline (year)**	**SBP <140mmHg and DBP <90 mmHg**	**SBP<130mmHg and DBP<80mmHg**
2008	5,796 (82.5%)	1,231 (17.5%)
2009	8,998 (84.0%)	1,711 (16.0%)
2010	14,545 (84.1%)	2,757 (15.9%)
2011	16,807 (84.2%)	3,159 (15.8%)
2012	17,772 (84.1%)	3,350 (15.9%)

	However, as we cannot exclude the possibility of time-varying confounder, we have conducted a sensitivity analysis adjusting the year of the enrolment in the weighting model. The results from this analysis are summarized below, which yield consistent results as the main analysis.<br><br>**Supplementary Table 20 Hazard ratios estimates of different blood pressure treatment target on patients (Including the year of the enrolment in the weighting model)**

Outcome	Adjusted hazard ratio (95% CI)	P value
Major CVD	0.80 (0.73-0.88)	<0.01
CHD	0.71 (0.62-0.82)	<0.01
Heart Failure	0.88 (0.70-1.11)	0.27
Stroke	0.89 (0.80-0.99)	0.03
ESRD	0.84 (0.62-1.12)	0.23
Mortality	0.88 (0.80-0.97)	0.01
Serious adverse event		
Composite of seven serious adverse event	0.99 (0.93-1.04)	0.65
Hypotension	1.01 (0.77-1.32)	0.94
Syncope	0.99 (0.86-1.14)	0.88
Bradycardia	0.93 (0.53-1.63)	0.80
Electrolyte abnormality	1.51 (0.34-6.70)	0.59
Falls	1.01 (0.93-1.10)	0.83
Acute kidney disease	0.86 (0.68-1.09)	0.22
Dizziness	1.00 (0.91-1.09)	0.98

Note: Major CVD: composite outcomes of heart failure, chronic heart disease and stroke; CHD: chronic heart disease; ESRD: end-stage renal disease; Analyses adjusted for sex, age, smoking status, fasting glucose, high-density lipoprotein cholesterol, low-density lipoprotein cholesterol, triglyceride, total cholesterol, eGFR, Charlson Comorbidities Index, usage of ACEI/ARB, β-blocker, calcium channel blockers, diuretic; history of fall, syncope and electrolyte abnormality, Specialist, General Outpatient Clinics attendance, accident and emergency and hospitalization (within 1 year before baseline).

R3(3):	The 12-month grace period may introduce bias. While the authors attempt to account for immortal time bias using cloning and censoring, the bias may still persist. Subjects must survive the entire 12-month grace period to be eligible, which could exclude those who experience clinical outcomes during this period. Moreover, only patients with consistent follow-up data—specifically, two consecutive BP measurements—are eligible, though the interval between these measurements is not clearly defined. This could lead to survivorship bias, where only patients actively engaged in their healthcare remain in the cohort, while sicker patients may have "dropped out" during the grace period.
A:	*Thank you very much for your comments. We would like to explain that although the grace period is 12 -month, this does not mean that all patients must survive the entire 12 months. We have clarified the inclusion criteria we used in our study in R3(1). Due to the design of the clone-censor-weight approach, any events occurring during the grace period were counted in the clones from both treatment arms to avoid immortal time bias (1,2).*<br><br>*We acknowledge your concern regarding survivorship bias in our study. We would like to explain that patients who did not have two consecutive records were censored instead of being dropped out. At the end of the grace period, we conducted the inverse probability weighting to adjust for any potential bias from the artificial censoring of patient. Additionally, according to the recommendation*

from the treatment guidelines, patients with hypertension typically have doctor consultations every 3 months, with an annual assessment after the diagnosis of hypertension. Therefore, the majority of patients should have at least two records during the grace period (3). We calculated the number of BP readings during the grace period and found that the proportion of patient having single BP record is 0.22%, indicating that the probability of censoring during the grace period due to inattentiveness to their healthcare remains low. Furthermore, given the concern on the survivorship bias, we conducted a sensitivity analysis using single record to indicate the treatment assignment. The results for this analysis are shown below, which were similar to the results from the main analysis.

Supplementary Table 14 Hazard ratios estimates of different blood pressure treatment target on patients (using single record for defining treatment strategy)

Outcome	Adjusted hazard ratio (95% CI)	P value
Major CVD	0.83 (0.77-0.90)	<0.01
CHD	0.85 (0.76-0.95)	<0.01
Heart Failure	1.10 (0.90-1.35)	0.36
Stroke	0.81 (0.72-0.90)	<0.01
ESRD	0.81 (0.62-1.05)	0.11
Mortality	0.88 (0.79-0.99)	0.03
Serious adverse event		
Composite of seven serious adverse event	1.01 (0.96-1.07)	0.72
Hypotension	1.05 (0.82-1.34)	0.71
Syncope	0.98 (0.86-1.11)	0.75
Bradycardia	0.58 (0.33-1.01)	0.05
Electrolyte abnormality	0.84 (0.20-3.56)	0.82
Falls	0.98 (0.91-1.06)	0.62
Acute kidney disease	0.84 (0.20-3.56)	0.82
Dizziness	1.02 (0.94-1.11)	0.58

Note: Major CVD: composite outcomes of heart failure, chronic heart disease and stroke; CHD: chronic heart disease; ESRD: end-stage renal disease; Analyses adjusted for sex, age, smoking status, fasting glucose, high-density lipoprotein cholesterol, low-density lipoprotein cholesterol, triglyceride, total cholesterol, eGFR, Charlson Comorbidities Index, usage of ACEI/ARB, β-blocker, calcium channel blockers, diuretic; history of fall, syncope and electrolyte abnormality, Specialist, General Outpatient Clinics attendance, accident and emergency and hospitalization (within 1 year before baseline).

The reasons for two consecutive records used in this study were explained in R1 (7).

However, we also conducted the sensitivity analyses by 1. using 6-month as grace period; 2. using three consecutive records to indicate the treatment assignment to check the robustness of our study outcomes.

Supplementary Table 13 Hazard ratios estimates of different blood pressure treatment target on patients (using 6-month as grace period)

Outcome	Adjusted hazard ratio (95% CI)	P value
Major CVD	0.80 (0.73-0.88)	<0.01
CHD	0.71 (0.62-0.82)	<0.01
Heart Failure	0.88 (0.70-1.11)	0.27
Stroke	0.83 (0.73-0.95)	0.01
ESRD	0.84 (0.62-1.12)	0.23
Mortality	0.84 (0.73-0.98)	0.03
Serious adverse event		
Composite of seven serious adverse event	0.93 (0.87-1.00)	0.07
Hypotension	0.88 (0.62-1.27)	0.50

Syncope	1.02 (0.85-1.22)	0.84
Bradycardia	0.44 (0.15-1.26)	0.13
Electrolyte abnormality	1.80 (0.22-14.94)	0.59
Falls	0.90 (0.81-1.01)	0.07
Acute kidney disease	0.79 (0.56-1.10)	0.16
Dizziness	0.98 (0.87-1.10)	0.74

Note: Major CVD: composite outcomes of heart failure, chronic heart disease and stroke; CHD: chronic heart disease; ESRD: end-stage renal disease; Analyses adjusted for sex, age, smoking status, fasting glucose, high-density lipoprotein cholesterol, low-density lipoprotein cholesterol, triglyceride, total cholesterol, eGFR, Charlson Comorbidities Index, usage of ACEI/ARB, β-blocker, calcium channel blockers, diuretic; history of fall, syncope and electrolyte abnormality, Specialist, General Outpatient Clinics attendance, accident and emergency and hospitalization (within 1 year before baseline).

Supplementary Table 15 Hazard ratios estimates of different blood pressure treatment target on patients (using 3 consecutive records for defining treatment strategy)

Outcome	Adjusted hazard ratio (95% CI)	P value
Major CVD	0.78 (0.71-0.87)	<0.01
CHD	0.69 (0.59-0.81)	<0.01
Heart Failure	0.89 (0.69-1.15)	0.38
Stroke	0.86 (0.74-0.99)	0.04
ESRD	0.73 (0.54-0.98)	0.03
Mortality	0.79 (0.68-0.92)	<0.01
Serious adverse event		
Composite of seven serious adverse event	0.96 (0.89-1.03)	0.26
Hypotension	0.58 (0.37-0.92)	0.02
Syncope	0.89 (0.73-1.08)	0.24
Bradycardia	0.58 (0.19-1.74)	0.33
Electrolyte abnormality	1.90 (0.23-16.04)	0.56
Falls	0.95 (0.85-1.07)	0.40
Acute kidney disease	0.67 (0.45-1.00)	0.05
Dizziness	1.05 (0.93-1.18)	0.45

Note: Major CVD: composite outcomes of heart failure, chronic heart disease and stroke; CHD: chronic heart disease; ESRD: end-stage renal disease; Analyses adjusted for sex, age, smoking status, fasting glucose, high-density lipoprotein cholesterol, low-density lipoprotein cholesterol, triglyceride, total cholesterol, eGFR, Charlson Comorbidities Index, usage of ACEI/ARB, β-blocker, calcium channel blockers, diuretic; history of fall, syncope and electrolyte abnormality, Specialist, General Outpatient Clinics attendance, accident and emergency and hospitalization (within 1 year before baseline).

Reference:

(1) Hernán MA, Robins JM. Using Big Data to Emulate a Target Trial When a Randomized Trial Is Not Available. Am J Epidemiol. 2016;183(8):758-64.

(2) Chen A, Ju C, Mackenzie IS, MacDonald TM, Struthers AD, Wei L, Man KKC. Impact of beta-blockers on mortality and cardiovascular disease outcomes in patients with obstructive sleep apnoea: a population-based cohort study in target trial emulation framework. Lancet Reg Health Eur. 2023; 33:100715.

(3) Hong Kong Special Administrative Region Government. (2013). Guidelines on the management of hypertension in adults. Department of Health, Hong Kong SAR.

R3(4): The censoring criteria seem confusing. For the standard BP target strategy clones, they are censored at month 12 if they had 5 consecutive records considering lower blood pressure target (identified as patients with BP below 140/90mmHg with prescription escalation). The rationale behind using 5 records and the specific time

	window is not clearly explained. These censoring rules are extensive and may reintroduce selection bias, undermining the very purpose of the cloning. For example, why 5 consecutive visits? Why not, e.g. 4?
A:	*Thank you very much for your comments. This criterion of 5 consecutive records was based on the usual clinical practice of following up patients with hypertension every 6 to 12 weeks and an annual assessment to monitor disease control and complication risk (1). The assessment of five consecutive records provided information on the patient's condition over approximately one year.* <br>*To address your concern, we conducted several sensitivity analyses using 2, 3, 4, and 6 consecutive records as the censoring criteria. The findings are summarized below, which were consistent with the results from the main analysis.*

Supplementary Table 21 Hazard ratios estimates of different blood pressure treatment target on patients (Using two consecutive records to define deviation from the assigned treatment strategy)

Outcome	Adjusted hazard ratio (95% CI)	P value
Major CVD	0.86 (0.78-0.94)	<0.01
CHD	0.79 (0.68-0.91)	<0.01
Heart Failure	0.85 (0.67-1.09)	0.20
Stroke	0.92 (0.81-1.05)	0.22
ESRD	0.85 (0.65-1.10)	0.22
Mortality	0.86 (0.75-0.99)	0.03
Serious adverse event		
Composite of seven serious adverse event	0.98 (0.91-1.04)	0.47
Hypotension	1.17 (0.83-1.65)	0.36
Syncope	0.99 (0.84-1.18)	0.95
Bradycardia	0.49 (0.20-1.17)	0.11
Electrolyte abnormality	0.84 (0.20-3.56)	0.82
Falls	0.98 (0.91-1.06)	0.62
Acute kidney disease	0.80 (0.59-1.08)	0.15
Dizziness	1.02 (0.92-1.13)	0.73

Note: Major CVD: composite outcomes of heart failure, chronic heart disease and stroke; CHD: chronic heart disease; ESRD: end-stage renal disease; Analyses adjusted for sex, age, smoking status, fasting glucose, high-density lipoprotein cholesterol, low-density lipoprotein cholesterol, triglyceride, total cholesterol, eGFR, Charlson Comorbidities Index, usage of ACEI/ARB, β-blocker, calcium channel blockers, diuretic; history of fall, syncope and electrolyte abnormality, Specialist, General Outpatient Clinics attendance, accident and emergency and hospitalization (within 1 year before baseline).

Supplementary Table 22 Hazard ratios estimates of different blood pressure treatment target on patients (Using three consecutive records to define deviation from the assigned treatment strategy)

Outcome	Adjusted hazard ratio (95% CI)	P value
Major CVD	0.86 (0.79-0.93)	<0.01
CHD	0.80 (0.70-0.91)	<0.01
Heart Failure	0.89 (0.72-1.10)	0.27
Stroke	0.91 (0.81-1.02)	0.11
ESRD	0.77 (0.61-0.98)	0.03
Mortality	0.87 (0.78-0.98)	0.02
Serious adverse event		
Composite of seven serious adverse event	0.98 (0.92-1.04)	0.48
Hypotension	1.09 (0.81-1.46)	0.59
Syncope	1.07 (0.92-1.24)	0.39
Bradycardia	0.51 (0.24-1.09)	0.08
Electrolyte abnormality	NA	NA
Falls	0.98 (0.90-1.07)	0.68
Acute kidney disease	0.84 (0.64-1.10)	0.21
Dizziness	1.02 (0.92-1.12)	0.74

Note: Major CVD: composite outcomes of heart failure, chronic heart disease and stroke; CHD: chronic heart disease; ESRD: end-stage renal disease; Analyses adjusted for sex, age, smoking status, fasting glucose, high-density lipoprotein cholesterol, low-density lipoprotein cholesterol, triglyceride, total cholesterol, eGFR, Charlson Comorbidities Index, usage of ACEI/ARB, β-blocker, calcium channel blockers, diuretic; history of fall, syncope and electrolyte abnormality, Specialist, General Outpatient Clinics attendance, accident and emergency and hospitalization (within 1 year before baseline).

Supplementary Table 23 Hazard ratios estimates of different blood pressure treatment target on patients (Using four consecutive records to define deviation from the assigned treatment strategy)

**Outcome**	**Adjusted hazard ratio (95% CI)**	**P value**
Major CVD	0.84 (0.78-0.91)	<0.01
CHD	0.80 (0.71-0.90)	<0.01
Heart Failure	0.86 (0.71-1.05)	0.13
Stroke	0.88 (0.79-0.98)	0.02
ESRD	0.80 (0.64-0.99)	0.04
Mortality	0.88 (0.79-0.98)	0.02
Serious adverse event		
Composite of seven serious adverse event	0.98 (0.92-1.03)	0.43
Hypotension	0.98 (0.74-1.30)	0.91
Syncope	1.02 (0.89-1.18)	0.75
Bradycardia	0.80 (0.43-1.48)	0.48
Electrolyte abnormality	1.47 (0.35-6.25)	0.60
Falls	0.99 (0.91-1.07)	0.76
Acute kidney disease	0.80 (0.62-1.03)	0.08
Dizziness	1.00 (0.92-1.10)	0.93

Note: Major CVD: composite outcomes of heart failure, chronic heart disease and stroke; CHD: chronic heart disease; ESRD: end-stage renal disease; Analyses adjusted for sex, age, smoking status, fasting glucose, high-density lipoprotein cholesterol, low-density lipoprotein cholesterol, triglyceride, total cholesterol, eGFR, Charlson Comorbidities Index, usage of ACEI/ARB, β-blocker, calcium channel blockers, diuretic; history of fall, syncope and electrolyte abnormality, Specialist, General Outpatient Clinics attendance, accident and emergency and hospitalization (within 1 year before baseline).

Supplementary Table 24 Hazard ratios estimates of different blood pressure treatment target on patients (Using six consecutive records to define deviation from the assigned treatment strategy)

**Outcome**	**Adjusted hazard ratio (95% CI)**	**P value**
Major CVD	0.86 (0.80-0.92)	<0.01
CHD	0.83 (0.74-0.92)	<0.01
Heart Failure	0.87 (0.73-1.04)	0.14
Stroke	0.90 (0.81-0.99)	0.04
ESRD	0.82 (0.68-1.00)	0.05
Mortality	0.88 (0.80-0.97)	0.01
Serious adverse event		
Composite of seven serious adverse event	1.03 (0.98-1.09)	0.26
Hypotension	1.02 (0.78-1.33)	0.90
Syncope	1.07 (0.93-1.23)	0.35
Bradycardia	1.09 (0.63-1.88)	0.77
Electrolyte abnormality	0.95 (0.22-4.09)	0.95
Falls	1.08 (0.99-1.17)	0.08
Acute kidney disease	0.91 (0.72-1.16)	0.45
Dizziness	1.06 (0.97-1.16)	0.20

Note: Major CVD: composite outcomes of heart failure, chronic heart disease and stroke; CHD: chronic heart disease; ESRD: end-stage renal disease; Analyses adjusted for sex, age, smoking status, fasting glucose, high-density lipoprotein cholesterol, low-density lipoprotein cholesterol, triglyceride, total cholesterol, eGFR, Charlson Comorbidities Index, usage of ACEI/ARB, β-blocker, calcium channel blockers, diuretic; history of fall, syncope and electrolyte abnormality, Specialist, General Outpatient Clinics attendance, accident and emergency and hospitalization (within 1 year before baseline).

Reference:

	(1) Hong Kong Special Administrative Region Government. (2013). Guidelines on the management of hypertension in adults. Department of Health, Hong Kong SAR.

R3(5):	The inclusion of medical exceptions for diabetes and CKD is appropriate, but the absence of consideration for other conditions—such as orthostatic hypotension, frailty, or medication intolerance—imposes a rigid rule-based framework that may not reflect real-world clinical practice. This limits the generalizability of the findings and may violate the positivity assumption, which requires that each participant have a non-zero probability of receiving any of the treatment strategies. For example, a frail elderly patient with hypotension might need de-escalation, but without DM or CKD, they may be censored. This implies that patients who are more susceptible to adverse effects—due to age, comorbidities, or polypharmacy—could never have feasibly received intensive treatment, even though in real practice, they might have through individualized clinical judgment. This violation has downstream implications: the treatment strategies may not represent the full covariate distribution, and inverse probability of censoring weighting (IPCW) may be biased due to extrapolation beyond the observed data, ultimately distorting per-protocol effect estimates.
A:	*Thank you very much for your comments. To address your concern that some patients may be more susceptible to adverse effects—due to age, comorbidities, or polypharmacy, we conducted a sensitivity analysis that considered patients with an increased risk of adverse effects due to advanced age (defined as older than 65 years), frailty (defined as a change in CCI greater than 2 compared to baseline), and polypharmacy (defined as taking more than three types of medicines) as contraindications. The results for this analysis are summarized below, which yielded consistent findings.*

Supplementary Table 25 Hazard ratios estimates of different blood pressure treatment target on patients (Considering side effect due to age, frailty or polypharmacy as contraindications)

Outcome	Adjusted hazard ratio (95% CI)	P value
Major CVD	0.85 (0.79-0.91)	<0.01
CHD	0.80 (0.72-0.90)	<0.01
Heart Failure	0.83 (0.69-0.99)	0.04
Stroke	0.90 (0.81-1.00)	0.05
ESRD	0.78 (0.64-0.96)	0.02
Mortality	0.88 (0.80-0.97)	0.01
Serious adverse event		
Composite of seven serious adverse event	1.03 (0.98-1.09)	0.26
Hypotension	1.02 (0.78-1.33)	0.90
Syncope	1.07 (0.93-1.23)	0.35
Bradycardia	1.09 (0.63-1.88)	0.77
Electrolyte abnormality	0.95 (0.22-4.09)	0.95
Falls	1.08 (0.99-1.17)	0.08
Acute kidney disease	0.91 (0.72-1.16)	0.45
Dizziness	1.06 (0.97-1.16)	0.20

Note: Major CVD: composite outcomes of heart failure, chronic heart disease and stroke; CHD: chronic heart disease; ESRD: end-stage renal disease; Analyses adjusted for sex, age, smoking status, fasting glucose, high-density lipoprotein cholesterol, low-density lipoprotein cholesterol, triglyceride, total cholesterol, eGFR, Charlson Comorbidities Index, usage of ACEI/ARB, β-blocker, calcium channel blockers, diuretic; history of fall, syncope and electrolyte abnormality,

	Specialist, General Outpatient Clinics attendance, accident and emergency and hospitalization (within 1 year before baseline).<br><br>*However, we also agree that we might be unable to include what all possible scenarios encountered in real-world clinical practice. We have discussed this limitation in the manuscript as follows:*<br><br>*Additionally, despite our efforts to emulate a target trial and account for various clinical scenarios, certain confounders and patients' complexities in real-world practice may not be captured as with other observational studies. Therefore, the findings reported in this study should be interpreted with caution in patients with specific clinical conditions or complexities. Future research incorporating additional real-world variables is warranted to further examine the benefits of intensive BP monitoring in other patient groups. (Page 12, Lines 230-235)*

R3(6):	Although the authors mention complete-case analysis, the extent and pattern of missing data—particularly regarding follow-up BP measurements—are not reported. It would be helpful to know how many BP measurements were available per patient and the method of BP assessment (e.g., ambulatory BP monitoring [ABPM], attended vs. unattended office readings).
A:	*Thank you very much for your comments. We apologize for any confusion regarding the number of BP assessments during follow-up and pattern of missing data during the follow-up period. In our study, the blood pressure readings were all from attended office BP readings. The number of follow-up blood pressure measurements varied among participants, with an average of 35 attended office clinic BP readings per patient during the study period.*<br><br>*In this study, a panel dataset was created for all time-varying indicators (by month) for each eligible patient. The last observation was carried forward method was used for time-varying clinical parameters during follow-up. This approach is widely employed in target trial emulation to handle time-varying exposures. (1,2) To explore the influence of carrying forward the last BP value for missing data at follow-up, we conducted a sensitivity analysis in which patients with missing BP measurements (defined as an interval of more than 1 year between two BP records) during follow-up were censored. The results were consistent with the main analysis.*

Supplementary Table 14 Hazard ratios estimates of different blood pressure treatment target on patients (Lost to follow-up)

Outcome	Adjusted hazard ratio (95% CI)	P value
Major CVD	0.85 (0.79-0.91)	<0.01
CHD	0.81 (0.72-0.90)	<0.01
Heart Failure	0.83 (0.69-1.00)	0.06
Stroke	0.86 (0.77-0.96)	0.01
ESRD	0.79 (0.64-0.97)	0.03
Mortality	0.89 (0.80-0.98)	0.02
Serious adverse event		
Composite of seven serious adverse event	0.99 (0.94-1.05)	0.76
Hypotension	0.99 (0.76-1.29)	0.94
Syncope	0.99 (0.87-1.14)	0.94
Bradycardia	0.92 (0.52-1.62)	0.77
Electrolyte abnormality	1.42 (0.33-6.19)	0.64
Falls	1.00 (0.92-1.09)	0.97

	Acute kidney disease	0.84 (0.67-1.07)	0.16
	Dizziness	1.01 (0.92-1.10)	0.85

Note: Major CVD: composite outcomes of heart failure, chronic heart disease and stroke; CHD: chronic heart disease; ESRD: end-stage renal disease; Analyses adjusted for sex, age, smoking status, fasting glucose, high-density lipoprotein cholesterol, low-density lipoprotein cholesterol, triglyceride, total cholesterol, eGFR, Charlson Comorbidities Index, usage of ACEI/ARB, β-blocker, calcium channel blockers, diuretic; history of fall, syncope and electrolyte abnormality, Specialist, General Outpatient Clinics attendance, accident and emergency and hospitalization (within 1 year before baseline).

We have included the method of BP assessment in the revised manuscript as below:

The blood pressure readings in this data source were from attended office measurements. (Page 13, Line 257)

There was an average of 35 attended office blood pressure measurements per patient. (Page 8, Lines 136-137)

Reference:

(1) Danaei G, Rodríguez LA, Cantero OF, Logan R, Hernán MA. Observational data for comparative effectiveness research: an emulation of randomised trials of statins and primary prevention of coronary heart disease. Stat Methods Med Res. 2013 Feb;22(1):70-96.

(2) Dickerman, B.A., García-Albéniz, X., Logan, R.W. et al. Avoidable flaws in observational analyses: an application to statins and cancer. Nat Med 25, 1601–1606 (2019).

R3(7): Clarification is needed on how medication intensity and escalation are defined. It would be helpful to use a standardized score to capture treatment intensity, based on the number and dosages of antihypertensive medications. Supplementary Table 13 lacks clarity regarding the frequency and definition of intensification. Does intensification refer to the number of medications, dose increases, or changes to more potent drug classes? According to Supplementary Figure 3, most subjects reached their BP goals within two years. However, Supplementary Table 13 reports that more than 80% of patients in both arms experienced three or more treatment intensifications by year 11. This seems inconsistent and warrants clarification.

A: *Thank you very much for your comment. The original Supplementary Table 13 combined all adjustments to antihypertensive regimens, including the changes of both dosage and the number of medications. We recognize that this may not have been sufficiently clear or appropriate for presenting treatment intensification. Therefore, we have revised the table to show the proportions of treatment intensification episodes by the number of dosages increases and the number of additional antihypertensive drugs during that year, as shown below (Supplementary Table 26 in the revised manuscript).*

Supplementary Table 26. The proportion of times of intensification in each treatment strategy during that year

	Year 1	Year 2	Year 3	Year 4	Year 5	Year 6	Year 7	Year 8
Blood pressure target below 140/90 mmHg								
0	65.50	78.35	86.81	86.99	86.65	85.78	88.63	90.95
1	28.81	17.54	11.02	10.94	11.30	11.83	9.58	7.61
2	4.75	3.38	1.73	1.62	1.67	1.89	1.46	1.16

≥3	0.94	0.73	0.42	0.45	0.38	0.50	0.33	0.28
Blood pressure target below 130/80 mmHg								
0	57.56	67.23	78.29	81.74	83.32	84.43	88.09	91.31
1	33.25	25.23	17.54	15.04	13.78	12.94	9.85	7.20
2	7.55	6.02	3.38	2.60	2.36	2.13	1.68	1.20
≥3	1.64	1.52	0.79	0.62	0.54	0.50	0.38	0.29

	Year 9	Year 10	Year 11
Blood pressure target below 140/90 mmHg			
0	93.94	96.35	98.51
1	4.99	3.04	1.20
2	0.83	0.53	0.27
≥3	0.24	0.08	0.02
Blood pressure target below 140/90 mmHg			
0	94.19	96.71	98.61
1	4.76	2.69	1.12
2	0.83	0.47	0.22
≥3	0.22	0.13	0.05

To clarify on the point that most subjects reached the intensive BP goal within two years, as shown in Supplementary Figure 3, we have shown in the table that the proportions of patients who had zero drug intensification were >80% after the first two years.

Supplementary Figure 3. Systolic blood pressure and diastolic blood pressure during follow-up

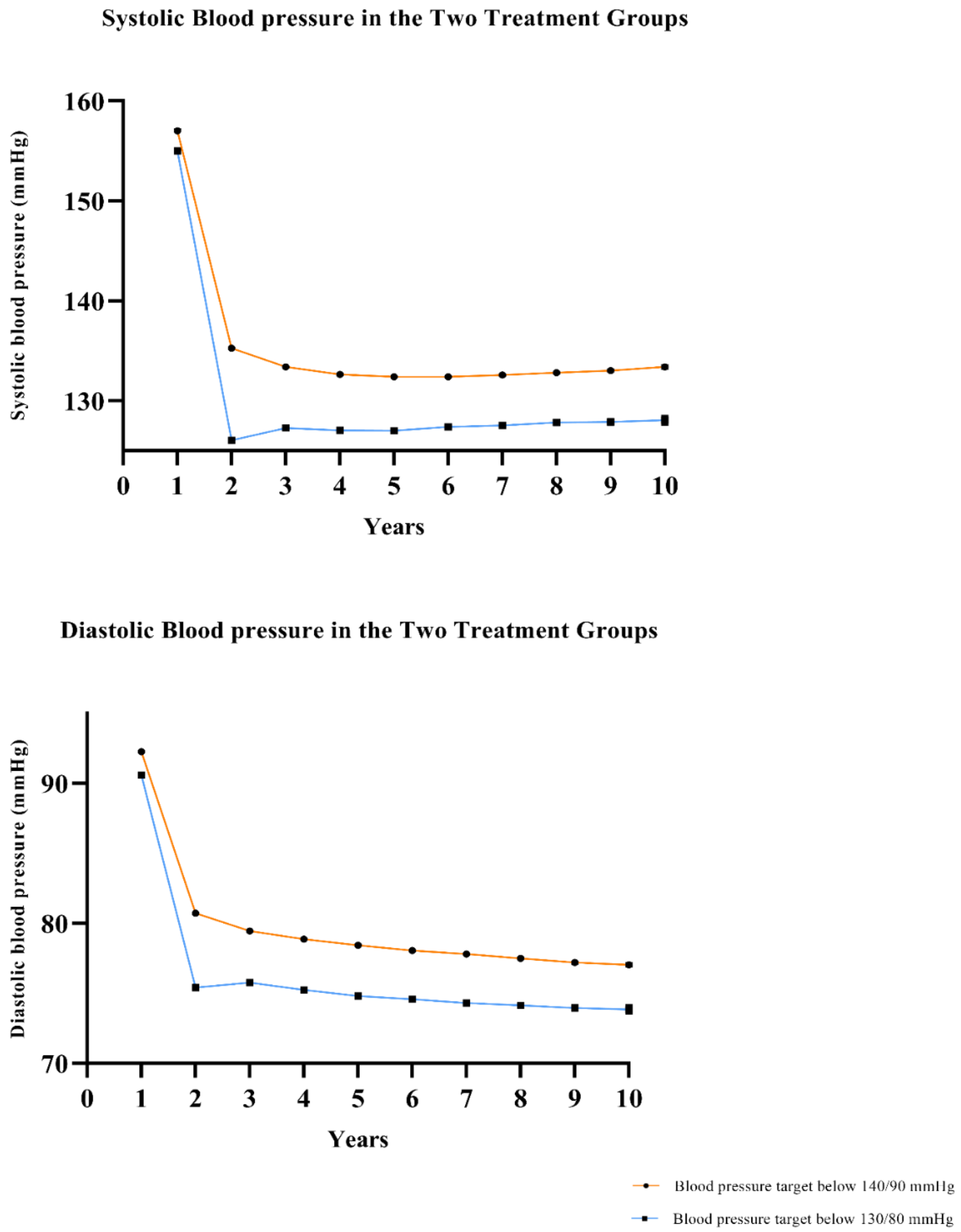

R3(8):	The lack of increased adverse events warrants more detailed discussion and analysis. Given the issues raised above, one wonders whether the study cohort enrolled relatively healthier or lower-risk individuals.
A:	*Thank you very much for your comments. Since we excluded patients with CKD, DM, or CVD, it was probable that the population included in our study were relatively healthier compared to previous RCTs (1,2), who might have lower risk of serious side effect. However, the results from the subgroup analysis of patients*

with a higher CCI score (CCI≥4) did not indicate an increased trend in the risk of adverse events, supporting the robustness of our results.

Our findings regarding the safety profile are generally consistent with those of the latest RCT conducted in China (3). Besides, the observed superior safety profile may be influenced by characteristics of the study population or the treatment strategies employed. Notably, patients with chronic kidney disease were excluded, as this group is more susceptible to acute kidney injury (4). Besides, the relatively low incidence of electrolyte disturbances could be attributed to the low prevalence of diuretics use in Chinese populations (5,6). Another reason for the low incidence of adverse events could have been caused by the inclusion criterion of events that required hospital admission. To address this, we conducted a sensitivity analysis that included all adverse events recorded in both inpatient and outpatient settings. The details regarding the results are provided in our response to comment number R2 (2). The results showed a slightly increased risk of dizziness among patients with the intensive treatment target. We have added the details in the Discussion section as below:

In contrast, we did not observe among patients managed with intensive blood pressure target of 130/80mmHg a greater risk of these adverse effects. Our findings regarding the safety profile are generally consistent with those of the latest RCT conducted in China. The low observed incidence of adverse events could be attributed partially to the selection of a healthier population. However, the results from the subgroup analysis of patients with a high Charlson Comorbidity Index (CCI) did not indicate an increased risk of adverse events, supporting the lack of risk of adverse events across a diverse patient group with varying health conditions and comorbidity statuses. The low observed incidence rates of electrolyte abnormality might be attributed to the low use of diuretics in our study population, which is consistent with the practice among other Chinese populations. Additionally, we excluded the patients with chronic kidney disease, who are more likely to develop acute kidney injury. While there was no significant increase in adverse events associated with intensified BP target <130/80 mmHg reported in our study, a careful balance of potential benefits and risks of intensive blood pressure control should be considered for individual patients. (Pages 10-11, Lines 190-201)

Reference:

(1) *SPRINT Research Group. A randomized trial of intensive versus standard blood-pressure control. New England Journal of Medicine. 2015; 373(22): 2103-2116.*

(2) *ACCORD Study Group. Effects of intensive blood-pressure control in type 2 diabetes mellitus. New England Journal of Medicine. 2010; 362(17): 1575–1585.*

(3) *Liu, JiaminAi, Xinyue et al. Lowering systolic blood pressure to less than 120 mm Hg versus less than 140 mm Hg in patients with high cardiovascular risk with and without diabetes or previous stroke: an open-label, blinded-outcome, randomised trial. The Lancet. 2024; 404: 245 – 255*

(4) *Rocco, MV · Sink, KM · Lovato, LC · et al. Effects of intensive blood pressure treatment on acute kidney injury events in the systolic blood pressure intervention trial (SPRINT). Am J Kidney Dis. 2018; 71:352-361*
(5) *Suchard, MA · Schuemie, MJ · Krumholz, HM · et al. Comprehensive comparative effectiveness and safety of first-line antihypertensive drug classes: a systematic, multinational, large-scale analysis. Lancet. 2019; 394:1816-1826*
(6) *Yang, R · Tang, J · Zhuo, Y · et al. Current prescription status of antihypertensive drugs in Chinese patients with hypertension: analysis by type of comorbidities. Clin Exp Hypertens. 2022; 44:240-248*

R3(9): Why so few medications from standard drug classes? Were those in intensive arm on more meds than those in standard arm? (e.g., similar to SPRINT, ~2.8 vs. 1.8)

A: *Thank you very much for your comments. The table below summarizes the mean number of anti-hypertensive medications used by patients in each arm over the follow-up years.*

Supplementary Table 27. The mean numbers of anti-hypertensive regimens in each treatment strategy over the follow-up years

	Year 0	Year 1	Year 2	Year 3	Year 4	Year 5	Year 6	Year 7	Year 8	Year 9	Year 10	Year 11
Blood pressure target below 140/90 mmHg												
Mean /SD	1.21/ 0.44	1.33/ 0.53	1.35/ 0.54	1.37/ 0.55	1.38/ 0.56	1.39/ 0.58	1.42/ 0.59	1.45/ 0.61	1.49/ 0.64	1.55/ 0.67	1.61/ 0.68	1.66/ 0.69
Blood pressure target below 130/80 mmHg												
Mean /SD	1.20/ 0.44	1.34/ 0.54	1.43/ 0.58	1.47/ 0.61	1.51/ 0.62	1.53/ 0.64	1.57/ 0.66	1.61/ 0.67	1.66/ 0.69	1.72/ 0.71	1.79/ 0.73	1.86/ 0.75

As expected, patients in the lower blood pressure target group received a greater number of classes of antihypertensive drugs compared to those in the traditional treatment target group. However, given effective pharmacological intervention on the management of blood pressure relies on both the adjustment of medication types and dosages, the slightly lower number of medications from the intensive treatment arm may not fully reflect the intensity of treatment prescribed.

As shown in the Supplementary Figure 3, which summarizes blood pressure values over the follow-up years across study arms, the majority of patients achieved the intensive blood pressure target within the first two years. This is evidenced by a higher proportion of patients undergoing more than two treatment intensifications during this initial period compared to the remainder of the follow-up as shown in the Supplementary Table 26. Consequently, the need for additional antihypertensive medications was reduced thereafter. The details of the figures and tables are provided in comment number R3 (7).

Furthermore, it is important to note that the intensive treatment target in the SPRINT trial was defined as 120 mmHg, whereas the target in our current study was 130 mmHg. As a result, participants in the SPRINT trial were expected to receive more aggressive antihypertensive therapy. Therefore, direct comparisons of the number of antihypertensive medications between the two studies may not be appropriate.

R3(10):	It would be useful to present the results of sensitivity analyses, model specifications (including variables used and regression coefficients), and the impact of weight truncation on outcomes.
A:	*Thank you very much for your suggestions. We have added those information as supplementary tables. The results of sensitivity analyses are summarized in supplementary table 11-25, the model specifications are summarized in supplementary table 28, and the impact of weight truncation on outcomes is summarized in supplementary table 4.*

R3(11):	For CONSORT diagram, in Figure 1, the ineligible patients + eligible patients (N=118,721) do not add up to N=699,568. Please explain this discrepancy.
A:	*Thank you very much for your comments. We have double checked the numbers and the numbers now add up to the total N.*

Reviewer 4

R3:	I co-reviewed this manuscript with one of the reviewers who provided the listed reports. This is part of the Nature Communications initiative to facilitate training in peer review and to provide appropriate recognition for Early Career Researchers who co-review manuscripts.
A:	*Thank you very much for your comments.*

RE: NCOMMS-25-28778A Optimal blood pressure target for antihypertensive management in patients with uncomplicated hypertension: Real-world evidence from a target trial emulation study

On behalf of the co-authors, we would like to thank the reviewers for their further comments and suggestions. In response, we have made the necessary adjustments in the revised manuscript, and hope that you will now find it suitable for publication. We provide here, our point-to-point responses to the earlier version of this paper reviewed.

Reviewer 1

R1 (1)	The authors answered most of the questions, and the manuscript improved. In my opinion, some minor changes are needed.
A:	Thank you very much for your comments and please see our point-by-point response below.

R1 (2)	Abstract: there are some typos (also in the text).
A:	Thank you for the comments. We have carefully proof-read the manuscript to revise the typos shown in the abstract and main text.

R1 (3)	Introduction: At least in my opinion, it would be more appropriate to list references from 5 to 10 in order of their appearance in the literature (8-10-9-7-6-5).
A:	Thank you very much for your suggestions. We have reordered the references based on their appearance in the literature.

R1 (4)	Abstract: "The absolute risk reduction observed for major CVDs was -0.44 % (CI, -1.47 to -0.44 %), and for all cause was -0.84 % (CI, -1.17 to -0.52 %).", and Results: "The risk reduction (Table 2) was observed for major CVD (absolute risk difference, -0.95 % [CI, -1.47 to -0.44 %]), and all-cause mortality (absolute risk difference, -0.84 % [CI, -1.17 to -0.52 %])." Please clarify.
A:	Thank you very much for your comments. We apologized the mistakes on the typos for the absolute risk difference we mentioned in the previous abstract and main text. We have double checked and revised the typos shown in the previous abstract and main text based on the results shown in Table 2:<br>(1) In the abstract: Results: Of the 118,271 eligible patients in our study, 82,753 patients were managed for the traditional treatment target of <140/90mmHg and 15,992 patients managed for the lower treatment goal of <130/80mmHg. Over the 7-years of follow-up, patients in the lower blood pressure target group were observed to incur a lower risk of hypertension related complications including CVDs (hazard ratio [HR]: 0.85 [95%CI, 0.79 to 0.91]), and all-cause mortality (hazard ratio [HR]: 0.88 [95%CI, 0.80 to 0.97]), with no significant increase risk of the adverse events reported. The absolute risk reduction observed for major CVDs was -0.58 % (CI, -0.63 to -

	0.53 %), and for all-cause mortality was -0.60 % (CI, -0.62 to -0.57 %). (Page 4, Lines 72-77)<br><br>(2) In the Results section: Over an average follow-up of 7 years, patients with lower BP target were observed to incur a lower risk of hypertension related complications (Figure 2), including major CVD [HR (95%CI):0.85 0.79-0.91)], CHD [HR (95%CI): 0.81 (0.70-0.90)], stroke [HR (95%CI): 0.79 (0.64-0.97)], and all-cause mortality [HR (95%CI): 0.88 (0.80-0.97)]. The risk reduction (Table 2) was observed for major CVD (absolute risk difference, -0.58 % [95%CI, -0.63 to -0.53 %]), and all-cause mortality (absolute risk difference, -0.60 % [CI, -0.62 to -0.57 %]). There was an average of 35 attended office blood pressure measurements per patient. (Pages 7-8, Lines 128-135)

R1 (5)	You report: “Table 2 Hazard ratios estimate of different blood pressure treatment targets on the risk of hypertension related complications”. HRs do not appear to be listed in this table. Please check.
A:	Thank you very much for your comments. We have changed the title into ‘Table 2 Crude incidence rate and adjusted risk difference of different blood pressure treatment targets on the risk of hypertension related complications and serious adverse events.’.

R1 (6)	You report: “Our findings regarding the safety profile are generally consistent with those of the latest RCT conducted in China.13” Is this reference correct?
A:	Thank you very much for your comments. We have revised it into the correct reference in the manuscript: Our findings regarding the safety profile are generally consistent with those of the latest RCT conducted in China.[12] (Page 10, Lines 188-189)<br><br>*Reference:*<br>12. Liu, JiaminAi, Xinyue et al. Lowering systolic blood pressure to less than 120 mm Hg versus less than 140 mm Hg in patients with high cardiovascular risk with and without diabetes or previous stroke: an open-label, blinded-outcome, randomised trial. The Lancet. 2024; 404: 245 – 255.

Reviewer 2

R2 (1)	The authors have made considerable efforts to address the reviewers' comments. However, several important concerns remain:
A:	Thank you for your helpful comments and suggestions. Please find our response below.

R2 (2)	After adjusting for competing risks, the association between intensive BP control and reduced risk of major CVD was attenuated, and it became non-significant for stroke

	and ESRD.<br><br>The inclusion of outpatient diagnoses revealed a significantly higher risk of syncope and dizziness associated with intensive treatment.<br><br>These findings are not consistent with the results of the main analysis, and therefore do not support the conclusion that an intensive BP target is associated with a lower risk of hypertension-related complications without a significant increase in adverse events.
A:	Thank you for your valuable insights regarding the interpretation of the results after adjusting for the competing risk of non-CVD death. Although the association between intensive treatment and the risk of major CVD was attenuated, the risk remained significantly lower in patients under the intensive treatment target. Similarly, for outcomes such as ESRD and stroke, the risk was still lower in the intensive group, although these differences did not reach statistical significance. One potential reason for the attenuated reduced risk is the limited number of the events of outcomes, which resulted in a lack of statistical power and a wide confidence interval. Besides, in our dataset, only the primary cause of death was recorded, which may lead to potential misclassification of the cause of death in this study. Moreover, the results from several other sensitivity analyses were mostly consistent in showing the reduced risk of the hypertension-related complications, indicating the robustness of our findings from the main analysis. Therefore, our conclusion that a lower BP target is associated with a reduced risk of hypertension-related complications remains supported by the findings. However, we have acknowledged that further studies with more precise and comprehensive cause-of-death data are necessary to better understand the relationship between intensive blood pressure management and related complications in the limitation section in the revised manuscript:<br>*Furthermore, the in our dataset, only the primary cause of death was recorded, which may lead to potential misclassification of the cause of death when considering the competing risks of the non-CVD mortality in this study. Further studies with more precise and comprehensive cause-of-death data are necessary to better understand the relationship between intensive blood pressure management and related complications.* (Pages 12-13, Lines 238-243).<br><br>Our conclusion on the null association in risk in side effects from intensive blood pressure monitoring was based on findings from the main analysis focusing on serious adverse events. Symptoms such as dizziness and syncope were generally mild; however, instances necessitating hospital admission may indicate a more severe or potentially life-threatening manifestation of these conditions. Accordingly, our main analysis prioritized events that required inpatient care, reflecting on the severity of the condition. Moreover, dizziness or syncope observed in outpatient settings may be attributable to other common comorbidities in elderly populations, such as neurological disorders, vision problems, dehydration, and low blood sugar, etc. However, in response to the concerns raised, we have revised the discussion to underscore that these findings highlight the importance of weighing both the benefits and potential risks of intensive blood pressure control when determining

	individualized treatment targets in clinical practice in the discussion section in the revised manuscript:<br>*While there was no significant increase in the risk of serious adverse events in inpatient settings observed in patients treated with intensive BP target <130/80 mmHg, the marginal increased risk of syncope and dizziness that observed in the outpatient consultation implicated the possibility of mild symptoms of these adverse effect and that careful assessment of potential benefits and risks of intensive blood pressure control should be considered for individual patients in clinical settings. Future study using more detailed and systematic adverse events monitoring are needed to better evaluate the safety of the intensive blood pressure target.* (Page 11, Lines 197-203)<br><br>Overall, based on the current results and your insightful suggestions, we have revised our study conclusion to ensure the accurate description on the findings of this study:<br>*The findings of the study provided comprehensive real-world evidence supporting the association of the clinical benefits of reduced risk of hypertension related cardiovascular complications and all-cause mortality with an intensive blood pressure management target among patients with uncomplicated hypertension without significant increased risk of serious adverse events. Nevertheless, the optimal blood pressure management target should also take into consideration of individual's characteristics, comorbidities, and potential risk factors for associated adverse events.* (Page 13, Lines 248-254)

Reviewer 3

R3 (1)	In this revised manuscript, Zhang et al. report the use of target trial emulation methodology is appropriate and timely. The investigators should be commended for including a large sample size with an extended follow-up period of up to 7 years, providing robust real-world evidence.<br>The authors provided a detailed explanation of the time-zero definition and grace period rationale. They further conducted sensitivity analyses (6-month grace period, single record, 3 consecutive records), which showed consistent results. The provided-reported BP treatment goal was unavailable in the database and is now reported as a limitation of the study.<br><br>We also commend the authors' effort in demonstrating the enrollment distribution of BP targets, which showed consistent proportions across years (2008-2012). This suggests that there is no temporal bias in treatment assignment.<br><br>Regarding the censoring criteria that use 5 consecutive BP records for defining deviation from the assigned treatment protocol, the authors conducted sensitivity analyses using 2, 3, 4, and 6 consecutive records as the censoring criteria which showed consistent results. In terms of the rigid censoring rules that may violate the positivity assumption for vulnerable patients, the authors performed sensitivity analysis considering age, frailty, and polypharmacy as contraindications, and again the results remained consistent. Given that each individual is unique and statistical

	aggregates should not override clinical acumen, the authors adequately addressed this concern and acknowledged the limitation in the Discussion.<br><br>The authors emphasized the use of the "Clone-Censor-Weight" approach to address immortal time bias, selection bias, and confounding variables, but fundamentally, the study infers treatment strategy (intensive versus standard) from downstream observations (BP readings + medication changes) rather than from actual clinical intent of the physician. Hence, the classification rules may not capture actual physician treatment goal. This is acknowledged as a limitation.<br><br>Some persistent concerns include the lack of generalizability, as the study excluded patients with diabetes, CKD, and CVD, which limits applicability to real-world hypertensive patients. Nonetheless, the authors did acknowledge this limitation and provided a sensitivity analysis based on a generalized morbidity index. The study provides valuable real-world evidence, and the study results should be interpreted with appropriate caution by the reader.<br><br>We have no additional major or minor concerns.
A:	Thank you for the valuable feedback and support on the revision made in response to the earlier comments.

We sincerely hope that you will consider this revised manuscript favorably. Should there be further corrections or clarifications needed, we are more than happy to make the necessary changes and provide additional information. We look forward to hearing from you soon.

Yours sincerely,

Eric Yuk Fai Wan
Department of Family Medicine and Primary Care &
Department of Pharmacology and Pharmacy
The University of Hong Kong
Email: yfwan@hku.hk
Tel. (852) 2552 4690
